# A deep-learning approach to predict reproductive toxicity of chemicals using communicative message passing neural network

**DOI:** 10.3389/ftox.2025.1640612

**Published:** 2025-07-22

**Authors:** Owen He, Daoxing Chen, Yimei Li

**Affiliations:** ^1^ Deerfield Academy, Deerfield, MA, United States; ^2^ School of Pharmaceutical Sciences, Wenzhou Medical University, Wenzhou, China; ^3^ Department of Biostatistics, St. Jude Children’s Research Hospital, Memphis, TN, United States

**Keywords:** reproductive, artificial intelligence (AI), deep learning, graph neural network, CMPNN, *in silico*

## Abstract

Reproductive toxicity is a concern critical to human health and chemical safety assessment. Recently, the U.S. Food and Drug Administration announced plans to assess toxicity with artificial intelligence-based computational models instead of animal studies in “a win-win for public health and ethics.” In this study, we used a reproductive toxicity dataset using Simplified Molecular Input Line Entry Specifications (SMILES) to represent 1091 reproductively toxic and 1063 non-toxic small-molecule compounds. A repeated nested cross-validation procedure was applied, in which the dataset was randomly partitioned into five distinct folds in the outer loop, each time, one fold serving as the test set. In the inner loop, a similar procedure was also repeated five times, with 12.5% each time serving as the validation set. We first evaluated the performance of classical machine learning (ML) methods such as Random Forest and Extreme Gradient Boosting on predicting reproductive toxicity, using standard model evaluation metrics including accuracy score (ACC), the area under the curve (AUC) of the receiver operating characteristics curve (ROC) and F1 score. Our analyses indicate that these methods’ overall results were mediocre and insufficient for high-throughput screening. To overcome these limitations, we adopted the Communicative Message Passing Neural Network (CMPNN) framework, which incorporates a communicative kernel and a message booster module. Our results show that our ReproTox-CMPNN model outperforms the current best baselines in both embedding quality and predictive accuracy. In independent test sets, ReproTox-CMPNN achieved a mean AUC of 0.946, ACC of 0.857 and F1 score of 0.846, surpassing traditional algorithms to establish itself as a new state-of-the-art model in this field. These findings demonstrate that CMPNN’s deep capture of multi-level molecular relationships offers an efficient and reliable computational tool for rapid chemical safety screening and risk assessment.

## 1 Introduction

Reproductive toxicity, referring to the ability to disturb reproductive competence through structural and functional alterations (U.S. Food and Drug Administration, 2011), remains a concern critical to chemical safety assessment, human health and development of novel drugs. Such toxicity can lead to a wide range of adverse effects (U.S. Environmental Protection Agency, 1996), including reduced fertility due to hormonal disruptions, decreased sperm count and motility or impaired ovarian reserves, harm to embryonic or fetal development leading to elevated rates of failure to reach term (about 50% for conceptions and 32%–34% for early pregnancies), congenital malformations or growth or neurobehavioral disorders like low birth weight, hypospadias or irregular puberty onset. These adverse outcomes arise from diverse mechanisms at multiple stages. Endocrine disruption occurs when toxins such as phthalates mimic or block endogenous hormones, destabilizing endocrine systems; oxidative stress can disrupt cellular signaling and induce apoptosis, impairing spermatogenesis and oocyte maturation. Genotoxic effects on germline or embryonic cells, including mutations and chromosomal abnormalities, elevate risks of developmental defects and early pregnancy loss. During embryogenesis, toxic exposures may interfere with implantation, organ formation, and morphogenesis, culminating in teratogenic outcomes and postnatal developmental deficits. Exposure-triggered epigenetic alterations, such as DNA methylation, histone modifications, and miRNA expression changes, may persist through embryonic development, potentially affecting multiple generations of offspring. During important windows of development like gametogenesis, pregnancy or early childhood, exposure to reproductive toxicants can cause irreversible adverse outcomes (Archibong et al., 2018; Iavarone and Dasmahapatra, 2025; Shi et al., 2021; Yang et al., 2018).

Through the aforementioned mechanisms, environmental and industrial chemicals from everyday plastics to persistent pollutants have been increasingly implicated in reproductive complications. Phthalates, widely used as plasticizers, interfere with androgen signaling, impairing spermatogenesis and ovary function; in animal studies, exposure was linked to short anogenital distances and reproductive tract malformations. Bisphenol A (BPA), a ubiquitous xenoestrogen, disrupts the hypothalamic-pituitary-gonadal axis, decreasing fertility in both sexes, and impairs embryonic implantation. Pesticides, particularly those with endocrine-modulating or genotoxic properties, are known to delay puberty, reduce gamete quality and increase miscarriage risk. Heavy metals like lead and cadmium induce oxidative stress in gonadal tissues, leading to diminished sperm production, menstrual irregularities, and fetal growth restriction. Per- and polyfluoroalkyl substances (PFAS)—persistent “forever chemicals”—cross the placenta, disrupt hormone pathways, impair ovarian and testicular development, and have been associated with reduced birth weight and infertility (Yesildemir and Celik, 2024; Mínguez-Alarcón et al., 2023).

Recent research has shown that many chemicals used in workplaces have not been adequately studied on their possible reproductive toxicity despite previous reports that exposure to these chemicals increases risk of endocrine disruption, impaired fertility and adverse reproductive outcomes (Rim, 2017). In electronics factories, workers routinely handle solvents such as trichloroethylene (TCE) and perchloroethylene (PCE), volatile organic compounds, as well as phthalate-rich plasticizers and flame retardants like polybrominated diphenyl ethers (PBDEs) to which they are exposed via inhalation and skin contact with cables and casings. In textile manufacturing, workrooms are often laden with bleaching agents, azo dyes, formaldehyde, and heavy metals released as airborne dust or absorbed dermally, leading to potential miscarriages, menstrual disturbances, and hormone disruption. Moreover, in poorly ventilated settings, microfibers and volatile processing chemicals that increase oxidative and endocrine stress can be inhaled (Wang and Qian, 2021). Even low levels of exposure to toxicants during pregnancy can lead to maternal complications, birth defects and delays or disorders in childhood development (Di Renzo et al., 2015). A well-known example is thalidomide, which has caused thousands of miscarriages and stillbirths in addition to almost 10,000 severe limb malformations at birth (Miller, 1991). Therefore, thorough evaluation of reproductive toxicity is required both for public health policy safeguarding current and future generations’ wellbeing as well as for the development of novel drugs and regulatory requirements.

Besides health impairments to immediately affected individuals, widespread exposure to reproductive toxicants brings substantial increases in healthcare costs and long-term burdens on public health systems. Njagi et al. (2023) reported that approximately 17.5% of adults globally experience infertility and that a recent Global Burden of Disease analysis estimated over 110 million cases of female infertility in 2021—a rise of 84% since 1990. Exposure to endocrine-disrupting chemicals, heavy metals, and persistent organic pollutants has additionally been linked to increased incidence of reproductive cancers, such as testicular and ovarian cancer, and a higher prevalence of birth defects (e.g., neural tube defects, hypospadias, congenital heart anomalies). Growing evidence also supports that prenatal or parental exposure to toxicants like methoxychlor and polychlorinated biphenyls has been linked to impaired fertility and reproductive health across multiple generations (Liu et al., 2025; Pan et al., 2023; Brehm and Flaws, 2019). According to a comprehensive analysis (Attina et al., 2016), by 2016, the estimated annual cost of healthcare in the United States had amounted to over 340 billion USD, or more than 2.3% of GDP, due to low-level daily exposure to endocrine-disrupting chemicals potentially hazardous to reproduction. Together, these trends underscore an urgent need for improved reproductive health surveillance and regulatory intervention.

With a global cost of approximately 10.6 billion USD in 2022, a figure expected to rise to $25.7 billion by 2032 (Faizullabhoy and Kamthe, 2022), reproductive toxicity testing is essential to the development of novel drugs. This cost indicates that traditional *in vitro* and *in vivo* tests of toxicity remain expensive and time-consuming; they additionally raise ethical issues regarding animal use. On 10 April 2025, the FDA announced plans to replace animal studies with artificial intelligence (AI)-based computational models to assess drug toxicity (Office of the Commissioner, 2025), in “a win-win for public health and ethics.” The European Union’s Registration, Evaluation, Authorisation, and Restriction of Chemicals regulation (REACH) and the U.S. Environmental Protection Agency (EPA)’s Toxic Substances Control Act (TSCA) now require not only extensive hazard assessments but also explicit justification for the use of animal testing, effectively making computational models a regulatory necessity. Under REACH’s “last-resort” provision, animal testing can only be pursued when alternative methods, including *in silico* approaches, have been exhausted. Similarly, TSCA encourages the use of predictive exposure and fate models to fill data gaps in chemical assessments to reduce reliance on new animal studies (European Commission, 2022; United States Environmental Protection Agency, 2025). As these regulations continue to expand their scope, development of robust computational toxicology models is no longer optional but essential to meet global compliance while minimizing ethical and financial burdens.

Quantitative Structure–Activity Relationship (QSAR) models use mathematical methods to model relationships between chemical structures’ properties and biological activities in order to predict biological activities of novel chemicals before formal experiments. These models provide a more ethical, cost-effective, rapid and efficient alternative to traditional *in vitro* and *in vivo* tests. Until the 1990s, QSAR models used simple linear and partial least squares regressions along with simple 1-D descriptors representing chemical structures. Since the early 2000s, QSAR models have evolved to incorporate machine learning (ML) methods such as Random Forest (RF), Support Vector Machines (SVM), and Extreme Gradient Boosting (XGBoost). These methods employ 2-D and 3-D descriptors, which more accurately capture molecular properties and non-linear relationships between properties and bioactivities (Cortes and Vapnik, 1995; Breiman, 2001; Sheridan et al., 2016; Li et al., 2025; Bahia et al., 2023; Xu et al., 2012; Ballester and Mitchell, 2010; Wu and Wang, 2018). These advancements resulted in more robust models and increased accuracy of predictions.

However, classical ML models rely on pre-computed descriptors that remain fixed throughout the training process, possibly limiting their performance. In the last 15 years, Deep Learning (DL) methods, particularly graph convolution-based Graph Neural Network (GNN), have been introduced to QSAR modeling (Wang et al., 2023; Duvenaud et al., 2015; Kearnes et al., 2016). In GNN models or in general, Message Passing Neural Network (MPNN), molecules are presented as undirected graphs with atoms as nodes and bonds as edges. The message passing phase of MPNN captures dynamic interactions between atoms and bonds; aggregated messages that represent whole molecules are then used to predict bioactivities through readout functions (Gilmer et al., 2017). Different from the node-based message passing phase of MPNN, Directed MPNN (DMPNN) considers directions of edges that better differentiate the influence between nodes, reducing redundancy in message passing (Dai et al., 2016; Yang et al., 2019; Han et al., 2022; Xia et al., 2023). Yang et al. showed that DMPNN outperformed most other deep neural network methods in predicting molecular bioactivities (Yang et al., 2019). More recently, the Communicative Message Passing Neural Network (CMPNN) framework, employing a communicative kernel to reinforce message exchange between nodes and edges and incorporates a message booster module during message passing to enrich molecular graph embeddings has demonstrated an enhanced predictive performance compared to DMPNN (Song et al., 2021). While all GNN methods offer a more dynamic and data-driven approach to developing bioactivity prediction models that allows for automatic extraction of features from molecular graphs, thus requiring less expertise for more accurate predictions of bioactivity, helping to enhance high-throughput screening processes and accelerate drug development timelines, recent research has reported significant gains of CMPNN in different molecular property prediction tasks (Rao et al., 2022; Song et al., 2021; Liu et al., 2024).

Recently in the specific area of predicting reproductive toxicity, Basant et al. (Basant et al., 2016) utilized two ensemble machine learning models, Decision Tree Forest and Decision Tree Boost, based on 334 chemicals of which toxicity to rats is known, to demonstrate the effectiveness of ML-based QSAR models. Further work based on larger datasets with more than 1,500 chemicals and methods including frequentist approaches (Jiang et al., 2019; Feng et al., 2021), Bayesian methods (Zhang et al., 2020) and Graph Transformer Networks (Ren et al., 2024), achieved areas under the receiver operating characteristic curve (AUCs) close or greater than 0.900 and accuracy scores (ACC) of at least 0.830.

Our study is to develop better-performing *in silico* predictive models on reproductive toxicity using a larger dataset of 2,154 chemicals that contains Simplified Molecular Input Line Entry Specifications (SMILES) and a binary classification of reproductively toxic or non-toxic. Considering the performance of CMPNN method in other areas, we compared it with 11 ML models using standard model evaluation metrics including accuracy score (ACC), the area under the curve (AUC) of the receiver operating characteristics curve (ROC), F1 score, balanced accuracy (BA), Cohen’s Kappa and Matthews correlation coefficient (MCC).

The rest of the manuscript is organized as follows. In Section 2, the reproductive toxicity dataset and the overall process are described first, followed by a brief description of different models and metrics of model evaluation. In Section 3, information about hyperparameters, results, and comparisons are presented. The manuscript ends with a conclusion and description of future work in Section 4.

## 2 Materials and methods

### 2.1 Toxicity data preparation

The reproductive toxicity dataset assembled in this study includes 2,154 small-molecule compounds from the ECHA-C&L Inventory, OECD-eChemPortal and previous literature (Ren et al., 2024; Feng et al., 2021; Zhang et al., 2020, Jiang et cal. 2019). The lists of chemicals are shown in Supplementary Table S1. This dataset includes a varied mix of chemical types—industrial substances, environmental pollutants, and pharmaceuticals. In the European Chemicals Agency(ECHA) database, substances receive Category 1A (known human toxicant), 1B (presumed), or 2 (suspected) based on a weight-of-evidence approach incorporating epidemiological, *in vivo*, *in vitro*, and structural-activity data—excluding effects merely secondary to general toxicity (European Chemicals Agency, 2017). Likewise, The Organisation for Economic Co-operation and Development (OECD)- eChemPortal aggregates reproductive hazard classifications (1A/1B/2 and lactation effects) from national and international regulatory sources (OECD, 2008; 2023). Most of the underlying data were derived from rodent studies measuring endpoints like fertility rates, implantation success, offspring development, and congenital malformations, with human evidence primarily influencing Category 1A. Therefore, this dataset provides a good basis for distinguishing reproductively toxic from non-reproductively-toxic compounds to develop computational methods.

### 2.2 Calculation and construction of molecular fingerprints

This study was conducted in a Python 3.12.2 environment installed with RDKit 2025.3.2, Chemprop 2.1.0, PyTorch 2.7.1, scikit-learn 1.6.1, Pandas 2.2.2, and NumPy 1.26.4. SMILES strings of the 2,154 chemicals were converted with the standard SMILES parser into RDKit molecular objects. Morgan fingerprints (ECFP4) were generated with a radius of 2 and a total length of 2,048 bits in binary (presence/absence) mode, producing ExplicitBitVect-type representations that provided a reliable data foundation for subsequent classification modeling.

### 2.3 Classical machine learning methods

This study used eleven classical machine learning methods to predict chemical compounds’ reproductive toxicity. Decision Tree constructs an interpretable tree by recursively splitting on feature thresholds, k-Nearest Neighbors is a “lazy” learner that assigns class based on the majority vote of nearest training samples in Euclidean space, Linear SVM finds a maximum-margin hyperplane for linear separation, Naive Bayes applies Bayes’ theorem under a conditional feature-independence assumption, Logistic Regression fits a sigmoid function to estimate class probabilities, Random Forest ensembles multiple decision trees built on random features and sample subsets to reduce overfitting, Adaptive Boosting (AdaBoost) trains weighted weak learners iteratively and combines them into a strong classifier, Gradient Boosted Decision Tree (GBDT) builds learners sequentially by fitting residual errors, Extra Trees further randomizes split thresholds and features to increase model diversity, Light Gradient Boosting Machine (LightGBM) employs a leaf-wise tree growth strategy for faster training, and XGBoost uses second-order derivative information and regularization to optimize both speed and accuracy. To ensure fair comparison and reproducibility, all models were tuned and evaluated within a unified pipeline. Algorithm implementation and key hyperparameters are shown in Supplementary Table S2.

### 2.4 Communicative message passing neural network (CMPNN)

**FIGURE 1:**
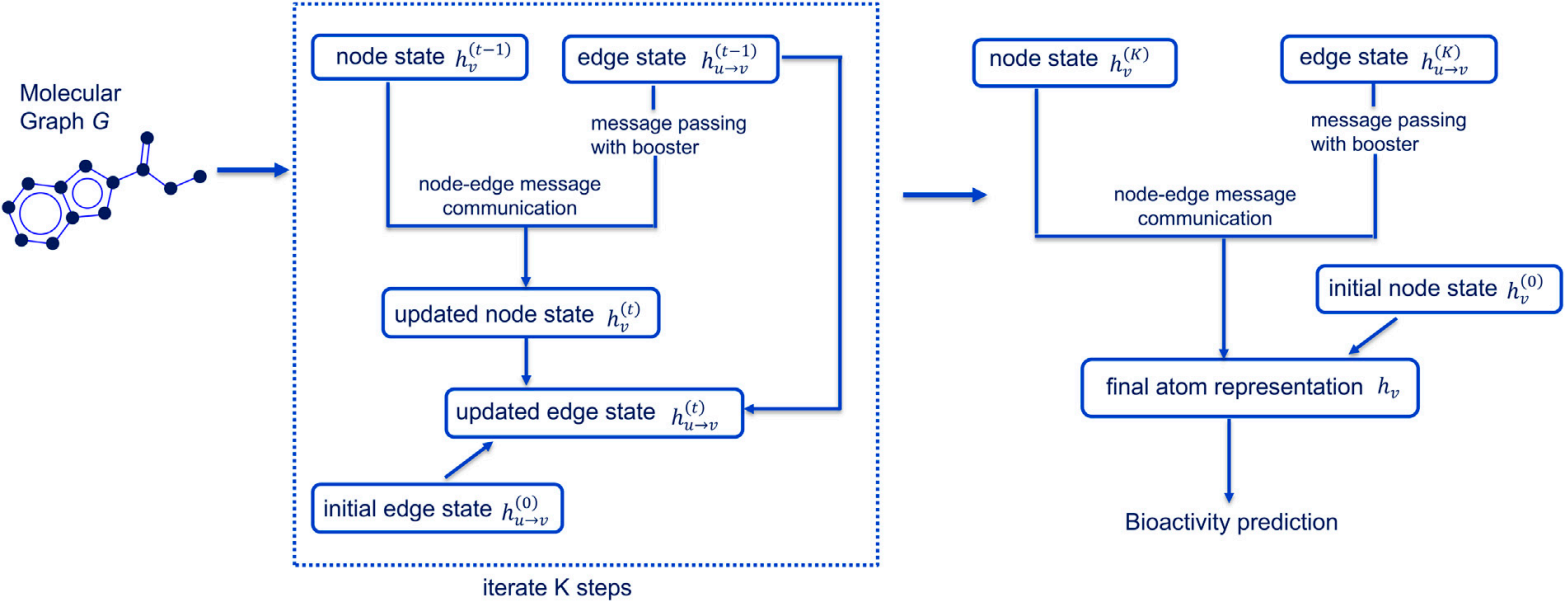
Architecture of CMPNN model.

In this study, we selected the CMPNN framework (Figure 1), given its architecture of enhanced message exchange and boosting, to develop our reproductive toxicity prediction model (ReproTox-CMPNN) and compared it with machine learning methods.

To provide a more formal and precise description, we decomposed CMPNN into five steps, each with its governing equations.

#### 2.4.1 Graphical representation

Each molecule is represented as a directed graph

$$G=\left(V,E\right),$$

where V is the set of atoms (nodes) and E is the set of directed bonds (edges). Each atom V has an initial feature vector $h_{v}^{\left(0\right)}$ ​, and each directed edge ( u → v ) has an initial embedding $h_{u\to v\;}^{\left(0\right)}$ , both obtained from atom- and bond-level descriptors.

#### 2.4.2 Message passing

Over K iterations, node states are updated by aggregating incoming edge messages:

$$m_{v}^{\left(t\right)}=\sum_{u:\left(u\to v\right)\in E}h_{u\to v\;}^{\left(t-1\right)}for\;t=1,...,K.$$

The new node hidden state is then

$$h_{v}^{\left(t\right)}={MLP}_{node\;}\left(\left[h_{v\;}^{\left(t-1\right)}\left\|m_{v}^{\left(t\right)}\right\|b_{v}^{\left(t\right)}\right]\right),$$

where “‖” denotes concatenation and $b_{v}^{\left(t\right)}$ ​ is the message-booster (Section 2.4.3).

#### 2.4.3 Message booster

To amplify the most informative bond contributions, for each node v we compute

$$b_{v}^{\left(t\right)}=\max_{u:\left(u\to v\right)\in E}\;h_{u\to v\;}^{\left(t-1\right)}\;.$$

(element-wise maximum over its incoming edge embeddings). We then scale the aggregated message by element-wise multiplication:

$$m_{v}^{{\left(t\right)}^{\prime }}=m_{v}^{\left(t\right)}⊙b_{v}^{\left(t\right)},$$

and use $m_{v}^{{\left(t\right)}^{\prime }}$ ​ in place of $m_{v}^{\left(t\right)}$ in the node update above.

#### 2.4.4 Node-edge communication

Edge embeddings are updated based on the newly computed node states. For each directed bond ( u → v ),

$$h_{u\to v}^{\left(t\right)}=h_{u\to v}^{\left(t-1\right)}+ReLU\left(W_{e\;}\left[h_{v}^{\left(t\right)}\left\|\;\right.h_{u\to v}^{\left(t-1\right)}\;\right]\right),$$

where $W_{e\;}$ is a learned weight matrix and ReLU is a rectified linear unit used at the next iteration. This residual formulation allows atom and bond representations to co-evolve.

#### 2.4.5 Readout and prediction head

After the final iteration t = K, we obtain node embeddings $\left\{h_{v}^{\left(K\right)}\right\}$ . We then apply a gated recurrent unit (GRU) readout to each node (to capture ordering effects), and sum over all nodes to produce a fixed-length molecular vector:

$$h_{mol}=\sum_{v\in V}GRU\;\left(h_{v}^{\left(K\right)}\right).$$

Finally, a two-layer perceptron with dropout maps $h_{mol}$ to the toxicity probability:

$$\hat{y}=\sigma \;\left(W_{2\;}ReLU\left(W_{1\;}h_{mol}+b_{1}\right)+b_{2}\right),$$

where σ is the sigmoid activation.

### 2.5 Model training, hyperparameter optimization and evaluation

**FIGURE 2:**
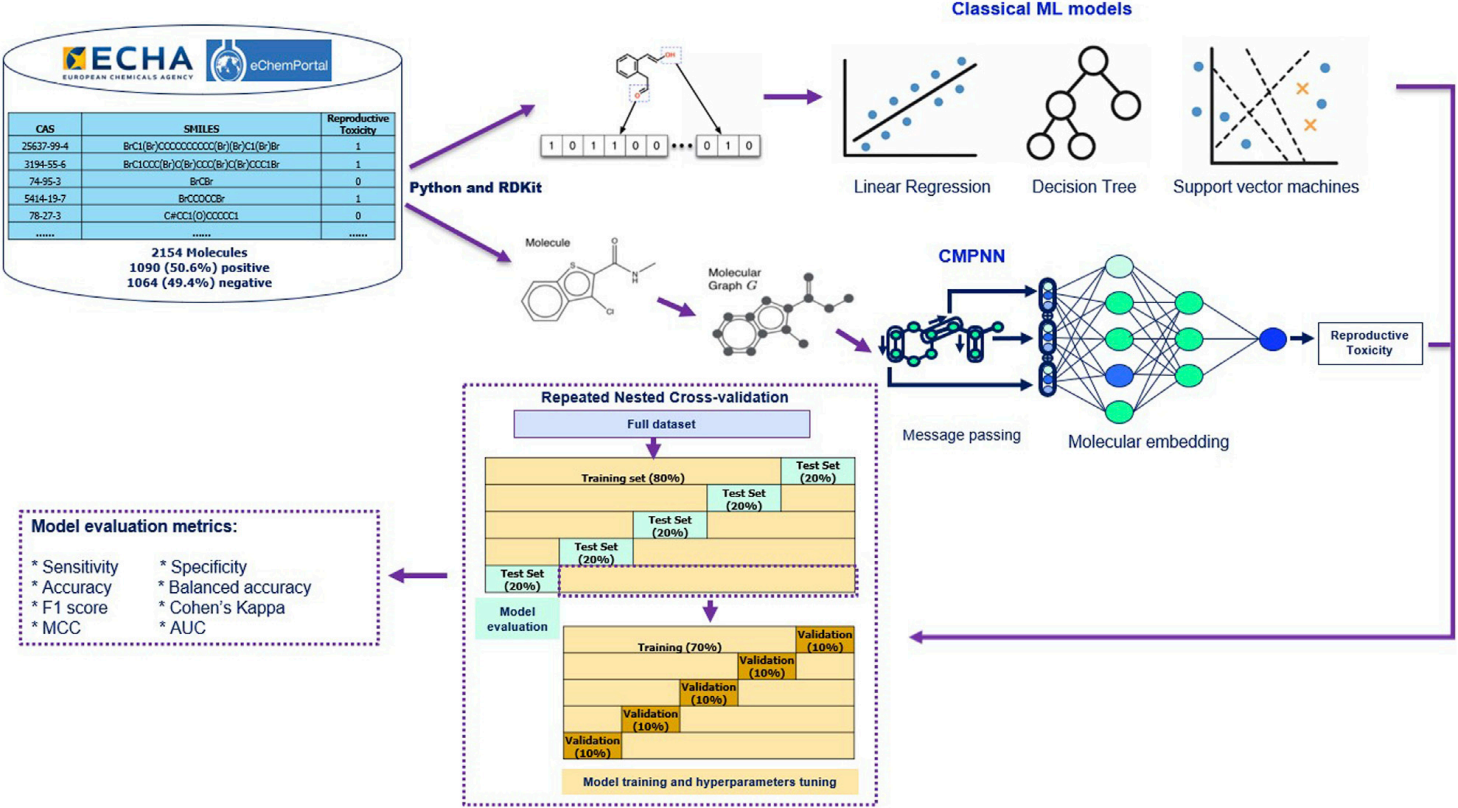
Machine learning and deep learning process to predict reproductive toxicity.

Using Python and RDKit, we generated classical molecular fingerprints and physicochemical descriptors as input features for various machine learning models like Random Forest (RF) and Support Vector Machines (SVM). To overcome the limitations of fingerprints, such as difficulty with activity cliffs and neglecting 3D conformational details, we constructed molecular graphs G and applied ReproTox-CMPNN to learn end-to-end embeddings based on automatic extraction of rich topological and chemical context from the molecular structure (Figure 2).

Model training and evaluation consisted of a rigorous repeated nested cross-validation scheme. In the outer loop, the full dataset was randomly partitioned into five distinct folds, with one fold each time serving as the test set for performance evaluation. In the inner loop, a similar procedure was also repeated five times, with 12.5% each time (10% of the total data) serving for validation/hyperparameter tuning. Nested cross-validation is more robust than 1-layer five-fold cross-validation as it uses inner loop for tuning and a separate set in outer loop for unbiased testing. The 80/20 split in the outer loop is a widely adopted guideline that balances the need for ample training data to learn patterns while retaining a sufficiently large test set to assess generalization; Gholamy et al. (2018) concluded that “p ≈ 80% is empirically the best division into the training and the testing sets.” This system ensured our results’ robustness and reproducibility, laying a solid foundation for future virtual screening and toxicological risk assessment.

Binary cross-entropy loss was minimized using the Adam optimizer, batch size = 50, maximum epochs = 60, with early stopping patience = 10 on validation AUC. Hyperparameters (hidden dimension = 256) were tuned via grid search within each training fold. All experiments were implemented in PyTorch and run on a Linux server equipped with 6 NVIDIA 4090 GPUs.

### 2.6 Model evaluation metrics

The comparison of machine learning and ReproTox-CMPNN models was based on accuracy score (ACC), balanced accuracy (BA), Cohen’s Kappa, Matthews correlation coefficient (MCC), F1 score, and the area under the curve (AUC) of the receiver operating characteristic curve (ROC). The designations TP, TN, FP, and FN in calculations refer to the number of true positives, true negatives, false positives, and false negatives, respectively. Compared to accuracy, Cohen’s kappa accounts for the possibility of agreements due to randomness, with $p_{o}$ defined as the observed agreement between two classifiers and $p_{e}$ as the expected agreement by chance.

$$Sensitivity=True\;positive\;rate\;\left(TPR\right)=\frac{TP}{TP+FN}$$

$$Specificity=1-False\;positive\;rate\;\left(FPR\right)=1-\frac{FP}{TN+FP}=\frac{TN}{TN+FP}$$

$$Accuracy=\frac{TP+TN}{TP+TN+FP+FN}$$

$$Balanced\;Accuracy=0.5*\;\left(sensitivity+specificity\right)$$

$$F1\;score=2*\left(\frac{\frac{TP}{TP+FP}*\frac{TP}{TP+FN}}{\frac{TP}{TP+FP}+\frac{TP}{TP+FN}}\right)$$

$$Cohen^{\prime }s\;Kappa=\frac{p_{o}-p_{e}}{1-p_{e}}\;where$$

$$p_{o}=\frac{TP+TN}{TP+TN+FP+FN}\;and\;{\;p}_{e}=\frac{\left(TP+FP\right)\left(TP+FN\right)+\left(FN+TN\right)\left(FP+TN\right)}{{\left(TP+TN+FP+FN\right)}^{2}}$$

$$MCC=\frac{\left(TP*TN\right)-\left(FP*FN\right)}{\sqrt{\left(TP+FP\right)\left(TP+FN\right)\left(TN+FP\right)\left(TN+FN\right)}}$$

AUC is calculated as the following with FPR as X-axis and TPR as Y-axis. A higher AUC value indicates a greater ability of the model to distinguish between positive and negative cases, i.e., better performance:

$$AUC=\int_{0}^{1}TPR\left(t\right)*dFPR\left(t\right)$$

or using trapezoidal rule,

$$AUC\approx \sum_{i=1}^{n-1}\left({FPR}_{i+1}-{FPR}_{i}\right)*\frac{{TPR}_{i+1}+{\;TPR}_{i}}{2}$$

Similarly, for other metrics, a value closer to 1 indicates superior model performance.

## 3 Results

### 3.1 Key molecular descriptors

The dataset used in this study contains Simplified Molecular Input Line Entry Specifications (SMILES) and a binary classification of reproductive toxicity (1 = Yes, 0 = No). Of the 2,154 chemicals, mainly organic compounds, 1,091 (51%) and 1,063 (49%) are classified as reproductively toxic and non-toxic, respectively. This balanced distribution provides a solid foundation for subsequent QSAR model training and validation.

**FIGURE 3:**
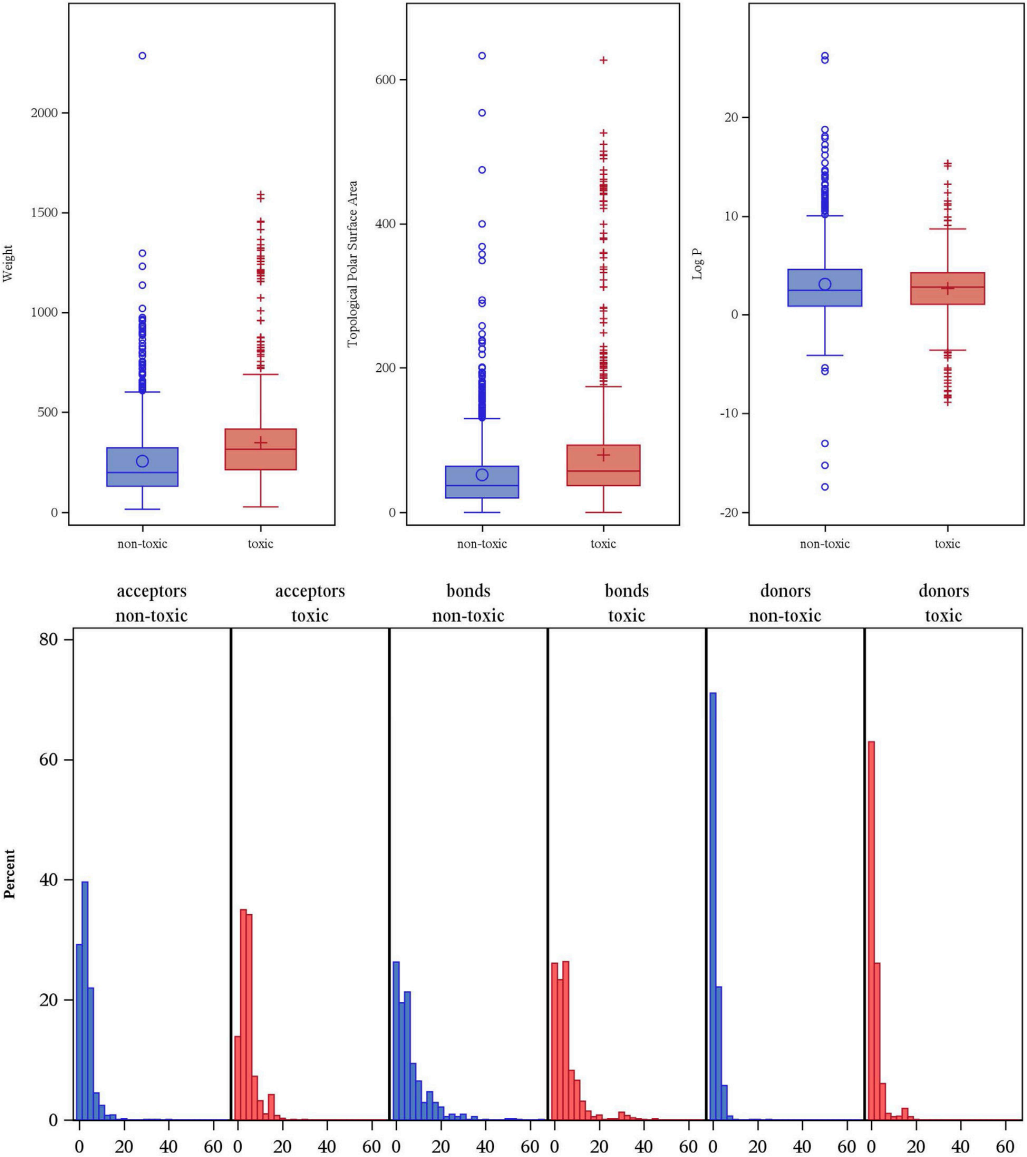
Distribution of key molecular descriptors by reproductive toxicity classification.

To better understand the physicochemical features that distinguish reproductive toxicants from non-toxic compounds, we first assessed six common molecular descriptors across our dataset. These six descriptors reflect basic physicochemical characteristics of molecules as well as widely used in QSAR models and drug discovery. As shown in Figure 3, we compared them between toxic and non-toxic compounds. Overall, toxicants exhibit higher median values of and larger dispersion in molecular weight (Weight) and topological polar surface area (TPSA) compared to non-toxic molecules, suggesting that larger and more polar structures are more likely reproductive toxicants. Specifically, the median molecular weight in the non-toxic group is 200 Da compared to 314 Da in the toxic group; similarly, the median non-toxic TPSA is 37 Å^2^ compared to 58 Å^2^ for toxicants with a more right-skewed distribution with more extreme outliers exceeding 200 Å^2^. In terms of lipophilicity, the median of logarithm of the partition coefficient (Log P) is 2.43 in the non-toxic class, compared to 2.76 in the toxic class, although the non-toxic class displays more outliers beyond a very high value (>10), reflecting that enhanced lipophilicity may facilitate membrane permeation and toxic bioaccumulation.

For hydrogen bond donors and acceptors, a slightly more right-skewed distribution in the toxic class indicates potential involvement of hydrogen-bonding interactions in mediating reproductive toxicity. However, comparable distributions between the two classes suggest that these descriptors alone are insufficient for clear-cut classification. Similarly, distributions of rotatable bonds imply limited impact of molecular flexibility on toxicity risk. Taken together, while several molecular descriptors show discernible trends between toxic and non-toxic compounds, these distributions’ overall comparability indicates a need for multivariate modeling. Integrating these descriptors within a comprehensive machine learning framework would be key to robust and generalizable reproductive toxicity predictions.

### 3.2 Hyperparameter search and model training

ReproTox-CMPNN uses a hyperparameter configuration carefully designed to improve predictive performance by balancing convergence and efficiency (Table 1). We used 60 training epochs with a batch size of 50, random enough to avoid local optima while effectively taking advantage of GPU memory to handle molecules of different sizes. We additionally employed an adaptive learning rate through a two-epoch warm-up phase followed by a decay phase.

**TABLE 1:** Hyperparameters of ReproTox-CMPNN.

Hyperparameter	Value	Description
epochs	60	Number of epochs to run
batch_size	50	Batch size
warmup_epochs	2	Epochs for linear LR warmup
init_lr	1.00E-04	Initial learning rate
max_lr	1.00E-03	Maximum learning rate
final_lr	1.00E-04	Final learning rate
hidden_size	300	Dimensionality of hidden layers in MPN
bias	FALSE	Whether to add bias to linear layers
depth	3	Number of message passing steps
activation	ReLU	Activation function
undirected	FALSE	Use undirected edges
ffn_hidden_size	None	Hidden dim for FFN
ffn_num_layers	2	Number of layers in FFN
atom_messages	FALSE	Use atom-to-atom messages
ensemble_size	1	Number of models in the ensemble
num_folds	5	Number of folds in cross-validation
split_sizes	0.7:0.1:0.2	Dataset split ratio

Learning rate increases linearly from 1e-4 to 1e-3 in the warm-up stage and then decreases exponentially to 1e-4. This method reduces instability during early training, accelerates convergence in the middle stage, and allows for fine-tuning in later stages.

In ReproTox-CMPNN’s architecture, we set the hidden layer dimension to 300 to sufficiently capture complex structural information in molecular graphs while avoiding over-parameterization; in molecular graph neural networks, this hidden layer dimension determines the richness of node (atom) and edge (bond) representations, an important part of the model. ReproTox-CMPNN’s linear layers default to not using bias terms, reducing the number of model parameters and lowering overfitting risk. For molecular graph representations, relative relationships are typically more important than absolute offsets, and this bias-free design enables the model to focus more on learning the relative feature importance in molecular structures. The message passing depth is set to 3, allowing each atom to perceive neighboring atoms up to three hops away. For most drug molecules, this depth adequately captures key structural information and chemical environments while avoiding over-smoothing and overfitting problems that deeper networks might introduce.

**FIGURE 4:**
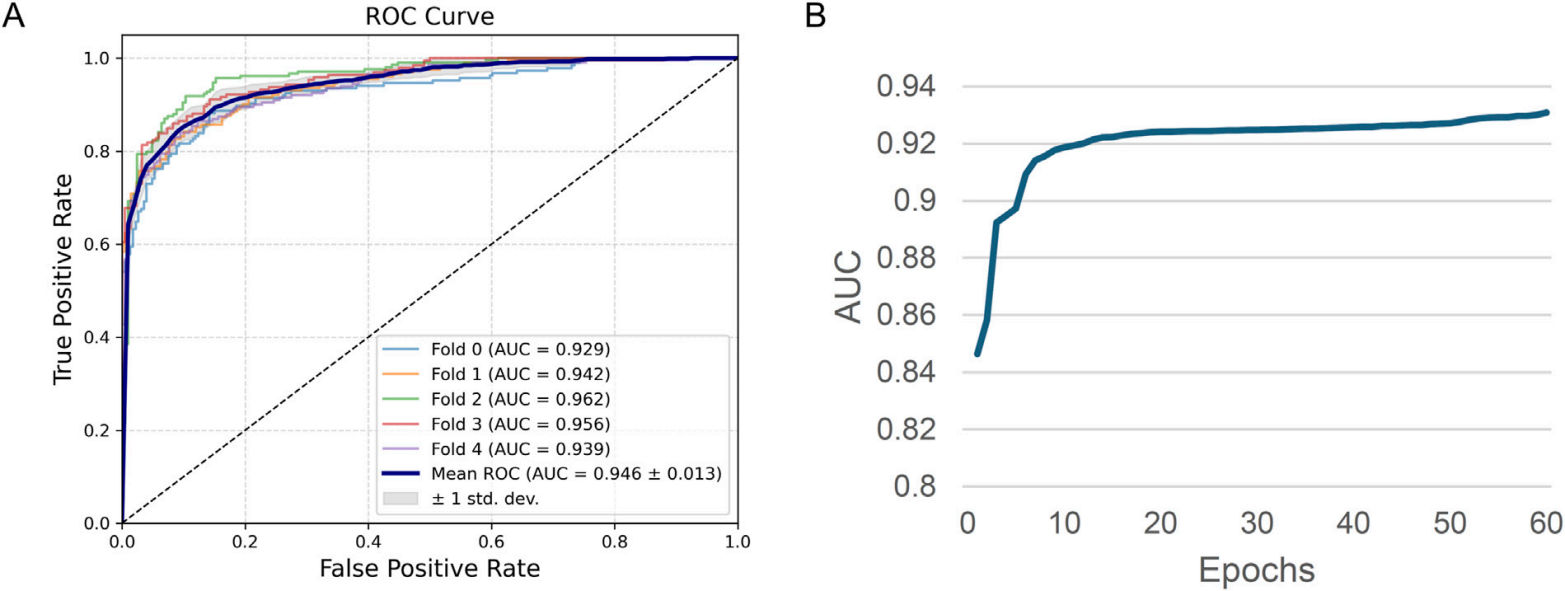
**(A)** ROC Curve, **(B)** AUC change during model training.

We obtained results presented in Figure 4A which show the ROC curves of the ReproTox-CMPNN model under five-fold cross-validation. The AUC values for folds 0–4 are 0.929, 0.942, 0.962, 0.956, and 0.939, respectively, resulting in a mean AUC of 0.946 with a standard deviation of 0.013. These results demonstrate excellent discriminative power and low variability across different folds, indicating robust stability and generalization performance. Figure 4B, showing the evolution of AUC during training epochs, illustrates the model’s rapid improvement within the first 10 epochs, with AUC rising from approximately 0.85 to over 0.92, after which the curve gradually flattens and stabilizes around 0.93. This behavior suggests fast convergence and no significant overfitting during later stages of training coupled with a consistent predictive accuracy throughout.

Overall, the CMPNN-based model achieves high and consistent AUC scores in cross-validation and demonstrates quick convergence and resilience during the training process, thus providing a solid foundation for reliable reproductive toxicity prediction of new compounds.

## 4 Discussion

### 4.1 Comparison of model performance

To provide a comprehensive benchmark of predictive methods on our reproductive toxicity dataset, we first evaluated eleven classical machine learning algorithms alongside the ReproTox-CMPNN model. As summarized in Table 2, ReproTox-CMPNN achieved an AUC of 0.946, substantially outperforming the next-best methods Random Forest (0.825) and Linear SVM (0.823). This clear margin underscores ReproTox-CMPNN’s superior ability to capture intricate molecular topology and chemical context, leading to markedly improved discrimination between toxic and non-toxic compounds.

**TABLE 2:** Model performance based on machine learning and the ReproTox-CMPNN algorithm.

Model	AUC mean (std*)	ACC mean (std)	BA mean (std)	Sensitivity mean (std)	Specificity mean (std)	Kappa mean (std)	MCC mean (std)	F1 score mean (std)
Decision Tree	0.730 (0.018)	0.725 (0.020)	0.730 (0.018)	0.683 (0.036)	0.778 (0.016)	0.454 (0.039)	0.459 (0.037)	0.7632 (0.019)
Nearest Neighbors	0.703 (0.008)	0.684 (0.015)	0.703 (0.008)	0.516 (0.018)	0.889 (0.003)	0.388 (0.022)	0.428 (0.016)	0.642 (0.012)
Linear SVM	0.825 (0.001)	0.813 (0.003)	0.825 (0.001)	0.710 (0.005)	0.939 (0.07)	0.633 (0.004)	0.655 (0.001)	0.807 (0.001)
Naive Bayes	0.775 (0.005)	0.767 (0.007)	0.775 (0.005)	0.692 (<0.001)	0.858 (0.009)	0.538 (0.013)	0.550 (0.013)	0.765 (<0.001)
Logistic Regression	0.797 (0.021)	0.792 (0.019)	0.797 (0.021)	0.751 (0.008)	0.843 (0.034)	0.586 (0.038)	0.591 (0.040)	0.799 (0.021)
RandomForest	0.813 (0.007)	0.808 (0.009)	0.813 (0.007)	0.765 (0.015)	0.861 (0.005)	0.617 (0.018)	0.623 (0.016)	0.814 (0.006)
AdaBoost	0.773 (0.007)	0.766 (0.004)	0.773 (0.007)	0.706 (0.012)	0.841 (0.027)	0.536 (0.010)	0.546 (0.014)	0.768 (0.006)
GradientBoosting	0.811 (0.004)	0.804 (0.003)	0.811 (0.004)	0.748 (0.002)	0.874 (0.009)	0.611 (0.006)	0.619 (0.008)	0.807 (0.006)
Extra Trees	0.809 (0.006)	0.804 (0.005)	0.809 (0.006)	0.763 (0.019)	0.855 (0.022)	0.610 (0.010)	0.616 (0.011)	0.811 (0.005)
Lightboost	0.828 (0.014)	0.821 (0.010)	0.828 (0.014)	0.768 (0.012)	0.888 (0.040)	0.645 (0.022)	0.654 (0.027)	0.825 (0.011)
XGBoost	0.825 (0.009)	0.820 (0.007)	0.825 (0.009)	0.778 (0.003)	0.873 (0.015)	0.642 (0.015)	0.647 (0.016)	0.826 (0.011)
ReproTox-CMPNN	0.946 (0.013)	0.857 (0.019)	0.856 (0.018)	0.823 (0.076)	0.890 (0.085)	0.713 (0.037)	0.721 (0.033)	0.846 (0.018)

*standard deviation.

In terms of overall classification metrics, ReproTox-CMPNN attained a mean accuracy of 0.857 with a standard deviation of 0.019 and a balanced accuracy (BA) of 0.856 with a standard deviation of 0.018, significantly higher than those of other models (e.g., Random Forest 0.825).

These results indicate that ReproTox-CMPNN delivers not only excellent overall predictive power but also maintains high balance between positive (toxic) and negative (non-toxic) classes, effectively mitigating the biases arising from class imbalance. Furthermore, its sensitivity (mean 0.823 and standard deviation 0.076) and specificity (mean 0.890 and standard deviation 0.085) both exceed 0.80, demonstrating reliable recall of toxicants and exclusion of non-toxicants.

Examining more stringent agreement measures, ReproTox-CMPNN’s Cohen’s Kappa (mean 0.713 and standard deviation 0.037) and Matthews Correlation Coefficient (mean 0.721 and standard deviation 0.033) are well above those of traditional models (most of which fall between 0.50 and 0.65), highlighting strong consistency and correlation with true labels. The F1 score of mean of 0.846 and standard deviation of 0.018, further reflects a balanced trade-off between precision and recall, particularly excelling in identifying toxic compounds. In contrast, simpler approaches such as K-Nearest Neighbors (AUC = 0.717) or Naive Bayes (AUC = 0.783) exhibit inferior discrimination and stability.

In summary, the ReproTox-CMPNN model, through its advanced representation of molecular structures, significantly surpasses various conventional machine learning algorithms, offering a powerful and robust framework for reproductive toxicity prediction with promising applicability in risk assessment pipelines.

### 4.2 Comparison with recent prediction models on reproductive toxicity

As shown in Table 3, in recent work regarding reproductive toxicity prediction, Jiang et al. (2019) used a much more extensive dataset (1,823 chemicals) compared to previous publications, multiple endpoints such as sperm reduction and infertility, and six machine learning methods to develop more reliable models. Their study recommended the SVM model using Molecular Access System Keys Fingerprints (MACCSFP), which generated an AUC of 0.900 and an accuracy score of 0.836. As previous work mainly focused on frequentist methods, Zhang et al. (2020) investigated Naive Bayes (NB) model together with six molecular descriptors and ten types of fingerprints. Their best model resulted in an AUC of 0.888 and an accuracy score of 0.830. Feng et al. (2021) used three machine learning methods with nine molecular fingerprints to build more ensemble models. The model they recommended had an AUC of 0.920 and an accuracy score of 0.844. Ren et al. (2024) developed a deep learning fragment-based graph transformer network (FGTN) model to predict reproductive toxicity, taking pre-generated fragments as nodes and bonds between fragments as edges with a super-molecule-level node to connect all fragment nodes. The FGTN model showed an AUC of 0.914 and an accuracy score of 0.861.

**TABLE 3:** Comparison to the previous models reported in the literature.

Model name	AUC	ACC	MCC	No. of compounds	References
MACCSFP-SVM^a^	0.900	0.836	0.679	1823	Jiang et al. (2019)
NB-1^b^	0.888	0.830	0.663	1685	Zhang et al. (2020)
Ensemble-Top12^c^	0.920	0.844	-^d^	1823	Feng et al. (2021)
FGTN^e^	0.914	0.861	0.723	2053	Ren et al. (2024)
ReproTox-CMPNN^f^	0.946	0.857	0.721	2154	Current study

^a^ MACCSFP-SVM, support vector machine model based on MACCS, fingerprints.

^b^ NB-1, naïve bayes-classifier model based on six molecular descriptors and LCFC_20 fingerprints.

^c^ Ensemble-Top12, ensemble model based on support vector machine, random forest, and extreme gradient boosting methods and 9 molecular fingerprints.

^d^ Not reported.

^e^ Fragment-based graph transformer network.

^f^ results based on a repeated nested cross-validation procedure.

Due to differences in datasets and data splitting, a direct comparison between the aforementioned models and the ReproTox-CMPNN model is not possible. However, a simple comparison can still provide some valuable information. ReproTox-CMPNN remarkably outperformed other models in AUC and was very close to the FGTN model in ACC and MCC, exceeding the remaining models. These results suggest that the ReproTox-CMPNN model is both reliable and robust (Table 3).

CMPNN’s strong performance across multiple molecular property benchmarks is attributed to its enhanced messaging framework: unlike traditional MPNNs that focus solely on node-to-node communication, CMPNN utilizes a communicative kernel to reinforce interactions between both node and edge features, complemented by a message booster, generating richer molecular representations. As it is inherently based on 2D molecular graphs, CMPNN may miss stereochemistry and spatial relationships crucial for predicting properties like enantiomer-specific activity. Furthermore, because our training set mainly comprises organic environmental pollutants, our model’s generalizability to other chemical domains such as metal complexes or inorganic salts remains to be demonstrated. Finally, since our current evaluation focuses on specific endpoints, validating CMPNN across a broader spectrum of toxicity readouts such as DNA damage, endocrine disruption will be needed to further illuminate its applicability.

### 4.3 Conclusion and future work

In this study, we built prediction models on a comprehensive reproductive toxicity dataset with 2,154 chemicals and compared the performance of the deep learning ReproTox-CMPNN model with other methods. Our results show that ReproTox-CMPNN learning results exceed the current best ML models in both embedding quality and predictive accuracy, making it a new state-of-the-art method in this field. ReproTox-CMPNN’s deep capture of multi-level molecular relationships offers an efficient and reliable computational tool for rapid chemical safety screening and risk assessment.

Future work includes training ReproTox-CMPNN to predict multiple toxicity endpoints or reproductive toxicity of chemical mixtures as well as combining ReproTox-CMPNN with mask language model (MLM) for protein embedding to further improve predictions.

## Data Availability

The original contributions presented in the study are included in the article/Supplementary Material, further inquiries can be directed to the corresponding authors.
