# Supplementary material for: A deep-learning approach to predict reproductive toxicity of chemicals using communicative message passing neural network
Source: Front Toxicol. 2025 Jul 22;7:1640612. doi: 10.3389/ftox.2025.1640612 (PMC12321834; doi:10.3389/ftox.2025.1640612)
Supplement: Supplementary file 1 [file Supplementaryfile1.docx]

# Supplementary Materials

A Deep-Learning Approach to Predict Reproductive Toxicity of Chemicals Using Communicative Message Passing Neural Network

**Owen He1*, Daoxing Chen2, Yimei Li3***

1Deerfield Academy, Deerfield, MA, United States

2School of Pharmaceutical Sciences, Wenzhou Medical University, Wenzhou, China

3Department of Biostatistics, St. Jude Children’s Research Hospital, Memphis, TN, United States

*** Correspondence:**Owen He

[ohe26@deerfield.edu](mailto:ohe26@deerfield.edu)

Yimei Li

[yimei.li@stjude.org](mailto:yimei.li@stjude.org)

# CONTENTS

## Tables

**Table S1. Reproductive toxicity dataset.**

**Table S2. The algorithm implementation and key hyperparameters of machine learming models.**

**Table S1. Reproductive toxicity dataset.**

| **SMILES** | **Reproductive Toxcicity**  **(1=Yes, 0=No)** |
| --- | --- |
| ClC(=O)Cl | 1 |
| [C-]#[O+] | 1 |
| CC(=O)O | 1 |
| ClCC(Br)CBr | 1 |
| C1C=CC2C3CC(C=C3)C12 | 1 |
| CC(C)Br | 1 |
| ClCCl | 1 |
| CCO | 1 |
| CCOCCOCC | 1 |
| Cc1ccccc1 | 1 |
| CC(=O)CC(C1=C(O)Oc2ccccc2C1=O)c3ccccc3 | 1 |
| CN1CCCC1=O | 1 |
| CC(=C)C(=O)OCC1CO1 | 1 |
| OC1=C(C2CC(Cc3ccccc23)c4ccc(cc4)c5ccc(Br)cc5)C(=O)Oc6ccccc16 | 1 |
| COC(C)COC(=O)C | 1 |
| CCCCCOC(=O)c1ccccc1C(=O)OCCCCC | 1 |
| Oc1c(cc(c(O)c1[N+](=O)[O-])[N+](=O)[O-])[N+](=O)[O-] | 1 |
| CCCCOC(=O)c1ccccc1C(=O)OCc2ccccc2 | 1 |
| CCCCC(CC)COC(=O)c1ccccc1C(=O)OCC(CC)CCCC | 1 |
| CC(C)(C)c1ccc(cc1)C(=O)O | 1 |
| CC(C)(C)C1=NN(C(=O)O1)c2cc(OCC#C)c(Cl)cc2Cl | 1 |
| NCCN | 1 |
| Nc1nc[nH]n1 | 1 |
| CC(=O)N1CCN(CC1)c2ccc(OCC3COC(Cn4ccnc4)(O3)c5ccc(Cl)cc5Cl)cc2 | 1 |
| CC1CC(C)C(=O)C(C1)C(O)CC2CC(=O)NC(=O)C2 | 1 |
| CN(C)C(=O)C | 1 |
| CN(C)C=O | 1 |
| c1c[nH]cn1 | 1 |
| CCN1CCCC1=O | 1 |
| CCC | 1 |
| CCCC | 1 |
| CCCCC | 1 |
| CCC(CO)NCCNC(CC)CO | 1 |
| CC1(C)OC2CC3C4CC(F)C5=CC(=O)C=CC5(C)C4C(O)CC3(C)C2(O1)C(=O)CO | 1 |
| FC(F)OC(F)(F)C(F)Cl | 1 |
| CC1C2C(O)C3C(N(C)C)C(=O)C(=C(O)C3(O)C(=C2C(=O)c4c(O)cccc14)O)C(=O)N | 1 |
| CC(=O)OC1CCC2(C)C3CCC4(C)C(CC=C4C(=O)C)C3CC=C2C1 | 1 |
| CC1CC2C3CC(F)C4=CC(=O)C=CC4(C)C3C(O)CC2(C)C1C(=O)COC(=O)C(C)(C)C | 1 |
| CCCCc1oc2ccccc2c1C(=O)c3cc(I)c(OCCN(CC)CC)c(I)c3 | 1 |
| C=CC(=O)OCC(COCC(COC(=O)C=C)(COC(=O)C=C)COC(=O)C=C)(COC(=O)C=C)COC(=O)C=C | 1 |
| OCC1CO1 | 1 |
| CCCCCCCCCCCC(=O)O | 1 |
| CCCCC(=O)OC1CCC2C3CCc4cc(O)ccc4C3CCC12C | 1 |
| CCCCCC(=O)OC1(CCC2C3CCC4=CC(=O)CCC4C3CCC12C)C(=O)C | 1 |
| CCCCCC(O)C=CC1C(O)CC(=O)C1CC=CCCCC(=O)O | 1 |
| COc1ccc2[nH]cc(CCNC(=O)C)c2c1 | 1 |
| COc1cccc2C(=O)c3c(O)c4CC(O)(CC(OC5CC(N)C(O)C(C)O5)c4c(O)c3C(=O)c12)C(=O)CO | 1 |
| CCCCCC(=O)OCC(=O)C1(O)CCC2C3CCC4=CC(=O)CCC4(C)C3C(O)CC12C | 1 |
| OC(=O)CCCC1CCCCC1 | 1 |
| NCC1OC(OC2C(N)CC(N)C(OC3OC(CO)C(O)C(N)C3O)C2O)C(O)C(O)C1O | 1 |
| ClC(Cl)C(c1ccc(Cl)cc1)c2ccccc2Cl | 1 |
| OS(=O)O | 1 |
| CN(C)C1C2CC3Cc4c(ccc(O)c4C(=C3C(=O)C2(O)C(=C(C(=O)N)C1=O)O)O)N(C)C | 1 |
| COC(=O)C1C(CC2CCC1N2C)OC(=O)c3ccccc3 | 1 |
| CC12CCC(=O)C=C1CCC3C2CCC4(C)C3CCC4(O)C#C | 1 |
| CC12CC(O)C3C(CCC4=CC(=O)C=CC34C)C1CCC2(O)C(=O)COP(=O)(O)O | 1 |
| CC12CCC3C(CCc4cc(O)ccc34)C1CCC2=O | 1 |
| CCCCCCC(=O)OCCN1CCN(CCCN2c3ccccc3Sc4ccc(cc24)C(F)(F)F)CC1 | 1 |
| CCCCCCCCCC(=O)OCCN1CCN(CCCN2c3ccccc3Sc4ccc(cc24)C(F)(F)F)CC1 | 1 |
| CC(=O)OC1CCC2C3CCC4CC(=O)C=C(C)C4(C)C3CCC12C | 1 |
| CCOC(=O)C(CCc1ccccc1)NC(C)C(=O)N2CCCC2C(=O)O | 1 |
| CC12CCC3C(CCC4=CC(=O)C=CC34C)C1CCC2O | 1 |
| Cc1nnc2CN=C(c3ccccc3)c4cc(Cl)ccc4n12 | 1 |
| CCC(=C(CC)c1ccc(O)cc1)c2ccc(O)cc2 | 1 |
| CC12CCC3C(CCc4cc(OC(=O)c5ccccc5)ccc34)C1CCC2O | 1 |
| CC12CC(=O)C3C(CCC4=CC(=O)C=CC34C)C1CCC2(O)C(=O)CO | 1 |
| CC(=O)OC1(CCC2C3CCC4=CC(=O)CCC4(C)C3CCC12C)C(=O)C | 1 |
| CCCCN(CCCC)CCCOc1ccc(cc1)C(=O)c2c(CCCC)oc3ccc(NS(=O)(=O)C)cc23 | 1 |
| CC(=O)OC1(CCC2C3C=C(Cl)C4=CC(=O)CCC4(C)C3CCC12C)C(=O)C | 1 |
| CC12CCC3C(CCC4=CC(=O)CCC34C)C1CCC2=O | 1 |
| COC1C(CC2CN3CCc4c([nH]c5cc(OC)ccc45)C3CC2C1C(=O)OC)OC(=O)c6cc(OC)c(OC)c(OC)c6 | 1 |
| FC(F)OC(Cl)C(F)(F)F | 1 |
| Cc1cccc(C)c1O | 1 |
| FC1=CN(C2CCCO2)C(=O)NC1=O | 1 |
| CC12Cc3cnoc3C=C1CCC4C2CCC5(C)C4CCC5(O)C#C | 1 |
| CCCCCC(=O)OCC(=O)C1(O)CCC2C3CCC4=CC(=O)C=CC4(C)C3C(O)CC12C | 1 |
| CCC(c1ccccc1)C(O)(c2ccccc2)c3ccc(OCCN(C)C)cc3 | 1 |
| CCCC(CCC)C(=O)O | 1 |
| CC(=O)OCC(=O)C1CCC2C3CCC4=CC(=O)CCC4(C)C3CCC12C | 1 |
| CCCCCCCCCCCCCCCCCC(=O)O | 1 |
| OC(=O)CN(CCN(CC(=O)O)CC(=O)O)CCN(CC(=O)O)CC(=O)O | 1 |
| OC(=O)c1ccc(O)cc1O | 1 |
| CC(C)(S)C(N)C(=O)O | 1 |
| CC12CC(=C(O)C=C1CCC3C2CCC4(C)C3CCC4(O)C#C)C=O | 1 |
| CCCSc1ccc2[nH]c(NC(=O)OC)nc2c1 | 1 |
| CC1(O)CCC2C3CCC4=CC(=O)C=CC4(C)C3CCC12C | 1 |
| Cc1ccncn1 | 1 |
| CCC(=C(c1ccccc1)c2ccc(OCCN(C)C)cc2)c3ccccc3 | 1 |
| CC1CC2C3CCC4=CC(=O)C=CC4(C)C3(F)C(O)CC2(C)C1(O)C(=O)COP(=O)(O)O | 1 |
| NC12CC3CC(CC(C3)C1)C2 | 1 |
| CN1CCN(Cc2ccc(cc2)C(=O)Nc3ccc(C)c(Nc4nccc(n4)c5cccnc5)c3)CC1 | 1 |
| CC12CCC3(O)C(CCC4=CC(=O)CCC34C)C1CCC2=O | 1 |
| CC1=C(CC(=O)O)c2cc(F)ccc2C1=Cc3ccc(cc3)S(=O)C | 1 |
| OC(=O)CC(O)(CC(=O)O)C(=O)O | 1 |
| Fc1ccc(cc1)C2CCNCC2COc3ccc4OCOc4c3 | 1 |
| OS(=O)(=O)O | 1 |
| CC(C)NCC(O)COc1cccc2ccccc12 | 1 |
| CC1CC2C3CCC4=CC(=O)C=CC4(C)C3C(=O)CC2(C)C1(O)C(=O)CO | 1 |
| CCNC(=O)C1CCCN1C(=O)C(CCCN=C(N)N)NC(=O)C(CC(C)C)NC(=O)C(COC(C)(C)C)NC(=O)C(Cc2ccc(O)cc2)NC(=O)C(CO)NC(=O)C(Cc3c[nH]c4ccccc34)NC(=O)C(Cc5c[nH]cn5)NC(=O)C6CCC(=O)N6 | 1 |
| CC12CCC3C(CCC4=CC(=O)C=CC34C)C1CCC2=O | 1 |
| Clc1cccc(N2CCN(CCCCOc3ccc4CCC(=O)Nc4c3)CC2)c1Cl | 1 |
| O=C1CCC(N2C(=O)c3ccccc3C2=O)C(=O)N1 | 1 |
| CC(=O)OC1(CCC2C3CCC4=CC(=O)CCC4C3CCC12C)C(=O)C | 1 |
| CCOC(O)C(F)(F)F | 1 |
| CCC12CCC3C(CCC4=CC(=O)CCC34)C1CCC2(O)C#C | 1 |
| COCC(NC(=O)C)C(=O)NCc1ccccc1 | 1 |
| CCN(CC)C(=O)C1CN(C)C2Cc3c[nH]c4cccc(C2=C1)c34 | 1 |
| OC(=O)c1cc(ccc1O)N=Nc2ccc(cc2)S(=O)(=O)Nc3ccccn3 | 1 |
| CC1(O)CCC2C3CCC4CC(O)CCC4(C)C3CCC12C | 1 |
| CCCCC(=O)N(Cc1ccc(cc1)c2ccccc2c3nn[nH]n3)C(C(C)C)C(=O)O | 1 |
| CC(=CC=CC(=CC(=O)O)C)C=CC1=C(C)CCCC1(C)C | 1 |
| CCCCCOC(=O)NC1=NC(=O)N(C=C1F)C2OC(C)C(O)C2O | 1 |
| CC(=O)CC(=O)C | 1 |
| CC(C)CC(NC(=O)CNC(=O)C(Cc1ccc(O)cc1)NC(=O)C(CO)NC(=O)C(Cc2c[nH]c3ccccc23)NC(=O)C(Cc4c[nH]cn4)NC(=O)C5CCC(=O)N5)C(=O)NC(CCCN=C(N)N)C(=O)N6CCCC6C(=O)NCC(=O)N | 1 |
| CN1C(=C(O)c2ccccc2S1(=O)=O)C(=O)Nc3ncc(C)s3 | 1 |
| CN(C)CCCC1(OCc2cc(ccc12)C#N)c3ccc(F)cc3 | 1 |
| ClCCN(CCCl)P1(=O)NCCCO1 | 1 |
| CN=C(NCCSCc1[nH]cnc1C)NC#N | 1 |
| CCCCCCC(=O)OCC(=O)C1CCC2C3CCC4=CC(=O)CCC4(C)C3CCC12C | 1 |
| Nc1nc(S)c2[nH]cnc2n1 | 1 |
| CCCCCCC(=O)OC1CCC2C3CCC4CC(=O)C=C(C)C4(C)C3CCC12C | 1 |
| CNNCc1ccc(cc1)C(=O)NC(C)C | 1 |
| CCN(CC)C(=S)S | 1 |
| CCC1(CCC(=O)NC1=O)c2ccc(N)cc2 | 1 |
| OC(=O)O | 1 |
| OC(C(O)C(=O)O)C(=O)O | 1 |
| CCNC1CC(N)C(OC2OC(=CCC2N)CN)C(O)C1OC3OCC(C)(O)C(NC)C3O | 1 |
| CC1=C(CCN2CCC(CC2)c3noc4cc(F)ccc34)C(=O)N5CCCCC5=N1 | 1 |
| CCN1C=C(C(=O)O)C(=O)c2cc(F)c(cc12)N3CCN(C)CC3 | 1 |
| COC(C1Cc2cc3cc(OC4CC(OC5CC(O)C(OC)C(C)O5)C(OC(=O)C)C(C)O4)c(C)c(O)c3c(O)c2C(=O)C1OC6CC(OC7CC(OC8CC(C)(O)C(OC(=O)C)C(C)O8)C(O)C(C)O7)C(O)C(C)O6)C(=O)C(O)C(C)O | 1 |
| CC1CCCC(O)CCCCCc2cc(O)cc(O)c2C(=O)O1 | 1 |
| ClC(Cl)Br | 1 |
| CN1CC(C=C2C1Cc3c[nH]c4cccc2c34)C(=O)NC5(C)OC6(O)C7CCCN7C(=O)C(Cc8ccccc8)N6C5=O | 1 |
| CC(C)CC=CC(=O)NC1C(O)C(O)C(CC(O)C2OC(C(O)C2O)N3C=CC(=O)NC3=O)OC1OC4OC(CO)C(O)C(O)C4NC(=O)C | 1 |
| ClCCNP1(=O)OCCCN1CCCl | 1 |
| CCC(=O)OC1CCC2C3CCc4cc(O)ccc4C3CCC12C | 1 |
| CNC1C(O)C2OC(OC3C(N)CC(N)C(O)C3O)C(N)CC2OC1OC4OC(CO)C(N)C(O)C4O | 1 |
| CCCCCC(=O)OC1(CCC2C3CCC4=CC(=O)CCC4(C)C3CCC12C)C(=O)C | 1 |
| CCC(C)(C)C(=O)OC1CC(C)C=C2C=CC(C)C(CCC3CC(O)CC(=O)O3)C12 | 1 |
| CC(C)NCC(O)COc1ccc(CC(=O)N)cc1 | 1 |
| CCCC(CCC)C(=O)N | 1 |
| CC12CCC3C(CCc4cc(O)ccc34)C1CC(O)C2O | 1 |
| CN(Cc1cnc2nc(N)nc(N)c2n1)c3ccc(cc3)C(=O)NC(CCC(=O)O)C(=O)O | 1 |
| CC12CCC3C(CCc4cc(O)ccc34)C1CCC2O | 1 |
| CC12CCC(=O)C=C1CCC3C4CCC(O)(C(=O)CO)C4(C)CC(O)C23 | 1 |
| CC12CC(O)C3C(CCC4=CC(=O)C=CC34C)C1CCC2(O)C(=O)CO | 1 |
| CCCCC(=O)OCC(=O)C1C(C)CC2C3CC(F)C4=CC(=O)C=CC4(C)C3(F)C(O)CC12C | 1 |
| CC1CC(=O)CC2CCC3C4CCC(OC(=O)C)C4(C)CCC3C12C | 1 |
| COc1ccc2c(c1)c(CC(=O)O)c(C)n2C(=O)c3ccc(Cl)cc3 | 1 |
| OCCO | 1 |
| OCC1OC(CC1O)N2C=C(Br)C(=O)NC2=O | 1 |
| CC1CC2C3CC(F)C4=CC(=O)C=CC4(C)C3C(O)CC2(C)C1C(=O)CO | 1 |
| CCC(=O)OC1(C(C)CC2C3CCC4=CC(=O)C=CC4(C)C3(F)C(O)CC12C)C(=O)CCl | 1 |
| CC1(C)OC2CC3C4CC(F)C5=CC(=O)C=CC5(C)C46OC6CC3(C)C2(O1)C(=O)CO | 1 |
| CC(=O)OCC(=O)C12N=C(C)OC1CC3C4CCC5=CC(=O)C=CC5(C)C4C(O)CC23C | 1 |
| CC12CCC(=O)C=C1C3CC3C4C2CCC5(C)C4C6CC6C57CCC(=O)O7 | 1 |
| CC1=CC(=O)CC2CCC3C4CCC(O)C4(C)CCC3C12C | 1 |
| CS(=O)(=O)OCCCCOS(=O)(=O)C | 1 |
| Cn1cnc(c1Sc2[nH]cnc3ncnc23)[N+](=O)[O-] | 1 |
| CC(=O)OCC(=O)C1(O)CCC2C3CCC4=CC(=O)CCC4(C)C3C(O)CC12C | 1 |
| CCC(C1=C(O)Oc2ccccc2C1=O)c3ccccc3 | 1 |
| CC12CCC3C(CCC4=CC(=O)CCC34CO)C1CCC2=O | 1 |
| Oc1c(cc(cc1[N+](=O)[O-])[N+](=O)[O-])[N+](=O)[O-] | 1 |
| CC1CC(=O)CC2CCC3C4CCC(O)C4(C)CCC3C12C | 1 |
| CC12CCC(=O)C=C1CCC3C4CCC(O)(C(=O)COC(=O)CCC(=O)O)C4(C)CC(O)C23 | 1 |
| OCl(=O)(=O)=O | 1 |
| CC1CC2C3CCC4=CC(=O)C=CC4(C)C3(F)C(O)CC2(C)C1(O)C(=O)COC(=O)C | 1 |
| CCC1(C(=O)NC(=O)NC1=O)c2ccccc2 | 1 |
| COc1c(C)c2COC(=O)c2c(O)c1CC=C(C)CCC(=O)O | 1 |
| CC(C)C(=O)Nc1ccc(c(c1)C(F)(F)F)[N+](=O)[O-] | 1 |
| COc1cc2OC3OCCC3(O)c2c4OC(=O)C5=C(CCC5=O)c14 | 1 |
| COC12C3NC3CN1C4=C(C2COC(=O)N)C(=O)C(=C(C)C4=O)N | 1 |
| COc1ccc2cc(ccc2c1)C(C)C(=O)O | 1 |
| [2H]C(=O)N(C([2H])([2H])[2H])C([2H])([2H])[2H] | 1 |
| CC12CC(O)C3C(CCC4=CC(=O)C=CC34C)C1CCC2(O)C(=O)COC(=O)CCC(=O)O | 1 |
| CCC1(C(=O)NCNC1=O)c2ccccc2 | 1 |
| O=C1NC(=O)C(N1)(c2ccccc2)c3ccccc3 | 1 |
| CC(c1c[nH]cn1)c2cccc(C)c2C | 1 |
| OCC(F)(F)F | 1 |
| CC(=O)C1CCC2C3C=CC4=CC(=O)CCC4(C)C3CCC12C | 1 |
| CC(=O)OC1CCC2C3CCC4CC(=O)CCC4(C)C3CCC12C | 1 |
| CC1CC2C(CCC3(C)C2CCC3(O)C(=O)C)C4(C)CCC(=O)C=C14 | 1 |
| CC=C1CC2CC1C=C2 | 1 |
| CC1CC2C3CCC(O)(C(=O)COC(=O)C)C3(C)CC(O)C2C4(C)C=CC(=O)C=C14 | 1 |
| CCC1NC(=O)C(C(O)C(C)CC=CC)N(C)C(=O)C(C(C)C)N(C)C(=O)C(CC(C)C)N(C)C(=O)C(CC(C)C)N(C)C(=O)C(C)NC(=O)C(C)NC(=O)C(CC(C)C)N(C)C(=O)C(NC(=O)C(CC(C)C)N(C)C(=O)CN(C)C1=O)C(C)C | 1 |
| OC(CCl)COc1cccc2ccccc12 | 1 |
| CCCC(=O)Nc1ccc(O)c(c1)C(=O)C | 1 |
| CCCCCCC(=O)OC1(CCC2C3CCC4=CC(=O)CCC4(C)C3CCC12C)C(=O)C | 1 |
| CN1C(=O)CN=C(c2ccccc2)c3cc(Cl)ccc13 | 1 |
| NCC1OC(OC2C(O)C(OC3C(O)C(N)CC(N)C3OC4OC(CN)C(O)C(O)C4N)OC2CO)C(N)C(O)C1O | 1 |
| CC12CCC3C(CCc4cc(OS(=O)(=O)O)ccc34)C1CCC2=O | 1 |
| [OH2]C(=O)CN(CCN(CC(=O)[OH2])CC(=O)O)CC(=O)O | 1 |
| CC(=O)OC1(CCC2C3C=C(C)C4=CC(=O)CCC4(C)C3CCC12C)C(=O)C | 1 |
| CC12CCC3C(CCc4cc(OC5CCCC5)ccc34)C1CCC2(O)C#C | 1 |
| CC(=O)C1CCC2C3CCC4=CC(=O)CCC4(C)C3CCC12C | 1 |
| CCCOc1ccc2C3CCC4(C)C(CCC4C3CCc2c1)OC | 1 |
| CCCC1OC2CC3C4CCC5=CC(=O)C=CC5(C)C4C(O)CC3(C)C2(O1)C(=O)CO | 1 |
| OI(=O)=O | 1 |
| CCN(CC)CCCC(C)Nc1ccnc2cc(Cl)ccc12 | 1 |
| CCC(=O)OC1CCC2C3CCC4=CC(=O)CCC4(C)C3CCC12C | 1 |
| OC(=O)CCCc1ccc(cc1)N(CCCl)CCCl | 1 |
| CC1(C)OC2CC3C4CCC5=CC(=O)C=CC5(C)C4(F)C(O)CC3(C)C2(O1)C(=O)CO | 1 |
| CN1C(=O)OC(C)(C)C1=O | 1 |
| COc1ccc(cc1)C(CN(C)C)C2(O)CCCCC2 | 1 |
| CNC1C(O)C(O)C(CO)OC1OC2C(OC3C(O)C(O)C(N=C(N)N)C(O)C3N=C(N)N)OC(C)C2(O)CO | 1 |
| CCC(=O)OCC=C(C)C=CC=C(C)C=CC1=C(C)CCCC1(C)C | 1 |
| CC(=O)OCC=C(C)C=CC=C(C)C=CC1=C(C)CCCC1(C)C | 1 |
| CNC(C)C1CCC(N)C(OC2C(N)CC(N)C(OC3OCC(C)(O)C(NC)C3O)C2O)O1 | 1 |
| Nc1nc(O)c2c(CCc3ccc(cc3)C(=O)NC(CCC(=O)O)C(=O)O)c[nH]c2n1 | 1 |
| Cc1nnc2CN=C(c3ccccc3Cl)c4cc(Cl)ccc4n12 | 1 |
| CC(=O)OC1(CCC2C3C=C(Cl)C4=CC(=O)C5CC5C4(C)C3CCC12C)C(=O)C | 1 |
| CCCCCCCCCCCCCCCC(=O)OCC=C(C)C=CC=C(C)C=CC1=C(C)CCCC1(C)C | 1 |
| OCCN1CCN(CCCN2c3ccccc3Sc4ccc(cc24)C(F)(F)F)CC1 | 1 |
| COc1cc(cc(OC)c1O)C2C3C(COC3=O)C(OC4OC5COC(C)OC5C(O)C4O)c6cc7OCOc7cc26 | 1 |
| CC1CC2C3CCC4=CC(=O)C=CC4(C)C3(F)C(O)CC2(C)C1(O)C(=O)COC(=O)c5ccncc5 | 1 |
| CC12CCC3C(CCC4=CC(=O)CCC34)C1CCC2=O | 1 |
| CCCCCCC(=O)OC1CCC2C3CCc4cc(O)ccc4C3CCC12C | 1 |
| CCCC(NC(C)C(=O)N1C2CCCCC2CC1C(=O)O)C(=O)OCC | 1 |
| CC(=O)Oc1ccccc1C(=O)O | 1 |
| CC(=O)CC(C1C(=O)Oc2ccccc2C1=O)c3ccccc3 | 1 |
| CCCCCCC(=O)OC1CCC2C3CCC4=CC(=O)CCC4(C)C3CCC12C | 1 |
| CC(C)C1NC(=O)C(NC(=O)C2=C(N)C(=O)C(=C3Oc4c(C)ccc(C(=O)NC5C(C)OC(=O)C(C(C)C)N(C)C(=O)CN(C)C(=O)C6CCCN6C(=O)C(NC5=O)C(C)C)c4N=C23)C)C(C)OC(=O)C(C(C)C)N(C)C(=O)CN(C)C(=O)C7CCCN7C1=O | 1 |
| OC(=O)c1ccccc1O | 1 |
| CC12CCC3C(CCc4cc(O)ccc34)C1CCC2(O)C#C | 1 |
| NC#N | 1 |
| CCCCCCCCCCC(=O)OC1CCC2C3CCc4cc(O)ccc4C3CCC12C | 1 |
| CN(C)C(=O)N(C)C | 1 |
| CCC(N(Cc1occc1)C(=O)n2ccnc2)C(=O)OCCCC=C | 1 |
| CCCCOC(=O)c1ccccc1C(=O)O | 1 |
| CCC(=O)OC1CCC2C3CCc4cc(OC(=O)CC)ccc4C3CCC12C | 1 |
| CC(=C)C(=O)OCCOc1ccc(cc1)C(C)(C)c2ccc(OCCOC(=O)C(=C)C)cc2 | 1 |
| NCCCCC(NC(CCc1ccccc1)C(=O)O)C(=O)N2CCCC2C(=O)O | 1 |
| OCC(O)CCl | 1 |
| CCC12CCC3C(CCC4=CC(=O)CCC34)C1C=CC2(O)C#C | 1 |
| [O-][N+](=O)N1CN(CN(CN(C1)[N+](=O)[O-])[N+](=O)[O-])[N+](=O)[O-] | 1 |
| SC#N | 1 |
| COC1CC(=O)CC(C)C12Oc3c(Cl)c(OC)cc(OC)c3C2=O | 1 |
| CC1CC2C3CCC4=CC(=O)C=CC4(C)C3(Cl)C(O)CC2(C)C1(OC(=O)c5occc5)C(=O)CCl | 1 |
| CC(=O)C1(O)CCC2C3CCC4=CC(=O)CCC4C3CCC12C | 1 |
| CC12CC(O)C3C(CCC4=CC(=O)C=CC34C)C1CC(O)C2(O)C(=O)CO | 1 |
| CCC12CC(=C)C3C(CCC4=CC(=O)CCC34)C1CCC2(O)C#C | 1 |
| NCC1(CC(=O)O)CCCCC1 | 1 |
| CC(O)(CS(=O)(=O)c1ccc(F)cc1)C(=O)Nc2ccc(C#N)c(c2)C(F)(F)F | 1 |
| Sc1[nH]cnc2ncnc12 | 1 |
| CCCc1nc(c(C(=O)OCC2=C(C)OC(=O)O2)n1Cc3ccc(cc3)c4ccccc4c5nn[nH]n5)C(C)(C)O | 1 |
| CCCCCCCCCCCCCCCCCC(=O)OCC(=O)OCC(=O)C1(O)CCC2C3CCC4=CC(=O)C=CC4(C)C3C(O)CC12C | 1 |
| CN=C1CN(O)C(=C2C=C(Cl)C=CC2=N1)c3ccccc3 | 1 |
| CCCCCC(=O)OCC(=O)C1C(C)CC2C3CC(F)C4=CC(=O)C=CC4(C)C3(Cl)C(O)CC12C | 1 |
| CC12C=CC3=C4CCC(=O)C=C4CCC3C1CCC2O | 1 |
| CC(=O)OCC(=O)C1(O)CCC2C3CCC4=CC(=O)C=CC4(C)C3C(O)CC12C | 1 |
| CC(=O)SC1CC2=CC(=O)CCC2(C)C3CCC4(C)C(CCC45CCC(=O)O5)C13 | 1 |
| CC(=O)OC1(CCC2C3CCC4=CC(=O)CCC4C3CCC12C)C#C | 1 |
| CC(CS)C(=O)N1CCCC1C(=O)O | 1 |
| COc1ccc2C3CCC4(C)C(CCC4=O)C3CCc2c1 | 1 |
| CC(=O)OC1C(=O)C2(C)C(O)CC3OCC3(OC(=O)C)C2C(OC(=O)c4ccccc4)C5(O)CC(OC(=O)C(O)C(NC(=O)c6ccccc6)c7ccccc7)C(=C1C5(C)C)C | 1 |
| CC1(O)CCC2C3CCC4CC(=O)CCC4(C)C3CCC12C | 1 |
| CC12CCC3C(CCC4=CC(=O)CCC34C)C1CCC2O | 1 |
| NNC(=O)c1ccncc1 | 1 |
| CCCCCCCCCCC(=O)OC1CCC2C3CCC4=CC(=O)CCC4(C)C3CCC12C | 1 |
| CCCCCCCCCC(=O)OC1CCC2C3CCC4=CC(=O)CCC4C3CCC12C | 1 |
| CC12CCC3C(=CCc4cc(O)ccc34)C1CCC2=O | 1 |
| CN1C(=C(O)c2ccccc2S1(=O)=O)C(=O)Nc3ccccn3 | 1 |
| CC(=O)Nc1nnc(s1)S(=O)(=O)N | 1 |
| CCCCCCCC(=O)OCC(=O)C1(O)CCC2C3CCC4=CC(=O)CCC4(C)C3C(O)CC12C | 1 |
| ClCCNCCCl | 1 |
| COc1ccc2C3CCC4(C)C(CCC4(O)C#C)C3CCc2c1 | 1 |
| CC12CCC3C(CCC4CC(=O)CCC34C)C1CCC2O | 1 |
| CCC(=O)OC1(C(C)CC2C3CC(F)C4=CC(=O)C=CC4(C)C3(F)C(O)CC12C)C(=O)SCF | 1 |
| BrCCOCCBr | 1 |
| CCCCCCC(=O)OC1(CCC2C3CCC4=CC(=O)CCC4C3CCC12C)C#C | 1 |
| CC12CCC3C(CCC4=C3CCC(=O)C4)C1CCC2O | 1 |
| OC(CCN1CCCCC1)(C2CCCCC2)c3ccccc3 | 1 |
| CC(=CCO)C=CC=C(C)C=CC1=C(C)CCCC1(C)C | 1 |
| NC(=O)NO | 1 |
| CC12CCC3C(CCC4=CC(=O)CCC34C)C1CCC2OC(=O)CCC5CCCC5 | 1 |
| CC1CC2C3CCC(O)(C(=O)CO)C3(C)CC(O)C2C4(C)C=CC(=O)C=C14 | 1 |
| COc1cc(Cc2cnc(N)nc2N)cc(OC)c1OC | 1 |
| COC(=O)Nc1nc2cc(ccc2[nH]1)C(=O)c3cccs3 | 1 |
| COc1cc2OC3OC=CC3c2c4OC(=O)C5=C(CCC5=O)c14 | 1 |
| CCC(C)C(=O)OC1CCC=C2C=CC(C)C(CCC3CC(O)CC(=O)O3)C12 | 1 |
| COC1=CC2=CCC3C4CCC(=O)C4(C)CCC3C2(C)CC1 | 1 |
| COc1ccnc(CS(=O)C2Nc3ccc(OC(F)F)cc3N2)c1OC | 1 |
| CC1CC2C(CCC3(C)C2CCC3(OC(=O)C)C(=O)C)C4(C)CCC(=O)C=C14 | 1 |
| CC1CC2C3CC(F)C4=CC(=O)C=CC4(C)C3(Cl)C(O)CC2(C)C1C(=O)COC(=O)C(C)(C)C | 1 |
| CC12CCC3C(CCC4=C3CCC(=O)C4)C1CCC2=O | 1 |
| CCOC(=O)C=C(C)C=CC=C(C)C=Cc1c(C)cc(OC)c(C)c1C | 1 |
| Nc1nc(N)c2nc(CNc3ccc(cc3)C(=O)NC(CCC(=O)O)C(=O)O)cnc2n1 | 1 |
| CCCCC(CC)C(=O)O | 1 |
| NC(Cc1ccc(cc1)N(CCCl)CCCl)C(=O)O | 1 |
| CCC(=O)C(CC(C)N(C)C)(c1ccccc1)c2ccccc2 | 1 |
| NS(=O)(=O)c1cc(C(=O)O)c(NCc2occc2)cc1Cl | 1 |
| COc1cc(C)c(C=CC(=CC=CC(=CC(=O)O)C)C)c(C)c1C | 1 |
| CC(=O)OC1(CCC2C3C=C(C)C4=CC(=O)CCC4C3CCC12C)C(=O)C | 1 |
| CC(=O)OC1(CCC2C3CC(=C)C4=CC(=O)CCC4C3CCC12C)C(=O)C | 1 |
| CC1(C)OC2CC3C4CCC5=CC(=O)C=CC5(C)C4(F)C(O)CC3(C)C2(O1)C(=O)COP(=O)(O)O | 1 |
| Cc1cc(cc(c1O)[N+](=O)[O-])[N+](=O)[O-] | 1 |
| CC12CCC3C(CCC4=CC(=O)CCC34)C1CCC2(O)C#C | 1 |
| CC12CCc3c(ccc4cc(OS(=O)(=O)O)ccc34)C1CCC2O | 1 |
| CCCCC(=O)OC1(C(C)CC2C3CCC4=CC(=O)C=CC4(C)C3(F)C(O)CC12C)C(=O)CO | 1 |
| COCCOc1cc2ncnc(Nc3cccc(c3)C#C)c2cc1OCCOC | 1 |
| CC(C)(C)NC(=O)C1CCC2C3CCC4NC(=O)C=CC4(C)C3CCC12C | 1 |
| FC(F)(F)C1(OC(=O)Nc2ccc(Cl)cc12)C#CC3CC3 | 1 |
| COC1CC(CC(C)C2CC(=O)C(C)C=C(C)C(O)C(OC)C(=O)C(C)CC(C)C=CC=CC=C(C)C(CC3CCC(C)C(O)(O3)C(=O)C(=O)N4CCCCC4C(=O)O2)OC)CCC1OCCO | 1 |
| NC1CCCCC1N | 1 |
| COc1c(C)c2COC(=O)c2c(O)c1CC=C(C)CCC(=O)OCCN3CCOCC3 | 1 |
| CCOc1nc2cccc(C(=O)OC(C)OC(=O)OC3CCCCC3)c2n1Cc4ccc(cc4)c5ccccc5c6nn[nH]n6 | 1 |
| CC(C)c1nc(nc(c2ccc(F)cc2)c1C=CC(O)CC(O)CC(=O)O)N(C)S(=O)(=O)C | 1 |
| Nc1nc(O)c2ncn(COC(CO)CO)c2n1 | 1 |
| OC(Cn1cncn1)(Cn2cncn2)c3ccc(F)cc3F | 1 |
| CC1CNC2C(C1)OC3(CCC4C5CC=C6CC(O)CCC6(C)C5CC4=C3C)C2C | 1 |
| c1ccc2c(c1)c3nc2[nH]c4nc(nc5[nH]c(nc6nc(n3)c7ccccc67)c8ccccc58)c9ccccc49 | 1 |
| CC(c1ncncc1F)C(O)(Cn2cncn2)c3ccc(F)cc3F | 1 |
| CN1N=Nc2c(ncn2C1=O)C(=O)N | 1 |
| COc1cc(cc(OC)c1OP(=O)(O)O)C2C3C(COC3=O)C(OC4OC5COC(C)OC5C(O)C4O)c6cc7OCOc7cc26 | 1 |
| CN1CC(CC2C1Cc3c[nH]c4cccc2c34)C(=O)NC5(C)OC6(O)C7CCCN7C(=O)C(Cc8ccccc8)N6C5=O | 1 |
| CCOC(=O)C(CCc1ccccc1)NC2CCc3ccccc3N(CC(=O)O)C2=O | 1 |
| CC(C)n1c(C=CC(O)CC(O)CC(=O)O)c(c2ccc(F)cc2)c3ccccc13 | 1 |
| Cn1c(CCCC(=O)O)nc2cc(ccc12)N(CCCl)CCCl | 1 |
| COC(=O)C1C2CCC(CC1OC(=O)c3ccccc3)N2 | 1 |
| CCNC(=O)C1CCCN1C(=O)C(CCCN=C(N)N)NC(=O)C(NC(=O)C(NC(=O)C(Cc2ccc(O)cc2)NC(=O)C(CO)NC(=O)C(Cc3c[nH]c4ccccc34)NC(=O)C(Cc5c[nH]cn5)NC(=O)C6CCC(=O)N6)C(C)C)C(C)C | 1 |
| CC(C)CC(NC(=O)C(CCCCNC(=O)c1cccnc1)NC(=O)C(CCCCNC(=O)c2cccnc2)NC(=O)C(CO)NC(=O)C(Cc3cccnc3)NC(=O)C(Cc4ccc(Cl)cc4)NC(=O)C(Cc5ccc6ccccc6c5)NC(=O)C)C(=O)NC(CCCCNC(C)C)C(=O)N7CCCC7C(=O)NC(C)C(=O)N | 1 |
| CC(C)OC(=O)CCCC=CCC1C(O)CC(O)C1CCC(O)CCc2ccccc2 | 1 |
| CC(C)C1NC(=O)C(NC(=O)C2=CC(=N)C(=C3Oc4c(C)c(O)c(N)c(C(=O)NC5C(C)OC(=O)C(C(C)C)N(C)C(=O)CN(C)C(=O)C6CCCN6C(=O)C(NC5=O)C(C)C)c4N=C23)C)C(C)OC(=O)C(C(C)C)N(C)C(=O)CN(C)C(=O)C7CCCN7C1=O | 1 |
| CC(=O)Oc1ccc2C3CCC4(C)C(CCC4=O)C3CCc2c1 | 1 |
| CC(C)CC(NC(=O)C(Cc1c[nH]c2ccccc12)NC(=O)C(Cc3ccc(O)cc3)NC(=O)C(CO)NC(=O)C(Cc4c[nH]c5ccccc45)NC(=O)C(Cc6ccc(F)cc6)NC(=O)C7C=CCN7C(=O)C)C(=O)NC(CCCN=C(N)N)C(=O)N8CCCC8C(=O)NCC(=O)N | 1 |
| CC(C)CC(NC(=O)C(Cc1c[nH]c2ccccc12)NC(=O)CNC(=O)C(Cc3ccc(O)cc3)NC(=O)C(CO)NC(=O)C(Cc4c[nH]c5ccccc45)NC(=O)C(Cc6c[nH]cn6)NC(=O)C7CCC(=O)N7)C(=O)N8CCCC8C(=O)NCC(=O)N | 1 |
| CCNC(=O)C1CCCN1C(=O)C(CCCN=C(N)N)NC(=O)C(CC(C)C)NC(=O)C(Cc2cn(Cc3ccccc3)cn2)NC(=O)C(Cc4ccc(O)cc4)NC(=O)C(CO)NC(=O)C(Cc5c[nH]c6ccccc56)NC(=O)C(Cc7c[nH]cn7)NC(=O)C8CCC(=O)N8 | 1 |
| CCCN1CC(CSC)CC2C1Cc3c[nH]c4cccc2c34 | 1 |
| CCNC(=O)C1CCCN1C(=O)C(CCCN=C(N)N)NC(=O)C(CC(C)C)NC(=O)CNC(=O)C(Cc2ccc(O)cc2)NC(=O)C(CO)NC(=O)C(Cc3c[nH]c4ccccc34)NC(=O)C(Cc5c[nH]cn5)NC(=O)C6CCC(=O)N6 | 1 |
| CC12CCC3C(CCc4cc(OS(=O)(=O)O)ccc34)C1CCC2O | 1 |
| CC(C)CC(NC(=O)C(Cc1c[nH]c2ccccc12)NC(=O)C(Cc3ccc(O)cc3)NC(=O)C(CO)NC(=O)C(Cc4c[nH]c5ccccc45)NC(=O)C(Cc6c[nH]cn6)NC(=O)C7CCC(=O)N7)C(=O)NC(CCCN=C(N)N)C(=O)N8CCCC8C(=O)NCC(=O)N | 1 |
| CCNC(=O)C1CCCN1C(=O)C(CCCN=C(N)N)NC(=O)C(CC(C)C)NC(=O)C(Cc2c[nH]c3ccccc23)NC(=O)C(Cc4ccc(O)cc4)NC(=O)C(CO)NC(=O)C(Cc5c[nH]c6ccccc56)NC(=O)C(Cc7c[nH]cn7)NC(=O)C8CCC(=O)N8 | 1 |
| Cc1cn(cn1)c2cc(NC(=O)c3ccc(C)c(Nc4nccc(n4)c5cccnc5)c3)cc(c2)C(F)(F)F | 1 |
| CCCCCC(C)(O)C=CC1C(O)CC(O)C1CC=CCCCC(=O)O | 1 |
| CCN(CC)CCNC(=O)c1c(C)[nH]c(C=C2C(=O)Nc3ccc(F)cc23)c1C | 1 |
| CCC1=CC2CN(C1)Cc3c([nH]c4ccccc34)C(C2)(C(=O)OC)c5cc6c(cc5OC)N(C)C7C(O)(C(OC(=O)C)C8(CC)C=CCN9CCC67C89)C(=O)OC | 1 |
| CCC12CCC3C(CCC4=CC(=NO)CCC34)C1CCC2(OC(=O)C)C#C | 1 |
| CC(=O)Oc1ccc2C3CCC4(C)C(O)CCC4C3CCc2c1 | 1 |
| CC12CCC3=C4CCC(=O)C=C4CCC3C1CCC2(O)CC#N | 1 |
| CC12CC=C3C(CCC4=CC(=O)CCC34C)C1CCC2=O | 1 |
| Oc1ccc(cc1)c2sc3cc(O)ccc3c2C(=O)c4ccc(OCCN5CCCCC5)cc4 | 1 |
| CC(C)C(N)C(=O)OCC(CO)OCn1cnc2c(O)nc(N)nc12 | 1 |
| CC12CCC3C(CC(=O)c4cc(O)ccc34)C1CCC2O | 1 |
| CCOC(=O)C(CCc1ccccc1)NC(C)C(=O)N2C3CCCC3CC2C(=O)O | 1 |
| CCNC(=O)CCCC=CCC1C(O)CC(O)C1C=CC(O)CCc2ccccc2 | 1 |
| CCC(=C(c1ccccc1)c2ccc(OCCNC)cc2)c3ccccc3 | 1 |
| FCOC(C(F)(F)F)C(F)(F)F | 1 |
| CC(C)CC(NC(=O)C(COC(C)(C)C)NC(=O)C(Cc1ccc(O)cc1)NC(=O)C(CO)NC(=O)C(Cc2c[nH]c3ccccc23)NC(=O)C(Cc4c[nH]cn4)NC(=O)C5CCC(=O)N5)C(=O)NC(CCCN=C(N)N)C(=O)N6CCCC6C(=O)NNC(=O)N | 1 |
| COC(C(Oc1nc(C)cc(C)n1)C(=O)O)(c2ccccc2)c3ccccc3 | 1 |
| CCC(=O)OC1(CCC2C3CC(C)C4=CC(=O)C=CC4(C)C3C(O)CC12C)C(=O)COC(=O)C | 1 |
| CCC(=O)OC1(CCC2C3CCC4=CC(=O)CCC4(C)C3C(O)CC12C)C(=O)COC(=O)C | 1 |
| CC1CC2=CC(=O)CCC2C3CCC4(C)C(O)CCC4C13 | 1 |
| CCCCC(C)(O)CC=CC1C(O)CC(=O)C1CCCCCCC(=O)OC | 1 |
| C=CC(=O)OC1CCC2C3CCC(C3)C12 | 1 |
| N#Cc1ccc(cc1)C(c2ccc(cc2)C#N)n3cncn3 | 1 |
| CCCCCCCCc1ccc(CCC(N)(CO)CO)cc1 | 1 |
| Nc1nc(N)c2nc(CC(CC#C)c3ccc(cc3)C(=O)NC(CCC(=O)O)C(=O)O)cnc2n1 | 1 |
| CCCCC(=O)OCC(=O)C1(O)CC(OC2CC(NC(=O)C(F)(F)F)C(O)C(C)O2)c3c(O)c4C(=O)c5c(OC)cccc5C(=O)c4c(O)c3C1 | 1 |
| ONC(=O)CCCCCCC(=O)Nc1ccccc1 | 1 |
| Oc1cccc(O)c1[N+](=O)[O-] | 1 |
| COc1cccc2C(=O)c3c(O)c4CC(O)(CC(OC5CC(N)C(O)C(C)O5)c4c(O)c3C(=O)c12)C(C)O | 1 |
| CN(C)C1C2CC3Cc4c(cc(NC(=O)CNC(C)(C)C)c(O)c4C(=C3C(=O)C2(O)C(=C(C(=O)N)C1=O)O)O)N(C)C | 1 |
| CC(C)c1c(C(=O)Nc2ccccc2)c(c3ccccc3)c(c4ccc(F)cc4)n1CCC(O)CC(O)CC(=O)O | 1 |
| CCNC(=O)C1CCCN1C(=O)C(CCCN=C(N)N)NC(=O)C(CC(C)C)NC(=O)C(CC(C)C)NC(=O)C(Cc2ccc(O)cc2)NC(=O)C(CO)NC(=O)C(Cc3c[nH]c4ccccc34)NC(=O)C(Cc5c[nH]cn5)NC(=O)C6CCC(=O)N6 | 1 |
| FC(F)OC(F)C(F)(F)F | 1 |
| CCNC(=O)C1CCCN1C(=O)C(CCCN=C(N)N)NC(=O)C(CC(C)C)NC(=O)C(C)NC(=O)C(Cc2ccc(O)cc2)NC(=O)C(CO)NC(=O)C(Cc3c[nH]c4ccccc34)NC(=O)C(Cc5c[nH]cn5)NC(=O)C6CCC(=O)N6 | 1 |
| CC(C)CC(NC(=O)C(CC1=CCc2ccccc12)NC(=O)C(Cc3ccc(O)cc3)NC(=O)C(CO)NC(=O)C(CCc4ccccc4NC=C)NC(=O)C(CC(=O)C5CCC(=O)N5)Cc6c[nH]cn6)C(=O)NC(CCCN=C(N)N)C(=O)CN7CCCC7C(=O)NCC(=O)N | 1 |
| O=C1C=CC=C2C3CC(CN12)C4CCCCN4C3 | 1 |
| CC(=O)C1(O)CCC2C3C=C(Cl)C4=CC(=O)C5CC5C4(C)C3CCC12C | 1 |
| Nc1cccc2C(=O)N(Cc12)C3CCC(=O)NC3=O | 1 |
| CC(C)CC(NC(=O)CNC(=O)C(Cc1ccc(O)cc1)NC(=O)C(CO)NC(=O)C(Cc2c[nH]c3ccccc23)NC(=O)C(Cc4c[nH]cn4)NC(=O)C5CCC(=O)N5)C(=O)NC(CCC(=O)N)C(=O)N6CCCC6C(=O)NCC(=O)N | 1 |
| CC(C)CC(NC(=O)C(CCCN=C(N)N)NC(=O)C(Cc1ccc(O)cc1)NC(=O)C(CO)NC(=O)C(Cc2c[nH]c3ccccc23)NC(=O)C(N)Cc4ccc(Cl)cc4)C(=O)NC(CCCN=C(N)N)C(=O)N5CCCC5C(=O)NC(C)C(=O)NC(=O)C(Cc6c[nH]c7ccccc67)NC(=O)C | 1 |
| Cc1c(c2ccc(O)cc2)n(Cc3ccc(OCCN4CCCCCC4)cc3)c5ccc(O)cc15 | 1 |
| CCN(C(CC(C)C)C(=O)N1CCCC1C(=O)N)C(=O)C(Cc2c[nH]c3ccccc23)NC(=O)C(CCCN=C(N)N)NC(=O)C(Cc4ccc(O)cc4)NC(=O)C(CO)NC(=O)C(Cc5c[nH]c6ccccc56)NC(=O)C(Cc7c[nH]cn7)NC(=O)C8CCC(=O)N8 | 1 |
| Cn1cnc(c1N)[N+](=O)[O-] | 1 |
| CCC(=O)NS(=O)(=O)c1ccc(cc1)c2c(C)onc2c3ccccc3 | 1 |
| CCC(=O)OC1(C(C)CC2C3CC(F)C4=CC(=O)C=CC4(C)C3(F)C(O)CC12C)C(=O)SC | 1 |
| COc1ccc(cc1OC)C(=O)OC2CC3CCC(C2)N3 | 1 |
| CON(C)C(=O)Nc1ccc(Cl)c(Cl)c1 | 1 |
| CP(=O)(O)CCC(N)C(=O)O | 1 |
| ClCCOP(=O)(OCCCl)OCCCl | 1 |
| Cc1cc(C)cc(OP(=O)(Oc2cc(C)cc(C)c2)Oc3cc(C)cc(C)c3)c1 | 1 |
| CCCCCCCCCc1ccc(OP(Oc2ccc(CCCCCCCCC)cc2)Oc3ccc(CCCCCCCCC)cc3)cc1 | 1 |
| O=S1(=O)CCCC1 | 1 |
| Cc1ccc(cc1)S(=O)(=O)O | 1 |
| OC(=O)c1ccccc1 | 1 |
| CCCCCCCC(=O)O | 1 |
| OCl(=O)=O | 1 |
| OBr(=O)=O | 1 |
| CC(C)CCCCCC(=O)O | 1 |
| CC(C)(C)c1cc(cc(c1O)C(C)(C)C)C(=O)O | 1 |
| CCC(=O)O | 1 |
| CC(C)(C)CCCCCC(=O)O | 1 |
| Cc1ccc(C)cc1 | 1 |
| CCCCCCC | 1 |
| Cc1cccc(C)c1 | 1 |
| c1ccc2c(c1)cc3ccc4cccc5ccc2c3c45 | 1 |
| C=Cc1ccccc1 | 1 |
| ClCC(Cl)CCl | 1 |
| CCCBr | 1 |
| Brc1cc(Oc2cc(Br)c(Br)c(Br)c2Br)c(Br)c(Br)c1Br | 1 |
| COCCO | 1 |
| CCOCCO | 1 |
| CCCCOCC(C)O | 1 |
| COCCOC | 1 |
| OCC1CCCO1 | 1 |
| CO | 1 |
| COC(C)CO | 1 |
| COCCOCCOC | 1 |
| COCCOCCOCCOC | 1 |
| NCCNCCO | 1 |
| Oc1ccc(O)cc1 | 1 |
| CCC(=C(c1ccc(OCCN(C)C)cc1)c2cccc(O)c2)c3ccccc3 | 1 |
| Oc1c(Cl)cc(Cl)c(Cl)c1Cl | 1 |
| Oc1ccccc1 | 1 |
| Oc1c(C=C)c(C=C)cc(C=C)c1C=C | 1 |
| CC(C)(c1ccc(O)cc1)c2ccc(O)cc2 | 1 |
| CCC(C)C(C)C(C)CCc1ccc(O)cc1 | 1 |
| Clc1ccc(cc1)C(C(=O)C2C(=O)c3ccccc3C2=O)c4ccccc4 | 1 |
| CCC(C)CC(C)CC(C)CCc1ccc(O)cc1 | 1 |
| C1COCO1 | 1 |
| CCOCCOC(=O)C | 1 |
| OC1=C(C2CCCc3ccccc23)C(=O)Oc4ccccc14 | 1 |
| COCCOC(=O)C | 1 |
| CCOC(=O)C(=O)OCC | 1 |
| OC1=C(C2CC(Cc3ccccc23)c4ccc(cc4)c5ccccc5)C(=O)Oc6ccccc16 | 1 |
| CCCCC(CC)COC(=O)CSCc1cc(c(O)c(c1)C(C)(C)C)C(C)(C)C | 1 |
| COCCOC(=O)c1ccccc1C(=O)OCCOC | 1 |
| CCCCOC(=O)C(C)Oc1ccc(Oc2ccc(cn2)C(F)(F)F)cc1 | 1 |
| COCC(=O)O | 1 |
| CC1(OC(=O)N(C1=O)c2cc(Cl)cc(Cl)c2)C=C | 1 |
| CCCCOC(=O)c1ccccc1C(=O)OCCCC | 1 |
| CC(C)CCOC(=O)c1ccccc1C(=O)OCCC(C)C | 1 |
| OC1=C(C2CC(Cc3ccccc23)c4ccc(OCc5ccc(cc5)C(F)(F)F)cc4)C(=O)Oc6ccccc16 | 1 |
| CC(C)CCc1ccc(C(=O)O)c(C(=O)O)c1CCC(C)C | 1 |
| CC(C)COC(=O)c1ccccc1C(=O)OCC(C)C | 1 |
| CC(C)CCCCOC(=O)c1ccccc1C(=O)OCCCCC(C)C | 1 |
| OS(=O)(=O)C(F)(F)C(F)(F)C(F)(F)C(F)(F)C(F)(F)C(F)(F)C(F)(F)C(F)(F)F | 1 |
| CCCCCCOC(=O)c1ccccc1C(=O)OCCCCCC | 1 |
| OC(=O)C(F)(F)C(F)(F)C(F)(F)C(F)(F)C(F)(F)C(F)(F)C(F)(F)F | 1 |
| CC(C)CCCOC(=O)c1ccccc1C(=O)OCCCC(C)C | 1 |
| OC(CC(C1=C(O)Oc2ccccc2C1=O)c3ccccc3)c4ccc(cc4)c5ccc(Br)cc5 | 1 |
| OC1=C(C2CC(Cc3ccccc23)c4ccc(cc4)c5ccc(Br)cc5)C(=O)Sc6ccccc16 | 1 |
| OC(=O)C(F)(F)C(F)(F)C(F)(F)C(F)(F)C(F)(F)C(F)(F)C(F)(F)C(F)(F)F | 1 |
| OC(=O)C(F)(F)C(F)(F)C(F)(F)C(F)(F)C(F)(F)C(F)(F)C(F)(F)C(F)(F)C(F)(F)F | 1 |
| O=C(OC1CCCCC1)c2ccccc2C(=O)OC3CCCCC3 | 1 |
| [O-][N+](=O)c1ccccc1 | 1 |
| CCC(C)c1cc(cc(c1OC(=O)C=C(C)C)[N+](=O)[O-])[N+](=O)[O-] | 1 |
| CC(C)(C)c1cc(cc(c1O)[N+](=O)[O-])[N+](=O)[O-] | 1 |
| CC(=O)OC[N-][N+](=O)C | 1 |
| [O-][N+](=O)c1ccc(Oc2ccc(Cl)cc2Cl)cc1 | 1 |
| CCC(C)c1cc(cc(c1O)[N+](=O)[O-])[N+](=O)[O-] | 1 |
| CCCCN | 1 |
| NCCNCCN | 1 |
| Clc1cc(Cl)c(cc1OCC#C)N2N=C3CCCCN3C2=O | 1 |
| CCCOCC(=Nc1ccc(Cl)cc1C(F)(F)F)n2ccnc2 | 1 |
| S=C1NCCN1 | 1 |
| CCCCCCCCCCCCCN1CC(C)OC(C)C1 | 1 |
| CCCCNC(=O)n1c(NC(=O)OC)nc2ccccc12 | 1 |
| COC(=O)Nc1nc2ccccc2[nH]1 | 1 |
| Fc1cc2OCC(=O)N(CC#C)c2cc1N3C(=O)C4=C(CCCC4)C3=O | 1 |
| CC1(C)CNC(=NC1)NN=C(C=Cc2ccc(cc2)C(F)(F)F)C=Cc3ccc(cc3)C(F)(F)F | 1 |
| CC1(C)CCC(=Cc2ccc(Cl)cc2)C1(O)Cn3cncn3 | 1 |
| CC(C)(C)C(O)C(Oc1ccc(Cl)cc1)n2cncn2 | 1 |
| Clc1ccc(CN2CCSC2=NC#N)cn1 | 1 |
| Oc1cccc2cccnc12 | 1 |
| NC=O | 1 |
| CNC=O | 1 |
| CNC(=O)C | 1 |
| CCNC(=O)C(C)OC(=O)Nc1ccccc1 | 1 |
| CC(C1CC1)C(O)(Cn2cncn2)c3ccc(Cl)cc3 | 1 |
| CNC1C(O)C(OC2C(N)CC(N)C(OC3OC(=CCC3N)CN)C2O)OCC1(C)O | 1 |
| OS(=O)(=S)O | 1 |
| CCCCCC(O)C=CC1C(O)CC(=O)C1CCCCCCC(=O)O | 1 |
| FC(F)(F)C(=O)C(F)(F)F | 1 |
| CC1CC2=C(CCC(=O)C2)C3CCC4(C)C(CCC4(O)C#C)C13 | 1 |
| CN(N=O)C(=O)N | 1 |
| CC12CCC3C(CCC4=CC(=O)CCC34C)C1CCC2OC(=O)CCc5ccccc5 | 1 |
| CC(C)CCC(=O)OC1CCC2C3CCC4=CC(=O)CCC4(C)C3CCC12C | 1 |
| CCCP(=O)(OC)OC | 1 |
| CC(C)(C(=O)O)c1ccc(cc1)C(O)CCCN2CCC(CC2)C(O)(c3ccccc3)c4ccccc4 | 1 |
| NN=C(N)N | 1 |
| OCC(NC(=O)C(Cl)Cl)C(O)c1ccc(cc1)[N+](=O)[O-] | 1 |
| NC1=NC(=O)N(C=N1)C2OC(CO)C(O)C2O | 1 |
| CCCCCC(O)C=CC1C(O)CC(O)C1CC=CCCCC(=O)O | 1 |
| CC(C)Cc1ccc(cc1)C(C)C(=O)O | 1 |
| CCC1(O)CC2CN(CCc3c([nH]c4ccccc34)C(C2)(C(=O)OC)c5cc6c(cc5OC)N(C=O)C7C(O)(C(OC(=O)C)C8(CC)C=CCN9CCC67C89)C(=O)OC)C1 | 1 |
| NC(=O)c1ccc(N)nc1 | 1 |
| CC(=O)c1cc2ccccc2s1 | 1 |
| CCCCCCCCCCCCCCCCCC(=O)NCCNCCO | 1 |
| CCC1CCC(CCC(=O)O)C1 | 1 |
| [2H]c1c([2H])c([2H])c(c([2H])c1[2H])[N+](=O)[O-] | 1 |
| COC12CCC3(CC1C(C)(O)C(C)(C)C)C4Cc5ccc(O)c6OC2C3(CCN4CC7CC7)c56 | 1 |
| OCC1OC(OC(C#N)c2ccccc2)C(O)C(O)C1O | 1 |
| CC(C)c1cccc(C(C)C)c1N=C=Nc2c(cccc2C(C)C)C(C)C | 1 |
| CNCC(O)c1ccc(O)c(O)c1 | 1 |
| NCC(O)c1ccc(O)c(O)c1 | 1 |
| C=CC(=O)NCNC(=O)C=C | 1 |
| NC1=NC(=O)N(C=C1)C2OC(CO)C(O)C2(F)F | 1 |
| COCCOC(=O)C=C | 1 |
| O=C(n1ccnc1)n2ccnc2 | 1 |
| NC1=NC(=O)N(C=N1)C2CC(O)C(CO)O2 | 1 |
| CCCCCCCCCCN(CCCCCCCCCC)CCCCCCCCCC | 1 |
| CC12CCC3C(CCC4=CC(=O)CCC34)C1CCC2OC(=O)CCc5ccccc5 | 1 |
| CC1CNC2C(C1)OC3(CCC4C5CC=C6CC(O)CCC6(C)C5C(=O)C4=C3C)C2C | 1 |
| OC1N=C(c2ccccc2Cl)c3cc(Cl)ccc3NC1=O | 1 |
| COC(C)(OC)N(C)C | 1 |
| CC(O)Cn1ccnc1 | 1 |
| CC1CC2C3CCC4=CC(=O)C=CC4(C)C3(Cl)C(O)CC2(C)C1(O)C(=O)CO | 1 |
| CCC(C)C1NC(=O)C(Cc2ccc(O)cc2)NC(=O)C(N)CSSCC(NC(=O)C(CC(=O)N)NC(=O)C(CCC(=O)N)NC1=O)C(=O)N3CCCC3C(=O)NC(CC(C)C)C(=O)NCC(=O)N | 1 |
| CCCCCCCCCCCC(=O)OC1CCC2C3CCC4=CC(=O)CCC4C3CCC12C | 1 |
| Cc1ccc(cc1)C(C)(C)C | 1 |
| CC1(C)C(=CC=CC=CC=CC2=[N+](CCCCS(=O)(=O)O)c3ccc4ccccc4c3C2(C)C)N(CCCCS(=O)(=O)O)c5ccc6ccccc6c15 | 1 |
| OC(=O)CC#N | 1 |
| OC[P+](CO)(CO)CO | 1 |
| CCCCCCCC[N+](C)(CCCCCCCC)CCCCCCCC | 1 |
| C1C=CC=C1 | 1 |
| CC[n+]1cccc2ccccc12 | 1 |
| CC(=O)OCC(=O)C1(O)C(CC2C3CCC4=CC(=O)C=CC4(C)C3(F)C(O)CC12C)OC(=O)C | 1 |
| CCCCC(CC)COC(=O)c1cccc(c1)C(=O)OCC(CC)CCCC | 1 |
| [2H]OC([2H])([2H])[2H] | 1 |
| CNC(=O)c1cc(Oc2ccc(NC(=O)Nc3ccc(Cl)c(c3)C(F)(F)F)cc2)ccn1 | 1 |
| CCCCC1C(=O)N(N(C1=O)c2ccccc2)c3ccccc3 | 1 |
| CC12CCC3C(CCC4=CC(=O)CCC34)C1CCC2OC(=O)CCC5CCCCC5 | 1 |
| Clc1ccc(C(=O)OOC(=O)c2ccc(Cl)cc2Cl)c(Cl)c1 | 1 |
| CCCCc1nc(Cl)c(CO)n1Cc2ccc(cc2)c3ccccc3c4nn[nH]n4 | 1 |
| CC12CCC3C(=CCc4cc(OS(=O)(=O)O)ccc34)C1CCC2=O | 1 |
| ClCCNC(=O)N(CCCl)N=O | 1 |
| OCC(=O)O | 1 |
| CC1=NS(=O)(=O)c2cc(Cl)ccc2N1 | 1 |
| Cc1cc(ccc1N=Nc2c(O)c3c(N)cc(cc3cc2S(=O)(=O)O)S(=O)(=O)O)c4ccc(N=Nc5c(O)c6c(N)cc(cc6cc5S(=O)(=O)O)S(=O)(=O)O)c(C)c4 | 1 |
| CC(=O)NO | 1 |
| CC(=C)C(=O)OCC(O)CCl | 1 |
| CCN(CC)C(C)C(=O)c1ccccc1 | 1 |
| CC(C)C1=CC=C(C)CC1 | 1 |
| COc1c2OC(=O)C=Cc2cc3ccoc13 | 1 |
| OC(COc1cccc(Cl)c1)C=CC2C(O)CC(O)C2CC=CCCCC(=O)O | 1 |
| OC1(CCN(CCCC(=O)c2ccc(F)cc2)CC1)c3ccc(Cl)cc3 | 1 |
| C(C=Cc1ccccc1)N2CCN(CC2)C(c3ccccc3)c4ccccc4 | 1 |
| C=CN1CCCC1=O | 1 |
| NC(=O)N1c2ccccc2C=Cc3ccccc13 | 1 |
| CC(=O)OC1(CCC2C3CCC4=CC(=O)CCC4(C)C3(F)C(O)CC12C)C(=O)C | 1 |
| Cc1ncc(CO)c(CO)c1O | 1 |
| CC12CCC3C(CCC4=CCCCC34)C1CCC2(O)C#C | 1 |
| CC(C(=O)O)c1ccc(c(F)c1)c2ccccc2 | 1 |
| CC12CCC3C(=CCc4cc(OS(=O)(=O)O)ccc34)C1CCC2O | 1 |
| FC(F)(F)COc1ccc(OCC(F)(F)F)c(c1)C(=O)NCC2CCCCN2 | 1 |
| CC(NCCc1ccc(O)cc1)C(O)c2ccc(O)cc2 | 1 |
| CCN(CC)CCOc1ccc(cc1)C(=C(Cl)c2ccccc2)c3ccccc3 | 1 |
| CC1OC(CC(N)C1O)OC2CC(O)(Cc3c(O)c4C(=O)c5ccccc5C(=O)c4c(O)c23)C(=O)C | 1 |
| CC(C)CCCCCOC(=O)c1ccccc1C(=O)OCCCCCC(C)C | 1 |
| CN1C(=O)C(O)N=C(c2ccccc2Cl)c3cc(Cl)ccc13 | 1 |
| CC12C=CC3=C4CCC(=O)C=C4CCC3C1CCC2(O)CC=C | 1 |
| CC(NC=O)C#N | 1 |
| C=CNC=O | 1 |
| CCCCCCCCCCCCCCCCCC1=NCCN1CCO | 1 |
| CCC(COC(=O)C(=C)C)(COC(=O)C(=C)C)COC(=O)C(=C)C | 1 |
| Cc1onc(NS(=O)(=O)c2ccc(N)cc2)c1 | 1 |
| CN1CCC23CCCCC2C1Cc4ccc(O)cc34 | 1 |
| OCCOCCN1CCN(CC1)C(c2ccccc2)c3ccc(Cl)cc3 | 1 |
| Cc1oc(C=O)cc1 | 1 |
| COCCc1ccc(OCC(O)CNC(C)C)cc1 | 1 |
| FC(F)(F)C(F)(F)C(F)(F)C(F)(F)C(F)(F)C(F)(F)C(F)(F)C(F)(F)S(=O)(=O)F | 1 |
| COc1cc2OC3OC=CC3c2c4OC(=O)C5=C(CCC5O)c14 | 1 |
| NC1=NC(=O)N(C=C1)C2OC(CO)C(O)C2O | 1 |
| COc1cccc2C(=O)c3c(O)c4CC(O)(CC(OC5CC(N)C(O)C(C)O5)c4c(O)c3C(=O)c12)C(=O)C | 1 |
| CC(CC(C#N)(c1ccccc1)c2ccccc2)N(C)C | 1 |
| ClCCN(CCCl)P1(=O)OCCCN1CCCl | 1 |
| CC(=O)Oc1c(cc(cc1C(C)(C)C)[N+](=O)[O-])[N+](=O)[O-] | 1 |
| CCC12CC(=C)C3C(CCC4=CCCCC34)C1CCC2(O)C#C | 1 |
| NS(=O)(=O)c1cc(ccc1Cl)C2(O)NC(=O)c3ccccc23 | 1 |
| CCCCCCCCCC(CCCCCCCC(=O)O)S(=O)(=O)O | 1 |
| CC(=C)C(=O)OCC1CCCO1 | 1 |
| CCC(=O)Oc1ccc(cc1)C(=C(CC)c2ccc(OC(=O)CC)cc2)CC | 1 |
| CCCCCCCCCCCC(=O)NCCNCCO | 1 |
| CCC(C)C(=O)OC1CC(C)C=C2C=CC(C)C(CCC3CC(O)CC(=O)O3)C12 | 1 |
| Nc1ccc(cc1)c2nc3cc(N)ccc3[nH]2 | 1 |
| NCC1OC(OC2C(N)CC(N)C(OC3OC(CO)C(O)C(N)C3O)C2O)C(N)CC1O | 1 |
| CCC(C)C1OC2(CCC1C)CC3CC(CC=C(C)C(OC4CC(OC)C(OC5CC(OC)C(O)C(C)O5)C(C)O4)C(C)C=CC=C6COC7C(O)C(=CC(C(=O)O3)C67O)C)O2 | 1 |
| CCCCCCCCCN(CCCCCCCCC)CCCCCCCCC | 1 |
| COc1ccc(cc1)C2Sc3ccccc3N(CCN(C)C)C(=O)C2OC(=O)C | 1 |
| CC12CCC3C(CCC4=CCCCC34)C1CCC2=O | 1 |
| CC1CC2C3CCC4=CC(=O)C=CC4(C)C3(F)C(O)CC2(C)C1(O)C(=O)CCl | 1 |
| OCCNCCNc1ccc(NCCNCCO)c2C(=O)c3c(O)ccc(O)c3C(=O)c12 | 1 |
| CC1CC2C3CCC(O)(C(=O)COC(=O)CCC(=O)O)C3(C)CC(O)C2C4(C)C=CC(=O)C=C14 | 1 |
| CCCC(=O)OC1(C(C)CC2C3CCC4=CC(=O)C=CC4(C)C3(F)C(=O)CC12C)C(=O)CCl | 1 |
| CCC(C)C1NC(=O)C(NC(=O)C2=C(N)C(=O)C(=C3Oc4c(C)ccc(C(=O)NC5C(C)OC(=O)C(C(C)C)N(C)C(=O)CN(C)C(=O)C6CCCN6C(=O)C(NC5=O)C(C)C)c4N=C23)C)C(C)OC(=O)C(C(C)C)N(C)C(=O)CN(C)C(=O)C7CCCN7C1=O | 1 |
| CC1CC2C3CCC4=CC(=O)C=CC4(C)C3(F)C(O)CC2(C)C1(O)C(=O)CO | 1 |
| CC(=O)OC1CCC2(C)C3CCC4(C)C(CCC4=O)C3CC=C2C1 | 1 |
| CC12CCC3C(CCc4cc(OC(=O)N(CCCl)CCCl)ccc34)C1CCC2OP(=O)(O)O | 1 |
| ClCCN(N=O)C(=O)NC1CCCCC1 | 1 |
| CS(=O)(=O)NC(=O)CCCC=CCC1C(C=CC(O)COc2ccccc2)C(O)CC1=O | 1 |
| CC(CSC(=O)c1ccccc1)C(=O)O | 1 |
| CC1CC2C3CC(F)C4=CC(=O)C=CC4(C)C3(F)C(O)CC2(C)C1(OC(=O)C)C(=O)CO | 1 |
| CC(=C)c1cccc(c1)C(=C)C | 1 |
| CCN(CC)CCOc1ccc(cc1)C(=Cc2ccccc2)c3ccccc3 | 1 |
| CCCCCCCCCCCCc1ccc(OP(=S)(S)Oc2ccc(CCCCCCCCCCCC)cc2)cc1 | 1 |
| CCc1c2CN3C(=O)C4=C(C=C3c2nc5ccc(OC(=O)N6CCC(CC6)N7CCCCC7)cc15)C(O)(CC)C(=O)OC4 | 1 |
| CCCC1CC(N(C)C1)C(=O)NC(C(C)Cl)C2OC(SC)C(O)C(O)C2O | 1 |
| Cc1cc(O)cc(C)c1O | 1 |
| CC12CCC3C(CC=C4CC(O)CCC34C)C1CCC2=O | 1 |
| NCCC(O)C(=O)NC1CC(N)C(OC2OC(CN)C(O)C(O)C2O)C(O)C1OC3OC(CO)C(O)C(N)C3O | 1 |
| CN(CCCl)CCCl | 1 |
| CCCCCCCC1=NCCN1CCO | 1 |
| OC1CC(O)C(C=CC2(COc3ccccc3)OCCO2)C1CC=CCCCC(=O)O | 1 |
| CC1CCCC2(C)OC2CC(OC(=O)CC(O)C(C)(C)C(=O)C(C)C1O)C(=Cc3csc(C)n3)C | 1 |
| CN(C)CCc1c[nH]c2ccc(Cn3cncn3)cc12 | 1 |
| CCCCCCCCCC(=O)OC1CCC2C3CCC4=CC(=O)CCC4(C)C3CCC12C | 1 |
| OC(=O)Cc1ccccc1Nc2c(Cl)cccc2Cl | 1 |
| CC1CC2C3CC(F)C4=CC(=O)C=CC4(C)C3(F)C(O)CC2(C)C1(OC(=O)C)C(=O)COC(=O)C | 1 |
| CC(=O)CC(C1=C(O)Oc2ccccc2C1=O)c3ccc(cc3)[N+](=O)[O-] | 1 |
| CC(CN=Cc1ccccc1O)N=Cc2ccccc2O | 1 |
| NCCCn1ccnc1 | 1 |
| NS(=O)(=O)c1cc2c(NCNS2(=O)=O)cc1Cl | 1 |
| CC1(O)CCC2C3CCC4CC(=O)OCC4(C)C3CCC12C | 1 |
| CCCCC1=NC2(CCCC2)C(=O)N1Cc3ccc(cc3)c4ccccc4c5nn[nH]n5 | 1 |
| CN(C)Cc1ccccc1O | 1 |
| CC(C)(C)NCC(O)COc1nsnc1N2CCOCC2 | 1 |
| CCCCCCCCC=CCCCCCCCC(=O)O | 1 |
| Cc1cc(ccc1N=Nc2ccc3c(cc(c(N)c3c2O)S(=O)(=O)O)S(=O)(=O)O)c4ccc(N=Nc5ccc6c(cc(c(N)c6c5O)S(=O)(=O)O)S(=O)(=O)O)c(C)c4 | 1 |
| Cc1ccc(OP(=O)(Oc2ccc(C)cc2)Oc3ccc(C)cc3)cc1 | 1 |
| CC(=CC=O)C=CC=C(C)C=CC1=C(C)CCCC1(C)C | 1 |
| CCC1(O)C(=O)OCC2=C1C=C3N(Cc4cc5c(CN(C)C)c(O)ccc5nc34)C2=O | 1 |
| CC(CCCC(C)(C)O)C1CCC2C(=CC=C3CC(O)CCC3=C)CCCC12C | 1 |
| CCC(N1CCCC1=O)C(=O)N | 1 |
| COc1ccc(CCN(C)CCCC(C#N)(C(C)C)c2ccc(OC)c(OC)c2)cc1OC | 1 |
| CS(=O)(=O)c1ccc2Sc3ccccc3Nc2c1 | 1 |
| NCC1OC(OC2C(N)CC(N)C(O)C2OC3OC(CO)C(O)C3O)C(N)C(O)C1O | 1 |
| CC(O)C(NC(=O)C(C)C(O)C(C)NC(=O)C(NC(=O)c1nc(nc(N)c1C)C(CC(=O)N)NCC(N)C(=O)N)C(OC2OC(CO)C(O)C(O)C2OC3OC(CO)C(O)C(OC(=O)N)C3O)c4c[nH]cn4)C(=O)NCCc5nc(cs5)c6nc(cs6)C(=O)NCCC[S+](C)C | 1 |
| CC(Cc1cccc(c1)C(C)(C)C)C=O | 1 |
| OCNC=O | 1 |
| CC12CCC3C(CCc4cc(O)ccc34)C1CCC2OC(=O)CCc5ccccc5 | 1 |
| CN1CCN(CC1)C(=O)OC2N(C(=O)c3nccnc23)c4ccc(Cl)cn4 | 1 |
| CCC#N | 1 |
| CCCC(=O)OC1(CCC2C3CCC4=CC(=O)CCC4C3CCC12CC)C#C | 1 |
| C=Cn1ccnc1 | 1 |
| Nc1nc(Cl)c2[nH]cnc2n1 | 1 |
| CCCCCCCCN(CCCCCCCC)CCCCCCCC | 1 |
| CCCC(C)C(CCC)C(C)Cc1ccc(OP(=S)(S)Oc2ccc(CC(C)C(CCC)C(C)CCC)cc2)cc1 | 1 |
| CC(CO)Cc1ccc(cc1)C(C)(C)C | 1 |
| CC1CC2(C)C(CCC2(OC(=O)C)C(=O)C)C3CCC4=CC(=O)CCC4C13 | 1 |
| CC12CCC3C(CCc4cc(O)ccc34)C1CCC2OC(=O)CCC5CCCC5 | 1 |
| NCCCC(N)CC(=O)NCC1NC(=O)C(CO)NC(=O)C(N)CNC(=O)C(NC(=O)C(=CNC(=O)N)NC1=O)C2CCN=C(N)N2 | 1 |
| CC(=O)OC1CCC2C3CCC4=CC(=O)CCC4=C3C=CC12C | 1 |
| COc1ncnc(NS(=O)(=O)c2ccc(N)cc2)c1OC | 1 |
| CC(C)NCC(O)COc1ccc(COCCOC(C)C)cc1 | 1 |
| CC12CCC3C(CCC4CC(=O)CCC34C)C1CC=C2 | 1 |
| Nc1nc(F)nc2c1ncn2C3OC(COP(=O)(O)O)C(O)C3O | 1 |
| Cc1oncc1C(=O)Nc2ccc(cc2)C(F)(F)F | 1 |
| CCNc1cc2OC3=CC(=NCC)C(=CC3=C(c4ccccc4C(=O)OCC)c2cc1C)C | 1 |
| CCC(C)c1cc(cc(c1OC(=O)C)[N+](=O)[O-])[N+](=O)[O-] | 1 |
| CN(C)C1C2C(O)C3C(=C(O)C2(O)C(=C(C(=O)N)C1=O)O)C(=O)c4c(O)cccc4C3(C)O | 1 |
| NCC(CC(=O)O)c1ccc(Cl)cc1 | 1 |
| CCC1OC23CCC(O1)(C(=O)C)C2(C)CCC4C3CCC5=CC(=O)CCC45C | 1 |
| Oc1[nH]cnc2nncc12 | 1 |
| OCCN1CCN=C1 | 1 |
| c1ncncn1 | 1 |
| ClCCN(CCCl)C(=O)Cl | 1 |
| CCOC(=O)OC1(CCC2C3CCC4=CC(=O)C=CC4(C)C3C(O)CC12C)C(=O)COC(=O)CC | 1 |
| CC12CCC3C(CCc4cc(OC(=O)N(CCCl)CCCl)ccc34)C1CCC2O | 1 |
| NS(=O)(=O)c1cc2c(NC(Cc3ccccc3)NS2(=O)=O)cc1C(F)(F)F | 1 |
| CN1CCN(CC1)C2=Nc3ccccc3Nc4sc(C)cc24 | 1 |
| CCOC(=O)CCC(NC(=O)c1ccc(cc1)N(C)Cc2cnc3nc(N)nc(N)c3n2)C(=O)OCC | 1 |
| Oc1nc2ccccc2n1CCCN3CCC(CC3)n4c(O)nc5cc(Cl)ccc45 | 1 |
| CN(C)C1C2CC3C(=C(O)C2(O)C(=C(C(=O)N)C1=O)O)C(=O)c4c(O)ccc(Cl)c4C3(C)O | 1 |
| Nc1ccc(O)cc1Cl | 1 |
| COCCOCCOCCOCCOC | 1 |
| CC12CCC3C(CCC4=CC(=O)CCC34)C1CCC2OS(=O)(=O)O | 1 |
| CCC(=O)OCC(=O)C1(OC(=O)CC)C(C)CC2C3CCC4=CC(=O)C=CC4(C)C3(F)C(O)CC12C | 1 |
| Cc1ncc[nH]1 | 1 |
| CCN(N=O)C(=O)N | 1 |
| CC1CC2C3CC(F)C4=CC(=O)C=CC4(C)C3(F)C(O)CC2(C)C1(O)C(=O)CO | 1 |
| OC(=O)C=CC(=O)O | 1 |
| C=CCC#N | 1 |
| CCCCC(CC)COC(=O)Nc1ccc(C)c(NC(=O)OCCN(C)C)c1 | 1 |
| CC1CC2=CC(=O)CCC2C3CCC4(C)C(CCC4(C)O)C13 | 1 |
| CCC(CO)NC(=O)C1CN(C)C2Cc3c[nH]c4cccc(C2=C1)c34 | 1 |
| COP(OC)OC | 1 |
| COc1ccc2CC3C4C=CC(O)C5Oc1c2C45CCN3C | 1 |
| CCCCCCCCC=CCCCCCCCC1=NCCN1CCO | 1 |
| COCC(=O)Nc1cc(Sc2ccccc2)ccc1N=C(NC(=O)OC)NC(=O)OC | 1 |
| CC1CC2C3CCC4=CC(=O)C=CC4(C)C3(F)C(O)CC2(C)C1(O)C(=O)COC(=O)CC(C)(C)C | 1 |
| S=P(N1CC1)(N2CC2)N3CC3 | 1 |
| CN(C)CCOC(c1ccccc1)c2ccccc2 | 1 |
| CC(=O)C1(O)CCC2C3CCC4=CC(=O)CCC4(C)C3CCC12C | 1 |
| COC(=O)c1ccccc1O | 1 |
| CC#CC1(O)CCC2C3CCC4=CC(=O)CCC4=C3C(CC12C)c5ccc(cc5)N(C)C | 1 |
| Clc1ccsc1 | 1 |
| CNC1C(O)C(O)C(CO)OC1OC2C(OC3C(O)C(O)C(N=C(N)N)C(O)C3N=C(N)N)OC(C)C2(O)C=O | 1 |
| NC(Cc1ccc(O)c(O)c1)C(=O)O | 1 |
| CCC1(C(=O)NC(=O)N(C)C1=O)c2ccccc2 | 1 |
| OP(=O)(O)OP(=O)(O)O | 1 |
| CS(=O)(=O)Nc1ccc(cc1Oc2ccccc2)[N+](=O)[O-] | 1 |
| CC12CC1(C)C(=O)N(C2=O)c3cc(Cl)cc(Cl)c3 | 1 |
| CCOCCOCCO | 1 |
| FC(F)(F)C(Cl)Br | 1 |
| CC(=O)OC12COC1CC(O)C3(C)C2C(OC(=O)c4ccccc4)C5(O)CC(OC(=O)C(O)C(NC(=O)OC(C)(C)C)c6ccccc6)C(=C(C(O)C3=O)C5(C)C)C | 1 |
| CC12CCC3C(CC=C4CC(CCC34C)OS(=O)(=O)O)C1CCC2=O | 1 |
| CCCCCCCCCCCCCCCCCCOP1OCC2(COP(OCCCCCCCCCCCCCCCCCC)OC2)CO1 | 1 |
| CCCCC(C)(C)C(O)C=CC1C(O)CC(=O)C1CCCCC=CC(=O)OC | 1 |
| CCCCCCCCCCCCc1ccc(O)cc1 | 1 |
| CC1(C)OC2COC3(COS(=O)(=O)N)OC(C)(C)OC3C2O1 | 1 |
| CC(C)(C#N)c1cc(Cn2cncn2)cc(c1)C(C)(C)C#N | 1 |
| C[N+](C)(C)CCO | 1 |
| CC(C)CCCCCCCOP(OCCCCCCCC(C)C)OCCCCCCCC(C)C | 1 |
| CC1(O)CCC2C3CCC4=CC(=O)CCC4C3CCC12C | 1 |
| CC12CCC(O)CC1CCC3C2CCC4(C)C3CCC4=O | 1 |
| Nc1ccc(cc1)N=Nc2ccc(N)c3cc(ccc23)S(=O)(=O)O | 1 |
| OC(=O)c1nn(Cc2ccc(Cl)cc2Cl)c3ccccc13 | 1 |
| Sc1nc2ccccc2[nH]1 | 1 |
| CC(=O)OC1CCC2C3CCC4(C)C(CCC4(OC(=O)C)C#C)C3CCC2=C1 | 1 |
| CC12CCC3C(CCC4=C3CCC(=O)C4)C1CCC2(O)C#C | 1 |
| CNS(=O)(=O)Cc1ccc2[nH]cc(CCN(C)C)c2c1 | 1 |
| O=[P+](Oc1ccccc1)Oc2ccccc2 | 1 |
| CCC1(O)CCC2C3CCC4=CCCCC4C3CCC12C | 1 |
| CC1(O)CCC2C3CCC4=CC(=O)CCC4(C)C3CCC12C | 1 |
| CC1CC2C3CCC4=CC(=O)C=CC4(C)C3(F)C(O)CC2(C)C1(O)C(=O)COC(=O)CCc5ccccc5 | 1 |
| Oc1ncc(F)c(O)n1 | 1 |
| C=CC(=O)OCC1CCCO1 | 1 |
| CC1OC(C(O)C1O)N2C=C(F)C(=O)NC2=O | 1 |
| NC(=O)C1CCCN1C(=O)C(Cc2c[nH]cn2)NC(=O)C3CCC(=O)N3 | 1 |
| CC(Cc1ccc(cc1)C(C)(C)C)C=O | 1 |
| CCC(=O)OC1(C(C)CC2C3CCC4=CC(=O)C=CC4(C)C3(F)C(O)CC12C)C(=O)CO | 1 |
| [O-][N+](=O)c1cccc2c[nH]nc12 | 1 |
| CC(O)C#N | 1 |
| COc1ccc(C=O)cc1 | 1 |
| CCC(OC)OC(=O)C | 1 |
| CCc1nc(N)nc(N)c1c2ccc(Cl)cc2 | 1 |
| Clc1ccc(C(Cn2ccnc2)OCc3ccsc3Cl)c(Cl)c1 | 1 |
| CC1CC2C3CCC4=CC(=O)C=CC4(C)C3(F)C(O)CC2(C)C1(OC(=O)c5ccccc5)C(=O)CO | 1 |
| COCCOC(=O)CC(=O)C | 1 |
| Brc1c(Br)c(Br)c(Oc2c(Br)c(Br)c(Br)c(Br)c2Br)c(Br)c1Br | 1 |
| CCC(=O)OCC(=O)C1(OC(=O)CC)C(C)CC2C3CCC4=CC(=O)C=CC4(C)C3(Cl)C(O)CC12C | 1 |
| CCC1(O)CC2CN(CCc3c([nH]c4ccccc34)C(C2)(C(=O)OC)c5cc6c(cc5OC)N(C)C7C(O)(C(OC(=O)C)C8(CC)C=CCN9CCC67C89)C(=O)OC)C1 | 1 |
| CC1(C)C2CCC1(C)C(=O)C2 | 1 |
| OCCOCCN1CCN(CC1)C2=Nc3ccccc3Sc4ccccc24 | 1 |
| CCC(C)C1OC2(CC3CC(CC=C(C)C(OC4CC(OC)C(OC5CC(OC)C(O)C(C)O5)C(C)O4)C(C)C=CC=C6COC7C(O)C(=CC(C(=O)O3)C67O)C)O2)C=CC1C | 1 |
| CC(=O)OC1CCC2(C)C3CCC4(C)C(CC=C4c5cccnc5)C3CC=C2C1 | 1 |
| COc1ccccc1Oc2c(NS(=O)(=O)c3ccc(cc3)C(C)(C)C)nc(nc2OCCO)c4ncccn4 | 1 |
| OC(=O)COCCN1CCN(CC1)C(c2ccccc2)c3ccc(Cl)cc3 | 1 |
| Cc1oc(C[N+](C)(C)C)cc1 | 1 |
| CC(=Cc1ccc(cc1)C(=O)O)c2ccc3c(c2)C(C)(C)CCC3(C)C | 1 |
| Cc1cc(C)[n+]([I-][n+]2c(C)cc(C)cc2C)c(C)c1 | 1 |
| CC1(C)NC(=O)N(C1=O)c2ccc(c(c2)C(F)(F)F)[N+](=O)[O-] | 1 |
| CC12CCC3C(CCC4=C(O)C(=O)CCC34C)C1CCC2=O | 1 |
| C[N+](C)(C)N | 1 |
| Brc1ccc2NC(=O)OC(=O)c2c1 | 1 |
| CC1CC2C3CC(F)C4=CC(=O)C=CC4(C)C3(F)C(O)CC2(C)C1(OC(=O)c5occc5)C(=O)SCF | 1 |
| CC(=O)OCC(CCn1cnc2cnc(N)nc12)COC(=O)C | 1 |
| COCCCOc1cc(CC(CC(N)C(O)CC(C(C)C)C(=O)NCC(C)(C)C(=O)N)C(C)C)ccc1OC | 1 |
| CC1CCCC2(C)OC2CC(NC(=O)CC(O)C(C)(C)C(=O)C(C)C1O)C(=Cc3csc(C)n3)C | 1 |
| CCOP(=O)(OCC)C(C)NC(=O)N(CCCl)N=O | 1 |
| Nc1ccccc1NC(=O)c2ccc(CNC(=O)OCc3cccnc3)cc2 | 1 |
| Nc1nc(Cl)nc2c1ncn2C3OC(CO)C(O)C3F | 1 |
| COCCOC(=O)OCCOC | 1 |
| CC(C)C1NC(=O)C(NC(=O)c2ccc(C)c3OC4=C(C)C(=O)C(=C(C(=O)NC5C(C)OC(=O)C(C(C)C)N(C)C(=O)CN(C)C(=O)C6CC(O)CN6C(=O)C(NC5=O)C(C)C)C4=Nc23)N)C(C)OC(=O)C(C(C)C)N(C)C(=O)CN(C)C(=O)C7CCCN7C1=O | 1 |
| CN1CCN(CC1)C(c2ccccc2)c3ccc(Cl)cc3 | 1 |
| CC12CC(O)C3C(CCC4=CC(=O)CCC34C)C1CCC2O | 1 |
| CC1CCC2C(C)C3C(CC4C5CC=C6CC(CCC6(C)C5CCC34C)OC7OC(CO)C(OC8OC(C)C(O)C(O)C8O)C(O)C7OC9OC(C)C(O)C(O)C9O)N2C1 | 1 |
| COC1C(C)OC(OC2CC(C)(O)C(C(=O)OC)c3cc4C(=O)c5c6OC7OC(C)(C(O)C(C7O)N(C)C)c6cc(O)c5C(=O)c4c(O)c23)C(OC)C1(C)OC | 1 |
| CC1CCC(CC1)NC(=O)N(CCCl)N=O | 1 |
| CN1CCC(C(O)C1)c2c(O)cc(O)c3C(=O)C=C(Oc23)c4ccccc4Cl | 1 |
| CC(CN1CC(=O)NC(=O)C1)N2CC(=O)NC(=O)C2 | 1 |
| CC(C)CC(NC(=O)C(CCCN=C(N)N)NC(=O)C(Cc1ccc(O)cc1)NC(=O)C(CO)NC(=O)C(N)Cc2c[nH]c3ccccc23)C(=O)NC(CCCN=C(N)N)C(=O)N4CCCC4C(=O)NC(C)C(=O)NC(=O)C(Cc5ccc(Cl)cc5)NC(=O)C(Cc6ccc(Cl)cc6)NC(=O)C | 1 |
| [O-][N+](=O)c1cccc2c(Br)[nH]nc12 | 1 |
| CC(C)CC(NC(=O)C(Cc1c[nH]c2ccccc12)NC(=O)C(Cc3ccc(O)cc3)NC(=O)C(CO)NC(=O)C(Cc4c[nH]c5ccccc45)NC(=O)C(Cc6ccccc6)NC(=O)C7CCC(=O)N7)C(=O)NC(CCCN=C(N)N)C(=O)N8CCCC8C(=O)NCC(=O)N | 1 |
| CC(C)CC(NC(=O)CNC(=O)C(Cc1ccc(O)cc1)NC(=O)C(CO)NC(=O)C(Cc2c[nH]c3ccccc23)NC(=O)C(Cc4c[nH]cn4)NC(=O)C5CCC(=O)N5)C(=O)NC(CCCN=C(N)N)C(=O)N6CC(O)CC6C(=O)NCC(=O)N | 1 |
| CC(=CC=CC(=CC(=O)Nc1ccc(O)cc1)C)C=CC2=C(C)CCCC2(C)C | 1 |
| CCCC(=O)OC1CC(=O)N(C)c2cc(CC(=CC=CC(OC)C3(O)CC(OC(=O)N3)C(C)C4OC14C)C)cc(OC)c2Cl | 1 |
| CC12CCC3C(CC(O)C4=CC(=O)CCC34C)C1CCC2O | 1 |
| COc1cc2c(CCNC(=O)C)c[nH]c2cc1Cl | 1 |
| Cc1cc(O)cc2OC(=O)c3c(O)cc(O)cc3c12 | 1 |
| CC(C)CC(NC(=O)C(Cc1ccccc1)NC(=O)C(Cc2ccc(O)cc2)NC(=O)C(CO)NC(=O)C3CCCN3C(=O)C(Cc4ccccc4)NC(=O)C5CCC(=O)N5)C(=O)NC(CCCN=C(N)N)C(=O)N6CCCC6C(=O)NCC(=O)N | 1 |
| NCCC(O)C(=O)NC1CC(N)C(OC2OC(CN)C(O)C(O)C2N)C(OC3OC(CO)C(O)C3O)C1O | 1 |
| CN(C)c1ccc(C=Cc2ccc3ccccc3[n+]2C)cc1 | 1 |
| CC(C)CC(NC(=O)CNC(=O)C(Cc1ccc(O)cc1)NC(=O)C(CO)NC(=O)C(Cc2c[nH]c3ccccc23)NC(=O)C(N)Cc4c[nH]cn4)C(=O)NC(CCCN=C(N)N)C(=O)N5CCCC5C(=O)NCC(=O)N | 1 |
| COc1cc2C3CCC4(C)C(O)CCC4C3CCc2cc1O | 1 |
| NC(=O)c1ncn(n1)C2OC(CO)C(O)C2O | 1 |
| CC(C)CC(NC(=O)CNC(=O)C(Cc1ccc(O)cc1)NC(=O)C(CO)NC(=O)C(Cc2c[nH]c3ccccc23)NC(=O)C(Cc4c[nH]cn4)NC(=O)C5CCC(=O)N5)C(=O)NC(CCCN=C(N)N)C(=O)N6CCCC6C(=O)NCC(=O)O | 1 |
| COc1cc(O)c2C(=O)OC3(C)CC(O)C(O)C=C3c2c1 | 1 |
| CCCCCC(O)C=CC1C(CC=CCCCC(=O)O)C(O)CC1=O | 1 |
| CC(C)CC(NC(=O)C(CCCCN)NC(=O)C(Cc1ccc(O)cc1)NC(=O)C(CO)NC(=O)C(Cc2c[nH]c3ccccc23)NC(=O)C(Cc4c[nH]cn4)NC(=O)C5CCC(=O)N5)C(=O)NC(CCCN=C(N)N)C(=O)N6CCCC6C(=O)NCC(=O)N | 1 |
| NC(=O)c1ncn(C2OC(CO)C(O)C2O)c1O | 1 |
| [2H]c1cc2C3CCC4(C)C(O)CCC4C3CCc2c([2H])c1O | 1 |
| CC(C)CC(NC(=O)C(C)NC(=O)C(Cc1ccc(O)cc1)NC(=O)C(CO)NC(=O)C(Cc2c[nH]c3ccccc23)NC(=O)C(Cc4ccccc4)NC(=O)C5CCC(=O)N5)C(=O)NC(CCCN=C(N)N)C(=O)N6CCCC6C(=O)NCC(=O)N | 1 |
| CCCCCN1C(=CC=Cc2oc3ccccc3[n+]2CCCCC)Oc4ccccc14 | 1 |
| CC12CCC3C(CCC4NC(=O)C=CC34C)C1CCC2C(=O)Nc5cc(ccc5C(F)(F)F)C(F)(F)F | 1 |
| COc1cc2CCN(CCCN(C)CC3Cc4cc(OC)c(OC)cc34)C(=O)Cc2cc1OC | 1 |
| CN(CCOc1ccc(NS(=O)(=O)C)cc1)CCc2ccc(NS(=O)(=O)C)cc2 | 1 |
| Oc1ccc2C(C(CCc2c1)c3ccccc3)c4ccc(OCCN5CCCC5)cc4 | 1 |
| CCOc1ccccc1OC(C2CNCCO2)c3ccccc3 | 1 |
| Cc1nc(Nc2ncc(s2)C(=O)Nc3c(C)cccc3Cl)cc(n1)N4CCN(CCO)CC4 | 1 |
| OC(Cn1ccnc1)(P(=O)(O)O)P(=O)(O)O | 1 |
| CC(C)(O)C(=O)Nc1ccc(c(c1)C(F)(F)F)[N+](=O)[O-] | 1 |
| CCC12CCC3C(CCC4=CC(=NO)CCC34)C1CCC2(O)C#C | 1 |
| CS(=O)(=O)CCNCc1oc(cc1)c2ccc3ncnc(Nc4ccc(OCc5cccc(F)c5)c(Cl)c4)c3c2 | 1 |
| CCCCCC(C)(O)C=CC1C(O)CC(O)C1CC=CCCCC(=O)OC | 1 |
| COc1nc(N)nc2c1ncn2C3OC(CO)C(O)C3O | 1 |
| CC12CCC3C(C(CCCCCCCCCS(=O)CCCC(F)(F)C(F)(F)F)Cc4cc(O)ccc34)C1CCC2O | 1 |
| Clc1ccc(Nc2nnc(Cc3ccncc3)c4ccccc24)cc1 | 1 |
| CC12CCC3C(CC(=C)C4=CC(=O)C=CC34C)C1CCC2=O | 1 |
| CCc1cc2C(=NCc3nnc(C)n3c2s1)c4ccccc4Cl | 1 |
| CN(C)CCOc1ccc(cc1)C(=C(CCCl)c2ccccc2)c3ccccc3 | 1 |
| COc1cc2c(Nc3ccc(Br)cc3F)ncnc2cc1OCC4CCN(C)CC4 | 1 |
| OCCOc1ccc(cc1)C(=C(CCCl)c2ccccc2)c3ccccc3 | 1 |
| CCCCCCCCc1cc(cc(c1OC(=O)C=CC)[N+](=O)[O-])[N+](=O)[O-] | 1 |
| COc1cccc2C(=O)c3c(O)c4CC(O)(CC(O)c4c(O)c3C(=O)c12)C(=O)C | 1 |
| CCCCN(CCCC)C(=O)CN1CC(C(C1c2ccc(OC)cc2)C(=O)O)c3ccc4OCOc4c3 | 1 |
| CCCCCC(O)C=CC1C=CC(=O)C1CCCCCCC(=O)O | 1 |
| CC(C)OC(=O)CCCC=CCC1C(O)CC(O)C1C=CC(O)COc2cccc(c2)C(F)(F)F | 1 |
| CN(C)c1ccc(cc1)C2CC3(C)C(CCC3(OC(=O)C)C(=O)C)C4CCC5=CC(=O)CCC5=C24 | 1 |
| Clc1ccccc1C2=NCC(=O)Nc3ccc(Br)cc23 | 1 |
| CC(C)OC(=O)CCCC=CCC1C(O)CC(O)C1C=CC(F)(F)COc2ccccc2 | 1 |
| CCC12C=CC3=C4CCC(=O)C=C4CCC3C1CCC2(O)C#C | 1 |
| Clc1ccc(s1)C(=O)NCC2CN(C(=O)O2)c3ccc(cc3)N4CCOCC4=O | 1 |
| COc1cccc2C(=O)c3c(O)c4CC(O)(CC(O)c4c(O)c3C(=O)c12)C(=O)CO | 1 |
| CC=CC1(O)CCC2C3CCC4=CC(=O)CCC4=C3C(CC12C)c5ccc(cc5)N(C)C | 1 |
| Cc1ccc(cc1)c2cc(nn2c3ccc(cc3)S(=O)(=O)N)C(F)(F)F | 1 |
| COc1ccc(CN2CCN(CC2)C(c3ccc(F)cc3)c4ccc(F)cc4)c(OC)c1OC | 1 |
| CC=C1NC(=O)C2CSSCCC=CC(CC(=O)NC(C(C)C)C(=O)N2)OC(=O)C(NC1=O)C(C)C | 1 |
| CCCCCCCCCCCCCCCC(=O)OC1CCC2C3CCC4=CC(=O)CCC4(C)C3CCC12C | 1 |
| CCNC(=NCC)NCCCCC(NC(=O)C(Cc1ccc(O)cc1)NC(=O)C(CO)NC(=O)C(Cc2cccnc2)NC(=O)C(Cc3ccc(Cl)cc3)NC(=O)C(Cc4ccc5ccccc5c4)NC(=O)C)C(=O)NC(CC(C)C)C(=O)NC(CCCCNC(=NCC)NCC)C(=O)N6CCCC6C(=O)NC(C)C(=O)N | 1 |
| Cn1c(ccc1c2ccc3NC(=S)OC(C)(C)c3c2)C#N | 1 |
| CCCCCCCCCCCCCCCCCCOP(=O)(O)OC1CC[N+](C)(C)CC1 | 1 |
| CN(Cc1cnc2nc(N)nc(N)c2n1)c3ccc(cc3)C(=O)O | 1 |
| COc1cccc(C)c1O | 1 |
| ClCCN(CCCl)CCOC(=O)CCNc1c2ccccc2nc3ccccc13 | 1 |
| CC(C)OC(=O)CCCC=CCC1C(O)CC(O)C1C=CC(O)CCc2ccccc2 | 1 |
| CCN(C(=O)C1=C(O)c2c(Cl)cccc2N(C)C1=O)c3ccccc3 | 1 |
| CCCCC(=O)OCC(=O)C1(O)C(C)CC2C3CCC4=CC(=O)C=CC4(C)C3(F)C(O)CC12C | 1 |
| CCCCCCCC(=O)CCC1C(O)CC(O)C1CC=CCCCC(=O)O | 1 |
| CC1=CC2=C(C=O)C(=O)C(C)(O)C(O)C2=CO1 | 1 |
| COC(=O)C1C(CC2CCC1[N+]2(C)[O-])OC(=O)c3ccccc3 | 1 |
| Nc1cccc2C(=O)N(C3CCC(=O)NC3=O)C(=O)c12 | 1 |
| CN(C)c1ccc(cc1)C2CC3(C)C(CCC3(O)CCCO)C4CCC5=CC(=O)CCC5=C24 | 1 |
| COC(=C1C(C(=C(C)N=C1C)C(=O)OC(C)(C)CN(C)CCC(c2ccccc2)c3ccccc3)c4cccc(c4)[N+](=O)[O-])O | 1 |
| CC1OC(OC2CC(O)(Cc3c(O)c4C(=O)c5ccccc5C(=O)c4c(O)c23)C(=O)CO)C(I)C(O)C1O | 1 |
| NC(Cc1c[nH]cn1)C(=O)NC(Cc2ccc(O)cc2)C(=O)O | 1 |
| COCCCOC | 1 |
| CS(=O)(=O)NC(=O)CBr | 1 |
| OC1=C(C(CC(=O)c2ccc(cc2)c3ccc(Br)cc3)c4ccccc4)C(=O)Oc5ccccc15 | 1 |
| Nc1nc(O)c2c(CCc3ccc(cc3)C(=O)O)c[nH]c2n1 | 1 |
| COC1C=COC2(C)Oc3c(C)c(O)c4c(O)c(NC(=O)C(=CC=CC(C)C(O)C(C)C(O)C(C)C(OC(=O)C)C1C)C)c(C=NN5CCN(C)CC5)c(O)c4c3C2=O | 1 |
| COC1CC2OCC2(OC(=O)C)C3C(OC(=O)c4ccccc4)C5(O)CC(OC(=O)C(O)C(NC(=O)OC(C)(C)C)c6ccccc6)C(=C(C(OC)C(=O)C13C)C5(C)C)C | 1 |
| CC(Oc1ccc(Oc2cnc3cc(Cl)ccc3n2)cc1)C(=O)OCC4CCCO4 | 1 |
| CC1=CC=C(C(=O)Nc2ccc(Oc3cc4cnn(C)c4cc3c5cn[nH]c5)c(F)c2)C(=O)N1c6ccc(F)cc6 | 1 |
| CON(C)Cc1ccc(O)c2C(=C3C(CC4C(N(C)C)C(=O)C(=C(O)C4(O)C3=O)C(=O)N)Cc12)O | 1 |
| CCC(=O)OC1(C(C)CC2C3CC(F)C4=CC(=O)CCC4(C)C3(F)C(O)CC12C)C(=O)SCF | 1 |
| CC(C)CCCCCCC(=O)O | 1 |
| CC=CC(=O)Oc1ccccc1[N+](=O)[O-] | 1 |
| COC(=O)c1ccc2c(C(=Nc3ccc(cc3)N(C)C(=O)CN4CCN(C)CC4)c5ccccc5)c(O)[nH]c2c1 | 1 |
| Cc1c(ccc(c1C2=NOCC2)S(=O)(=O)C)C(=O)c3cnn(C)c3O | 1 |
| Fc1ccc(C(=O)Cn2cncn2)c(F)c1 | 1 |
| CCCCCOC(=O)NC1=NC(=O)N(C=C1F)C2OC(C)C3OC(=O)OC23 | 1 |
| CC(O)(CS(=O)(=O)c1ccccc1)C(=O)Nc2ccc(C#N)c(c2)C(F)(F)F | 1 |
| CCC12CC(=C)C3C(CCC4=CCCCC34)C1CCC2=O | 1 |
| CC(C)(Cc1ccc(Oc2ncccc2C#N)cc1)NCC(O)COc3ccccc3c4cccs4 | 1 |
| OCC1OC(N2C=CC(=O)NC2=O)C(F)(F)C1O | 1 |
| CCCCCC(O)C=CC1C(O)CC(=O)C1CCCCCCC(=O)OCC | 1 |
| Cn1c(CCCC(=O)O)nc2cc(ccc12)N(CCO)CCO | 1 |
| OC(=O)c1ccc2nc(oc2c1)c3cc(Cl)cc(Cl)c3 | 1 |
| CCc1ncncc1F | 1 |
| CCCCCCCCCCC(C)CCCCCCCC | 0 |
| CCCCOCCOCCO | 0 |
| OC1(CCCCC1)C(=O)c2ccccc2 | 0 |
| Cc1ccc(cc1[N+](=O)[O-])S(=O)(=O)C | 0 |
| ClC(=C)C(=C)Cl | 0 |
| CC(=O)Oc1ccc(cc1C2C(=O)Oc3ccc(cc23)C(C)(C)CC(C)(C)C)C(C)(C)CC(C)(C)C | 0 |
| CCCCOCCOCCOC(=O)C | 0 |
| C(Oc1ccc2ccccc2c1)c3ccccc3 | 0 |
| CCCCCCCCCCCCN1CCCC1=O | 0 |
| OCC#CCO | 0 |
| CCCCC(CC)COP(=O)(Oc1ccccc1)Oc2ccccc2 | 0 |
| CSc1ccc(cc1)C(=O)C(C)(C)N2CCOCC2 | 0 |
| Oc1ccc2ccccc2c1 | 0 |
| ClCC1CO1 | 0 |
| CCC(=CCCC(=CC#N)C)C | 0 |
| CC(CCC=C(C)C)CC#N | 0 |
| CC(=C)C(=O)OCCC(F)(F)C(F)(F)C(F)(F)C(F)(F)C(F)(F)C(F)(F)F | 0 |
| OC(=O)c1cc2ccccc2cc1O | 0 |
| CC(C)CCCCCCc1ccc(O)cc1 | 0 |
| CCCCCCCCSCc1cc(C)c(O)c(CSCCCCCCCC)c1 | 0 |
| Oc1c(N=Nc2ccc(cc2)S(=O)(=O)CCOS(=O)(=O)O)c(cc3cc(cc(Nc4nc(Cl)nc(Nc5cccc(c5)S(=O)(=O)O)n4)c13)S(=O)(=O)O)S(=O)(=O)O | 0 |
| CC(=O)Nc1ccc2c(O)c(N=Nc3ccc(cc3)S(=O)(=O)CCOS(=O)(=O)O)c(cc2c1)S(=O)(=O)O | 0 |
| CCN(CCC(C)C)c1ccc2c(Oc3cc(C)c(Nc4ccccc4)cc3C25OC(=O)c6ccccc56)c1 | 0 |
| OC(=O)CCCCCN1C(=O)c2ccccc2C1=O | 0 |
| CC(=CCCC(=CC=CC(=O)C)C)C | 0 |
| CCCC(CC)COC(=O)CCc1cc(c(O)c(c1)C(C)(C)C)C(C)(C)C | 0 |
| CCCCCCCCC=C | 0 |
| BrC1CC(Br)CC(Br)CC(Br)CC(Br)CC(Br)C1 | 0 |
| OCCOCC#CCOCCO | 0 |
| CCC(=O)C=CC1C(=CCCC1(C)C)C | 0 |
| CC(O)CN(CC(C)O)C(=O)CCCCC(=O)N(CC(C)O)CC(C)O | 0 |
| CCCCCCCCCCCCCCCCCCCCc1ccc(cc1)S(=O)(=O)O | 0 |
| CC1(C)CC(CC(C)(C)N1)OC(=O)CCCCCCCCC(=O)OC2CC(C)(C)NC(C)(C)C2 | 0 |
| CCOC(C)(C)C | 0 |
| CC=NO | 0 |
| CC(=NO)C | 0 |
| C=CCOC(=O)CCC1CCCCC1 | 0 |
| CC1(C)CNCC(C)(C)C1OC(=O)CCCCCCCCC(=O)OC2C(C)(C)CNCC2(C)C | 0 |
| Oc1ccccc1C(=O)OCc2ccccc2 | 0 |
| NC(=O)N=NC(=O)N | 0 |
| COC(=O)OC | 0 |
| CCCCCCC(=O)OCC=C | 0 |
| CCC=CCCOC(=O)c1ccccc1O | 0 |
| O=C1CCCCCCCCCCCCCCO1 | 0 |
| CCCCCCOC(=O)c1ccccc1O | 0 |
| CC(=O)Nc1ccc2cc(c(N=Nc3ccc(cc3)S(=O)(=O)CCOS(=O)(=O)O)c(O)c2c1)S(=O)(=O)O | 0 |
| OS(=O)(=O)c1ccc2ccccc2c1 | 0 |
| CCCCCCCCCC(CC)c1ccc(cc1)S(=O)(=O)O | 0 |
| OC#N | 0 |
| OCC#C | 0 |
| CC(C)N | 0 |
| Nc1c(N=Nc2ccc(cc2)S(=O)(=O)CCOS(=O)(=O)O)c(cc3cc(c(N=Nc4ccc(cc4)S(=O)(=O)CCOS(=O)(=O)O)c(O)c13)S(=O)(=O)O)S(=O)(=O)O | 0 |
| C1CCOC1 | 0 |
| OCC1OC2OC3C(O)C(O)C(OC3CO)OC4C(O)C(O)C(OC4CO)OC5C(O)C(O)C(OC5CO)OC6C(O)C(O)C(OC6CO)OC7C(O)C(O)C(OC7CO)OC1C(O)C2O | 0 |
| CN1C(=O)N(C(=O)C=C1C(F)(F)F)c2ccc(Cl)c(c2)C(=O)OC(C)(C)C(=O)OCC=C | 0 |
| NCCCN(CCO)CCO | 0 |
| CCCCCCCCOC(=O)c1ccccc1C(=O)OCc2ccccc2 | 0 |
| C1CCc2ccccc2C1 | 0 |
| CC(C)(C)OOC(C)(C)c1cccc(c1)C(C)(C)OOC(C)(C)C | 0 |
| CCCCCCCCCOC(=O)c1ccccc1C(=O)OCCCCCCCC | 0 |
| C=COCCOCCOC=C | 0 |
| CC1(C)N(CO)C(=O)N(CO)C1=O | 0 |
| C(CCOCC1CO1)COCC2CO2 | 0 |
| Cc1ccc(Nc2ccc(Nc3ccc(C)cc3)c4C(=O)c5ccccc5C(=O)c24)cc1 | 0 |
| Clc1ccc(Cl)cc1 | 0 |
| CC(=C)C(=O)OC1CC2CCC1(C)C2(C)C | 0 |
| COC(C)COC(C)CO | 0 |
| C1COCCO1 | 0 |
| CCOCCCC(C)OCCOCC(C)CO | 0 |
| ClCCOCOCCCl | 0 |
| NC(=N)NC#N | 0 |
| CCOCC(C)O | 0 |
| OCCN(CCO)CCO | 0 |
| CCCOCC(C)O | 0 |
| CCCCOCC(C)OCC(C)O | 0 |
| CC(C)(O)c1cccc(c1)C(C)(C)O | 0 |
| CC(O)COc1ccccc1 | 0 |
| CN(CCO)CCO | 0 |
| CCC(C)(C)OOC(C)(C)CC | 0 |
| c1nc[nH]n1 | 0 |
| CC(CCN)CC(C)(C)CN | 0 |
| OCCNCCO | 0 |
| ClCCOP(=O)(OCCCl)OCC(CCl)(CCl)COP(=O)(OCCCl)OCCCl | 0 |
| FC(F)(F)C(Cl)Cl | 0 |
| CC(C)OC(C)C | 0 |
| OCC(O)COC(=O)CS | 0 |
| CC(C)c1cc(C(C)C)c(N=C=O)c(C(C)C)c1N=C=O | 0 |
| CC(C)CC(=O)CC(C)C | 0 |
| CCCCCCCCCCCC(=O)OCC(O)CO | 0 |
| CCC(C)CCCC(C)CCCC(C)C | 0 |
| CN(C)CCOC(=O)C(=C)C | 0 |
| CC(CCCC(C)(C)O)C=C | 0 |
| CC(=C)C(=O)OCCN1CCNC1=O | 0 |
| CC(C)COC(=O)C(=C)C | 0 |
| Cc1ccccc1OCC2CO2 | 0 |
| CC(C)(N)CO | 0 |
| CC(C)(C)c1ccc(OCC2CO2)cc1 | 0 |
| CCC(N)CO | 0 |
| CCCCCCCCOC(=O)c1ccccc1C(=O)OCCCCCCCC | 0 |
| CCCCCCCCCCCOC(=O)c1ccccc1C(=O)OCCCCCCCCCCC | 0 |
| CC(C)Cl | 0 |
| CCOC(C)(C)CC | 0 |
| NCCO | 0 |
| CCCCC(CC)COC(=O)C=C | 0 |
| CCOCC(C)OC(=O)C | 0 |
| CCCCC(CC)CO | 0 |
| CCCCC(CC)CO[N+](=O)[O-] | 0 |
| CCCCCCCCC(CO)CCCCCC | 0 |
| CC(C)(O)C(=O)c1ccccc1 | 0 |
| OC(=O)CS | 0 |
| OCCOC(=O)C=C | 0 |
| CC(=C)C(=O)OC(=O)C(=C)C | 0 |
| CC(=C)C(=O)OCCO | 0 |
| Cc1c(cc(cc1[N+](=O)[O-])[N+](=O)[O-])[N+](=O)[O-] | 0 |
| Cc1cc(N)ccc1N | 0 |
| OCCc1ccccc1 | 0 |
| Cc1ccccc1S(=O)(=O)N | 0 |
| CC(O)CC(C)(C)O | 0 |
| CCCCCCCCCCC(CO)CCCCCCCC | 0 |
| CCCCC(CC)COC(=O)OOC(C)(C)CC | 0 |
| C(Oc1cccc(c1)N(CC2CO2)CC3CO3)C4CO4 | 0 |
| NCCN1CCNCC1 | 0 |
| CC(C)C(=O)C | 0 |
| COc1ccc(C=CC(=O)OCCC(C)C)cc1 | 0 |
| CC(C)(c1cc(Br)c(O)c(Br)c1)c2cc(Br)c(O)c(Br)c2 | 0 |
| C(Oc1ccc(cc1)N(CC2CO2)CC3CO3)C4CO4 | 0 |
| CC(C)C1=NN(C(=O)NC(C)(C)C)C(=O)N1N | 0 |
| CCC(C)(C)c1ccc(O)cc1 | 0 |
| OCCCCOC(=O)C=C | 0 |
| Cc1ccc(cc1)S(=O)(=O)N=C=O | 0 |
| C[N+]1([O-])CCOCC1 | 0 |
| CC(C)(C)c1ccc(O)c(O)c1 | 0 |
| OCC1OC2OC3C(O)C(O)C(OC3CO)OC4C(O)C(O)C(OC4CO)OC5C(O)C(O)C(OC5CO)OC6C(O)C(O)C(OC6CO)OC7C(O)C(O)C(OC7CO)OC8C(O)C(O)C(OC8CO)OC9C(O)C(O)C(OC9CO)OC1C(O)C2O | 0 |
| CC(C)(C)c1ccc(O)cc1 | 0 |
| CCCCOCCOCCOCOCCOCCOCCCC | 0 |
| OCC1OC2OC3C(O)C(O)C(OC3CO)OC4C(O)C(O)C(OC4CO)OC5C(O)C(O)C(OC5CO)OC6C(O)C(O)C(OC6CO)OC7C(O)C(O)C(OC7CO)OC8C(O)C(O)C(OC8CO)OC1C(O)C2O | 0 |
| CC1=CC(=O)N=S(=O)(O)O1 | 0 |
| Nc1nc(N)nc(n1)c2ccccc2 | 0 |
| CCC12COCN1COC2 | 0 |
| CC(C)CCC(=O)C | 0 |
| CC1(C)COC(=O)C2CC=CCC2C(=O)OC1 | 0 |
| CC(C)CCCCCCCOC(=O)C(=C)C | 0 |
| CCCCCCCCCCCCCCCCCCCCCCCCC=C | 0 |
| CCCCCCC=CCCC=CCCCC | 0 |
| CCCCCCCCCCCCCCCCCCCCC=C | 0 |
| Brc1ccc(Br)c(C=C)c1 | 0 |
| C1CCC2CCCCC2C1 | 0 |
| Oc1cccc(O)c1 | 0 |
| CCCCCCCCCC(=O)O | 0 |
| CCCCCCCCCCCCCCCCCCCCCC(=O)OCC(O)CO | 0 |
| CCc1ccccc1 | 0 |
| CC(CC(C)(C)C)CC(C)(C)CC(C)(C)C | 0 |
| CCCCCCCCCCCCCCCC(=O)OCCOC(=O)CCCCCCCCCCCCCCC | 0 |
| CCCCCCCCCCCCCCC=C | 0 |
| ClC(Cl)c1ccccc1 | 0 |
| OC(=O)CBr | 0 |
| CCCCN(C1CC(C)(C)N(C)C(C)(C)C1)c2nc(NCCCN(CCN(CCCNc3nc(nc(n3)N(CCCC)C4CC(C)(C)N(C)C(C)(C)C4)N(CCCC)C5CC(C)(C)N(C)C(C)(C)C5)c6nc(nc(n6)N(CCCC)C7CC(C)(C)N(C)C(C)(C)C7)N(CCCC)C8CC(C)(C)N(C)C(C)(C)C8)c9nc(nc(n9)N(CCCC)C%10CC(C)(C)N(C)C(C)(C)C%10)N(CCCC)C%11CC(C)(C)N(C)C(C)(C)C%11)nc(n2)N(CCCC)C%12CC(C)(C)N(C)C(C)(C)C%12 | 0 |
| CCCCCCCCCCCCCCCCCC[N+](C)(C)C | 0 |
| CCCCN(C)C | 0 |
| C1CCC(CC1)N(Sc2nc3ccccc3s2)C4CCCCC4 | 0 |
| CC(C)N(C)C | 0 |
| CCCCCCCCCCCCCCCC[N+](C)(C)C | 0 |
| CCCCCCCCCCCCN(CCCN)CCCN | 0 |
| CCCCCCCCCC[N+](C)(C)CCCCCCCCCC | 0 |
| CC(C)NC(C)C | 0 |
| CCNc1nc(NC(C)C)nc(SC)n1 | 0 |
| CNc1ccccc1 | 0 |
| CCN(C(C)C)C(C)C | 0 |
| CCCSP(=O)(OCC)Oc1ccc(Br)cc1Cl | 0 |
| CCCCCCC(O)CCCCCCCCCCC(=O)OCC(O)CO | 0 |
| CCCCCCCCCCCCCCCCC=C | 0 |
| CCC(C)(CCC(C)C)C(=O)OCC1CO1 | 0 |
| C1CO1 | 0 |
| NC(=O)C=C | 0 |
| OCCCOC(=O)C=C | 0 |
| CC(C)(C)OOC(C)(C)c1ccc(cc1)C(C)(C)OOC(C)(C)C | 0 |
| CC1(C)C2CCC1(C)C(C2)OC(=O)C=C | 0 |
| CC(C)C1=CC2=CCC3C(C)(CCCC3(C)C(=O)O)C2CC1 | 0 |
| CCCN(CCC)C(=O)SCC | 0 |
| COCC(C)OCC(C)OC(C)CO | 0 |
| CC(C)C(C)(C)C(C)(C)C(C)(C)S | 0 |
| CC#N | 0 |
| CC(=C)C(=O)OCC=C | 0 |
| OC(=O)C=C | 0 |
| C=CC#N | 0 |
| NC(CC(=O)O)C(=O)O | 0 |
| CC(=C)C(=O)OCc1ccccc1 | 0 |
| CCOC(=O)CC(NC1CCC(CC2CCC(NC(CC(=O)OCC)C(=O)OCC)C(C)C2)CC1C)C(=O)OCC | 0 |
| O=C(OCC1CO1)c2ccc(cc2)C(=O)OCC3CO3 | 0 |
| CCCCCC(CCC)COC(=O)c1ccccc1C(=O)OCC(CCC)CCCCC | 0 |
| CCCCC(CC)COC(=O)c1ccc(cc1)C(=O)OCC(CC)CCCC | 0 |
| CCCC(=O)O | 0 |
| CCCCOC(=O)C | 0 |
| CCCCO | 0 |
| CCCCOC(=O)C=C | 0 |
| CCCCOC(=O)C(=C)C | 0 |
| Clc1ccccc1 | 0 |
| OC(=O)C1CCC(CC1)C(=O)O | 0 |
| CCCCCCCC=CC | 0 |
| CC(=C)C(=O)OC1CCCCC1 | 0 |
| CCCCCCCCCCOC(=O)C(=C)C | 0 |
| OC(=O)CSSCC(=O)O | 0 |
| CCCCOC(=O)c1ccc(cc1)C(=O)OCCCC | 0 |
| CCCCCCCCCC(=O)OOC(=O)CCCCCCCCC | 0 |
| CSSC | 0 |
| CCCCCCCCCCCCCCCCCCCCCCOC(=O)C=C | 0 |
| CCCCCCCCCCC=C | 0 |
| O=C(OOC(=O)c1ccccc1)c2ccccc2 | 0 |
| CCCCCCCCCCCCOC(=O)C(=C)C | 0 |
| O=C(c1ccccc1)c2ccccc2 | 0 |
| CCCCCCCCCCCCCCCCCC(=O)OCCOC(=O)CCCCCCCCCCCCCCCCC | 0 |
| CCCCC(CC)C(=O)OCCOCCOCCOC(=O)C(CC)CCCC | 0 |
| CCOC(=O)C(=C)C | 0 |
| CCCCCC(=O)C | 0 |
| CCOC(=O)C=C | 0 |
| O=CCCCC=O | 0 |
| CCCCC=C | 0 |
| CCCCCCCCCCCCCCCC | 0 |
| CCCCCCCCCCCCCCCCOC(=O)C(=C)C | 0 |
| CC=CC=CC(=O)O | 0 |
| CCCCCCCCCCCCCCCCCCCC | 0 |
| CCCCCCCCCCCCCCCCCCCCOC(=O)C=C | 0 |
| CI | 0 |
| CC(C)COC(=O)C=C | 0 |
| OC(=O)c1cccc(c1)C(=O)O | 0 |
| CN=C=S | 0 |
| CC(=C)C(=O)O | 0 |
| CC(CO)OC(=O)C(=C)C | 0 |
| COC=O | 0 |
| COC(=O)C=C | 0 |
| COC(=O)C(=C)C | 0 |
| CC1CCCCC1 | 0 |
| CCCCCCCC=C | 0 |
| CCCCCCCCCCCCCCCCCCCCCCCCCCC=C | 0 |
| CCCCCCCCCCCCCCCCCC | 0 |
| CCCCCCCCOC(=O)C(C)Oc1ccc(Cl)cc1C | 0 |
| CCCCCCC=C | 0 |
| CCCCCCCCCCCCCCCCCCOC(=O)C(=C)C | 0 |
| O=C(OCCOCCOC(=O)c1ccccc1)c2ccccc2 | 0 |
| O=CC=O | 0 |
| CCCC(O)CO | 0 |
| CCCCCCCCCCCCCCC | 0 |
| C1CNCCN1 | 0 |
| CCCCCCC(=O)OCC(COC(=O)CCCCCC)OC(=O)CCCCCC | 0 |
| CCCCCCCCC=CCCCCCCCC(=O)OCC(COC(=O)CCCCCCCC=CCCCCCCCC)OC(=O)CCCCCCCC=CCCCCCCCC | 0 |
| CCCCCCCCCCCCCCCCCC(=O)OCC(COC(=O)CCCCCCCCCCCCCCCCC)OC(=O)CCCCCCCCCCCCCCCCC | 0 |
| CCCOC(=O)C | 0 |
| OCC(=O)Oc1ccc(Cl)cc1Cl | 0 |
| COc1cc(OC)nc(NC(=O)N=S(=O)(O)c2ncccc2OCC(F)(F)F)n1 | 0 |
| CCCCCCCCCCCC(=O)N(C)CC(=O)O | 0 |
| CC(C)(C)OC(=O)C=C | 0 |
| ClC(Cl)(Cl)Cl | 0 |
| CC(=C)C(=O)OC(C)(C)C | 0 |
| CCCCCCCCCCCCCCOC(=O)C(=C)C | 0 |
| CCCCCCCCCCCC=CC | 0 |
| CCCCCCCCCCCCC | 0 |
| CCCCOCCOP(=O)(OCCOCCCC)OCCOCCCC | 0 |
| CCCCCC(C)OC(=O)COc1ccc(Cl)c2cccnc12 | 0 |
| CC1COc2ccccc2N1C(=O)C(Cl)Cl | 0 |
| CC(C)(CS(=O)(=O)O)NC(=O)C=C | 0 |
| CC(=CCCC(=CC=O)C)C | 0 |
| CC(CCC=C(C)C)CC=O | 0 |
| Cc1ccc(cc1)C(=O)OOC(=O)c2ccc(C)cc2 | 0 |
| C=COC1CCCCC1 | 0 |
| C(COc1ccccc1)Oc2ccccc2 | 0 |
| CCCCOCCOCCOCCCC | 0 |
| CC(c1ccccc1)c2ccccc2 | 0 |
| O=S(=O)(c1ccccc1)c2ccccc2 | 0 |
| CC(O)COc1ccc(cc1)C(C)(C)c2ccc(OCC(C)O)cc2 | 0 |
| FC(F)(F)C(F)(F)C(F)(F)N(C(F)(F)C(F)(F)C(F)(F)F)C(F)(F)C(F)(F)C(F)(F)F | 0 |
| CC1C(C)(C)C2=C(C(=O)CCC2)C1(C)C | 0 |
| CCC(C)(C)OO | 0 |
| Cc1cccc(N)c1N | 0 |
| C=CCOC(=O)c1ccccc1C(=O)OCC=C | 0 |
| CC12CCC(CC1)C(C)(C)O2 | 0 |
| CC(C)(N=C=O)c1cccc(c1)C(C)(C)N=C=O | 0 |
| NCc1cccc(CN)c1 | 0 |
| CNC(=O)NC | 0 |
| CCN(C(=O)N(CC)c1ccccc1)c2ccccc2 | 0 |
| O=C(c1ccc(Oc2ccccc2)cc1)c3ccc(cc3)C(=O)c4ccc(Oc5ccccc5)cc4 | 0 |
| N=C(Nc1ccccc1)Nc2ccccc2 | 0 |
| ClCCCCCCCl | 0 |
| Cc1cc(C)c(Nc2ccc(Nc3c(C)cc(C)cc3C)c4C(=O)c5ccccc5C(=O)c24)c(C)c1 | 0 |
| O=C1c2cccc(Sc3ccccc3)c2C(=O)c4c(Sc5ccccc5)cccc14 | 0 |
| CCCOCC(C)OCC(C)O | 0 |
| CCCCC(CC)COP(=S)(S)OCC(CC)CCCC | 0 |
| CCCCC(CC)CN(CC(CC)CCCC)Cn1cncn1 | 0 |
| Oc1ccc2ccccc2c1N=Nc3ccc(cc3Cl)[N+](=O)[O-] | 0 |
| Oc1ccc2ccccc2c1N=Nc3ccc(cc3[N+](=O)[O-])[N+](=O)[O-] | 0 |
| Cc1ccc(N=Nc2c(O)ccc3ccccc23)c(c1)[N+](=O)[O-] | 0 |
| CCCCCl | 0 |
| Clc1ccc(cc1)S(=O)(=O)c2ccc(Cl)cc2 | 0 |
| Cc1cc(ccc1N=C=O)c2ccc(N=C=O)c(C)c2 | 0 |
| O=C=NC1CCC(CC2CCC(CC2)N=C=O)CC1 | 0 |
| CC(C)(OO)c1ccccc1 | 0 |
| NCCCCCCCCCCC(=O)O | 0 |
| OCCCOCCN(CCOCCCO)CCOCCCO | 0 |
| OCCN(CCO)C1CCCCC1 | 0 |
| c1ccc2[nH]nnc2c1 | 0 |
| CC(=O)C(N=Nc1cc(Cl)c(cc1Cl)c2cc(Cl)c(cc2Cl)N=NC(C(=O)C)C(=O)Nc3ccc(C)cc3C)C(=O)Nc4ccc(C)cc4C | 0 |
| CC(=O)C(N=Nc1ccc(cc1Cl)c2ccc(N=NC(C(=O)C)C(=O)Nc3ccccc3)c(Cl)c2)C(=O)Nc4ccccc4 | 0 |
| CC(=O)C(N=Nc1ccc(cc1Cl)c2ccc(N=NC(C(=O)C)C(=O)Nc3ccc(C)cc3C)c(Cl)c2)C(=O)Nc4ccc(C)cc4C | 0 |
| COc1ccccc1NC(=O)C(N=Nc2ccc(cc2Cl)c3ccc(N=NC(C(=O)C)C(=O)Nc4ccccc4OC)c(Cl)c3)C(=O)C | 0 |
| CC(=O)C(N=Nc1ccc(cc1Cl)c2ccc(N=NC(C(=O)C)C(=O)Nc3ccccc3C)c(Cl)c2)C(=O)Nc4ccccc4C | 0 |
| CC(=O)C(N=Nc1ccc(cc1Cl)c2ccc(N=NC(C(=O)C)C(=O)Nc3ccc(C)cc3)c(Cl)c2)C(=O)Nc4ccc(C)cc4 | 0 |
| COc1cc(NC(=O)C(N=Nc2ccc(cc2Cl)c3ccc(N=NC(C(=O)C)C(=O)Nc4cc(OC)c(Cl)cc4OC)c(Cl)c3)C(=O)C)c(OC)cc1Cl | 0 |
| OCCOc1ccc(OCCO)cc1 | 0 |
| CCOc1ccc(NC(=O)C(N=Nc2ccc(cc2Cl)c3ccc(N=NC(C(=O)C)C(=O)Nc4ccc(OCC)cc4)c(Cl)c3)C(=O)C)cc1 | 0 |
| CC(=O)C(N=Nc1ccccc1OCCOc2ccccc2N=NC(C(=O)C)C(=O)Nc3ccc4[nH]c(O)nc4c3)C(=O)Nc5ccc6[nH]c(O)nc6c5 | 0 |
| CC(C)(N=NC(C)(C)C(=N)N)C(=N)N | 0 |
| FC(F)(F)N1C(F)(F)C(F)(F)OC(F)(F)C1(F)F | 0 |
| CCCCCCCCCc1cc(C)cc(Cc2cc(C)cc(CCCCCCCCC)c2O)c1O | 0 |
| NS(=O)(=O)c1ccc(NC(=S)Nc2ccc(F)cc2)cc1 | 0 |
| CC(C)(C1CCCO1)C2CCCO2 | 0 |
| CC(C)C(OC(=O)C(C)C)C(C)(C)COC(=O)C(C)C | 0 |
| CC(=C)CC(C)(C)C | 0 |
| C1CCN2CCCN=C2CC1 | 0 |
| Nc1ccc(N)c(c1)S(=O)(=O)O | 0 |
| CC(C)(C)C1=CC(=Cc2ccccc2)C=C(C1=O)C(C)(C)C | 0 |
| CN(C)Cc1cc(c(O)c(c1)C(C)(C)C)C(C)(C)C | 0 |
| O=C1N(CCc2ccccc2)C(=O)c3ccc4c5ccc6C(=O)N(CCc7ccccc7)C(=O)c8ccc(c9ccc1c3c49)c5c68 | 0 |
| CN1C(=O)c2ccc3c4ccc5C(=O)N(C)C(=O)c6ccc(c7ccc(C1=O)c2c37)c4c56 | 0 |
| O=C1N(C(=O)c2ccc3c4ccc5C(=O)N(C(=O)c6ccc(c7ccc1c2c37)c4c56)c8ccc(cc8)N=Nc9ccccc9)c%10ccc(cc%10)N=Nc%11ccccc%11 | 0 |
| [SH-2] | 0 |
| CN(C)CCO | 0 |
| CC(C)OCCOC(=O)C | 0 |
| CCC(C)NCCO | 0 |
| CC(=O)CC(=O)OCCOC(=O)C(=C)C | 0 |
| CC(=O)C(N=Nc1ccccc1C(=O)O)C(=O)Nc2ccc3[nH]c(O)nc3c2 | 0 |
| C[C](C)C | 0 |
| COc1ccccc1N=NC(C(=O)C)C(=O)Nc2ccc3[nH]c(O)nc3c2 | 0 |
| NC(CO)(CO)CO | 0 |
| CC(=O)C(N=Nc1ccc(Cl)cc1[N+](=O)[O-])C(=O)Nc2ccc3[nH]c(O)nc3c2 | 0 |
| CC(=O)C(N=Nc1ccc(cc1)[N+](=O)[O-])C(=O)Nc2ccc3[nH]c(O)nc3c2 | 0 |
| Cc1ccc(N)c(c1)S(=O)(=O)O | 0 |
| CNC(=O)C(=C1NC(=C2C(=O)NC(=O)NC2=O)c3ccccc13)C#N | 0 |
| FC(F)(F)C(=C)Br | 0 |
| Nc1ccccc1S(=O)(=O)O | 0 |
| OC(=O)COc1ccc(Cl)cc1Cl | 0 |
| CCCCCCCCCCCCCCCCCCO | 0 |
| O=C1N(SC2CCCCC2)C(=O)c3ccccc13 | 0 |
| Clc1ccccn1 | 0 |
| CC1=CCC(CC1)C(C)(C)O | 0 |
| CCCCC(CC)COC(=O)c1ccccc1O | 0 |
| CCCCC(CC)COC(=O)CS | 0 |
| OC(=O)c1cc(ccc1O)[N+](=O)[O-] | 0 |
| CCCCC(C#N)c1ccccc1 | 0 |
| CC(C)(C)O | 0 |
| CC(C)(O)C=C | 0 |
| OC(=O)CCSCCC(=O)O | 0 |
| CC(CCO)CCC=C(C)C | 0 |
| CCC(=CCCC(C)(O)C=C)C | 0 |
| CC(CCOC(=O)C)CCC=C(C)C | 0 |
| CC(=CCCC(=CCO)C)C | 0 |
| CC(=CCCC(=CCOC(=O)C)C)C | 0 |
| CN(C)CCCO | 0 |
| C[N+](C)(C)CCCNC(=O)C=C | 0 |
| CC(=O)Nc1cc(cc(N)c1O)S(=O)(=O)O | 0 |
| CCCCCCCCCC=CCC1CC(=O)OC1=O | 0 |
| CC(C)CC(C)CC(C)C=C(C)C1CC(=O)OC1=O | 0 |
| Cc1ccccc1NC(=O)c2cc3ccccc3c(N=Nc4ccc(cc4C)[N+](=O)[O-])c2O | 0 |
| Cc1ccc(cc1N=Nc2c(O)c(cc3ccccc23)C(=O)Nc4ccccc4)[N+](=O)[O-] | 0 |
| CNS(=O)(=O)c1cc(OC)c(cc1C)N=Nc2c(O)c(cc3ccccc23)C(=O)Nc4ccc5[nH]c(O)nc5c4 | 0 |
| COc1ccc(cc1N=Nc2c(O)c(cc3ccccc23)C(=O)Nc4ccc5[nH]c(O)nc5c4)C(=O)Nc6ccccc6 | 0 |
| COC(C)CCO | 0 |
| COC(C)(C)CCO | 0 |
| COC(C)CCOC(=O)C | 0 |
| CC(=O)C(=CCC1CC=C(C)C1(C)C)C | 0 |
| CC(O)C(C)C=CC1CC=C(C)C1(C)C | 0 |
| CCCCCCC=CC1CC(=O)OC1=O | 0 |
| CC(=O)CC(=O)Nc1ccccc1 | 0 |
| Cc1cccnc1 | 0 |
| CCC(=O)OC1C=CC2C3CCC(C3)C12 | 0 |
| CC(=O)C(N=Nc1ccccc1C(F)(F)F)C(=O)Nc2ccc3[nH]c(O)nc3c2 | 0 |
| O=C1OC(=O)C2CC=CCC12 | 0 |
| CC(CC(c1cc(c(O)cc1C)C(C)(C)C)c2cc(c(O)cc2C)C(C)(C)C)c3cc(c(O)cc3C)C(C)(C)C | 0 |
| FC(F)(F)C(F)(F)F | 0 |
| CC(C)(C)c1cc(Cc2cc(c(O)c(c2)C(C)(C)C)C(C)(C)C)cc(c1O)C(C)(C)C | 0 |
| NNS(=O)(=O)c1ccc(Oc2ccc(cc2)S(=O)(=O)NN)cc1 | 0 |
| Cc1cccc2[nH]nnc12 | 0 |
| Oc1ccc(cc1)S(=O)(=O)c2ccc(O)cc2 | 0 |
| CCCC(c1cc(c(O)cc1C)C(C)(C)C)c2cc(c(O)cc2C)C(C)(C)C | 0 |
| CC(C)(C)CC(C)(C)c1ccc(O)cc1 | 0 |
| COc1cc(OC)c(NC(=O)c2cc3ccccc3c(N=Nc4cc(ccc4OC)C(=O)Nc5ccc(cc5)C(=O)N)c2O)cc1Cl | 0 |
| Oc1nc2cc(NC(=O)c3cc4ccccc4c(N=Nc5cc(Cl)ccc5Cl)c3O)ccc2[nH]1 | 0 |
| CC(C)(c1ccccc1)c2ccc(Nc3ccc(cc3)C(C)(C)c4ccccc4)cc2 | 0 |
| COc1ccccc1NC(=O)c2cc3ccccc3c(N=Nc4ccc(cc4)C(=O)N)c2O | 0 |
| Oc1c(cc2ccccc2c1N=Nc3cc(Cl)ccc3Cl)C(=O)Nc4ccccc4 | 0 |
| Clc1cccc2C(=O)OC(=O)c12 | 0 |
| CCCCCCCc1ccc(O)cc1 | 0 |
| CCOc1ccccc1NC(=O)c2cc3ccccc3c(N=Nc4ccc(cc4)C(=O)N)c2O | 0 |
| CCCCCC(O)C(=CCC)C | 0 |
| Nc1ccc(cc1)S(=O)(=O)O | 0 |
| Cc1ccccc1NC(=O)c2cc3ccccc3c(N=Nc4ccc(Cl)cc4[N+](=O)[O-])c2O | 0 |
| Nc1ccc(O)cc1 | 0 |
| CN1CCOCC1 | 0 |
| CNS(=O)(=O)c1cc(OC)c(cc1OC)N=Nc2c(O)c(cc3ccccc23)C(=O)Nc4ccc5[nH]c(O)nc5c4 | 0 |
| O=C1NC(=O)C(=C2NC(=C3C(=O)NC(=O)NC3=O)c4ccccc24)C(=O)N1 | 0 |
| CC1=CC2=NC(=O)NC2=CC1=NNC3C(=O)NC(=O)NC3=O | 0 |
| C=CC1CC2CC1C=C2 | 0 |
| CC(CCCC(C)(C)O)CC=O | 0 |
| Clc1ccc2C(=O)OC(=O)c2c1 | 0 |
| CC(C)CCCCCOC(=O)CS | 0 |
| O=C1c2ccccc2C(=O)c3ccccc13 | 0 |
| CCc1ccc(C)nc1 | 0 |
| OCCOCC(COCCO)OCCO | 0 |
| CCC(C)CCCOC(=O)CCc1cc(c(O)c(c1)C(C)(C)C)n2nc3ccccc3n2 | 0 |
| CCCCCCCCCCCCOC(=O)c1ccccc1 | 0 |
| C[N+](C)(CCOC(=O)C=C)Cc1ccccc1 | 0 |
| CCC=C | 0 |
| CCCCCCCCCCCCCCCCCCCN | 0 |
| CCCCCCCCCCCCCCCCN | 0 |
| OCCCOCC(OCCCO)C(OCCCO)C(OCCCO)C(COCCCO)OCCCO | 0 |
| CCCCC(CC)COC(=O)CCCCCCCCC(=O)OCC(CC)CCCC | 0 |
| CC(C)c1ccccc1C(C)C | 0 |
| BrCBr | 0 |
| CCCCOC(=O)CCCCCCCCC(=O)OCCCC | 0 |
| Nc1ccc(cc1N)S(=O)(=O)O | 0 |
| C[N+](C)(CC=C)CC=C | 0 |
| CC(C)(COCCCOC(=O)C=C)COCCCOC(=O)C=C | 0 |
| OC(=O)C(Cl)(Cl)Cl | 0 |
| CNCCS(=O)(=O)O | 0 |
| CCOC(=O)CC(=O)C | 0 |
| CCCCCCCCC(CCCCCCCCC(=O)O)C(CCCCCCCC(=O)O)CCC=CCCCCC | 0 |
| OC(=O)CC=CC=CC=CC=CC=CC=CC=CC=COC(=O)CC=CC=CC=CC=CC=CC=CC=CC=COC(=O)CC=CC=CC=CC=CC=CC=CC=CC=C | 0 |
| CCCCC(CC)C(=O)OOC(C)(C)C | 0 |
| CC(CC(=O)OOC(C)(C)C)CC(C)(C)C | 0 |
| CN(C)CCCNCCCN(C)C | 0 |
| S=C(SN1CCOCC1)N2CCOCC2 | 0 |
| CCCCCCC(O)CCCCCCCCCCC(=O)NCCNC(=O)CCCCCCCCCCC(O)CCCCCC | 0 |
| CC[N+](CC)(CC)CC | 0 |
| CN(C)CCCN(C)C | 0 |
| C[N+](C)(C)C | 0 |
| O=C(NCCCCCCNC(=O)N1CCCCCC1=O)N2CCCCCC2=O | 0 |
| CCCCCCCCCCCCCCCCCCCCCC[N+](C)(C)C | 0 |
| CCN(CC)CCCN | 0 |
| CCCCN(CCCC)CCCC | 0 |
| CCCCCCCCCCCCN(C)C | 0 |
| CCCCCCCCCCCCCCCCN(C)C | 0 |
| CCCCCCCCCCN(C)C | 0 |
| CN(C)C(=O)C=C | 0 |
| CN(C)CCCN | 0 |
| CC(C)CCC(C)Nc1ccc(Nc2ccccc2)cc1 | 0 |
| CCCCCCCCCCCCCCCCCCN(C)C | 0 |
| CCCCCCCCCCCCCCN(C)C | 0 |
| CC(C)CC(C)Nc1ccc(Nc2ccccc2)cc1 | 0 |
| CC(=O)Nc1ccc(NC(=O)c2cc3ccccc3c(N=Nc4cc(ccc4Cl)C(=O)N)c2O)cc1 | 0 |
| CC(=O)C(N=Nc1ccc(cc1)C(=O)Nc2ccc(cc2)C(=O)N)C(=O)Nc3ccc4[nH]c(O)nc4c3 | 0 |
| COc1cc(NC(=O)c2cc3ccccc3c(N=Nc4cc(ccc4OC)C(=O)Nc5ccccc5)c2O)c(OC)cc1Cl | 0 |
| CCN(CC)S(=O)(=O)c1ccc(OC)c(c1)N=Nc2c(O)c(cc3ccccc23)C(=O)Nc4cc(Cl)c(OC)cc4OC | 0 |
| COc1ccc(Cl)cc1NC(=O)c2cc3ccccc3c(N=Nc4cc(ccc4OC)C(=O)Nc5ccccc5)c2O | 0 |
| CC(=C)C(=O)NCO | 0 |
| CCN(CC)CCN | 0 |
| COc1ccc(cc1N=Nc2c(O)c(cc3ccccc23)C(=O)Nc4cc(Cl)ccc4C)C(=O)Nc5ccccc5 | 0 |
| CCCCCCCCCCCCCCCCCC(=O)NCCCN(C)C | 0 |
| CC(C)Nc1ccc(Nc2ccccc2)cc1 | 0 |
| CCCCNS(=O)(=O)c1ccccc1 | 0 |
| CCNCC | 0 |
| CNC(=S)NN | 0 |
| CCCCCCCCCCCCCCCCCCCCC=CCC=CCCCCCCCCCCCNCCCCCCCCCCCCCCCCCCCCC=CCC=CCCCCCCCCCCC | 0 |
| OCCCOCC(COCCCO)(COCCCO)COCCCO | 0 |
| CCCCC(CC)CCCCCC(C)CCCC(=O)O | 0 |
| Nc1nc(Cl)nc(Nc2cc(cc3cc(c(N=Nc4cc(ccc4O)[N+](=O)[O-])c(O)c23)S(=O)(=O)O)S(=O)(=O)O)n1 | 0 |
| Cc1ccc(cc1)[N+](=O)[O-] | 0 |
| CCCCO[P+](=O)OCCCC | 0 |
| CCC(C)OP(=S)(S)OC(C)CC(C)C | 0 |
| CC1CCC2C(C1)C(=O)OC2=O | 0 |
| CCC(C)(OO)OOC(C)(CC)OO | 0 |
| CCCCCCCCC=CCCCCCCCCO | 0 |
| CCCCCCCCC=CCCCCCCCC(=O)N(C)CC(=O)O | 0 |
| CC(C)CP(=S)(S)CC(C)C | 0 |
| OCC1OC(OC2(CO)OC(CO)C(O)C2O)C(O)C(O)C1O | 0 |
| C[N+](C)(C)CCOC(=O)C=C | 0 |
| CC(=C)C(=O)OCC[N+](C)(C)C | 0 |
| Cc1cc(c(O)c(C)c1CN2C(=O)N(Cc3c(C)cc(c(O)c3C)C(C)(C)C)C(=O)N(Cc4c(C)cc(c(O)c4C)C(C)(C)C)C2=O)C(C)(C)C | 0 |
| CC(c1ccccc1)c2cc(C(C)c3ccccc3)c(O)c(c2)C(=O)O | 0 |
| CC(C)(C)CSSCC(C)(C)C | 0 |
| CCCCC(CC)COP(=S)(S)OCC(C)C | 0 |
| CC=C=C | 0 |
| Cc1cc(C)cc(c1)N2C(=O)c3ccc4c5ccc6C(=O)N(C(=O)c7ccc(c8ccc(C2=O)c3c48)c5c67)c9cc(C)cc(C)c9 | 0 |
| CCCCN(CCCC)C(=S)S | 0 |
| CCCCCC(=O)OCC=C | 0 |
| OCC=C | 0 |
| C=CC=C | 0 |
| CCCCOC(=O)c1ccccc1N=Nc2c(O)c(cc3ccccc23)C(=O)Nc4ccc5[nH]c(O)nc5c4 | 0 |
| OCC1CCC(CO)CC1 | 0 |
| CC(C)CCCCCOC(=O)CCCCC(=O)OCCCCCC(C)C | 0 |
| OS(=O)(=O)OOS(=O)(=O)O | 0 |
| CCOC(=O)C1CCC(CC1)C(=O)OCC | 0 |
| CCCCCCOC(=O)CCCCC(=O)OCCCCCC | 0 |
| COC(=O)C1CCC(CC1)C(=O)OC | 0 |
| CC(C)OC(=O)CCCCCCCCC(=O)OC(C)C | 0 |
| COC(=O)C=CC(=O)OC | 0 |
| CO[P+](=O)OC | 0 |
| COC(=O)c1ccc(C(=O)OC)c(c1)N=NC(C(=O)C)C(=O)Nc2ccc3[nH]c(O)nc3c2 | 0 |
| CS(=O)C | 0 |
| COC(=O)c1cc(cc(c1)C(=O)OC)N=NC(C(=O)C)C(=O)Nc2ccc3[nH]c(O)nc3c2 | 0 |
| COP(=O)(CCC(=O)NCO)OC | 0 |
| OS(=O)(=O)OS(=O)(=O)O | 0 |
| CCOC(=O)C1OC1(C)c2ccccc2 | 0 |
| C=C | 0 |
| O=C1OC(=O)C2CCCCC12 | 0 |
| CCCCCCCCCCCCN | 0 |
| OCCCCCCO | 0 |
| CCCCCCCCCCCCCCCCCCC=C | 0 |
| S=C(SSSSSSC(=S)N1CCCCC1)N2CCCCC2 | 0 |
| NN | 0 |
| O=C1OC(=O)c2ccc3c4ccc5C(=O)OC(=O)c6ccc(c7ccc1c2c37)c4c56 | 0 |
| CCCCCCOC1OC(CO)C(O)C(O)C1O | 0 |
| CC(=C)C=C | 0 |
| CC(=C)OC(=O)C | 0 |
| O=C1NC(=O)c2ccc3c4ccc5C(=O)NC(=O)c6ccc(c7ccc1c2c37)c4c56 | 0 |
| OC(=O)C(=O)O | 0 |
| COC(=O)CCc1cc(c(O)c(c1)C(C)(C)C)C(C)(C)C | 0 |
| COC(=O)c1ccc(cc1N=Nc2c(O)c(cc3ccccc23)C(=O)Nc4ccccc4OC)C(=O)Nc5cc(Cl)ccc5Cl | 0 |
| CC(=C)C(=O)N | 0 |
| COC(=O)CCCCCCCCC=C | 0 |
| C1COCCN1 | 0 |
| CS(=O)(=O)O | 0 |
| Cc1ccccc1Cc2ccccc2 | 0 |
| O=CN1CCOCC1 | 0 |
| CCCCC=CCC | 0 |
| CCCCCCCCCCCCCCCCCCN | 0 |
| CCCCCCCCP(=O)(O)O | 0 |
| OCCCCCO | 0 |
| CCCC(=O)C | 0 |
| c1ccncc1 | 0 |
| CC=C | 0 |
| CC(=C)CS(=O)(=O)O | 0 |
| COC(=O)c1cc(cc(c1)S(=O)(=O)O)C(=O)OC | 0 |
| CCCCCCCCCCCC(=O)OCCS(=O)(=O)O | 0 |
| OC(=O)C1CCC(=O)N1 | 0 |
| CC(=O)OC(C)(C)C | 0 |
| CC(C)(C)OOC(=O)c1ccccc1 | 0 |
| Oc1cc(N=Nc2ccc3c(cccc3c2)S(=O)(=O)O)c(c4ccc(NC(=O)Nc5ccc6c(O)c(N=Nc7ccc8cccc(c8c7)S(=O)(=O)O)c(cc6c5)S(=O)(=O)O)cc14)S(=O)(=O)O | 0 |
| CCCCCCCCCCCCC=C | 0 |
| CC1C=CC=C1 | 0 |
| CCOP(OCC)OCC | 0 |
| CC(C)CCCCCCCOC(=O)c1ccc(C(=O)OCCCCCCCC(C)C)c(c1)C(=O)OCCCCCCCC(C)C | 0 |
| Cc1cc(N=Nc2cc(ccc2S(=O)(=O)O)S(=O)(=O)O)c(C)cc1N=Nc3ccc(Nc4nc(N)nc(Cl)n4)c5c(cccc35)S(=O)(=O)O | 0 |
| CC(C)CC(C)OP(=S)(S)OC(C)CC(C)C | 0 |
| OC(=O)CCCCCCCCC=C | 0 |
| CC(C)CCCCCOP(=S)(S)OCCCCCC(C)C | 0 |
| NCCC(=O)O | 0 |
| CC1(C)OCC(CO)O1 | 0 |
| CCCCCCCCC=CCCCCCCCC#N | 0 |
| CCC1(COC(=O)C=C)COCOC1 | 0 |
| CCCCCCCCCCCCCCC=C1OC(=O)C1CCCCCCCCCCCCCC | 0 |
| CCOCOC1CCCCCCCCCCC1 | 0 |
| CCC=CCCO | 0 |
| CCCCCCCCCCCCCCCCOC(=O)OOC(=O)OCCCCCCCCCCCCCCCC | 0 |
| CCC(O)OCC(C)OC(O)CC | 0 |
| CC(C)(CC(=C)c1ccccc1)c2ccccc2 | 0 |
| CCCCCCCCCCCCCCOC(=O)OOC(=O)OCCCCCCCCCCCCCC | 0 |
| CC(C)(c1ccccc1)C(C)(C)c2ccccc2 | 0 |
| CC(O)CN(CC(C)O)c1ccc(C)cc1 | 0 |
| CC(O)CNCC(C)O | 0 |
| O=C1C=CC(=O)N1c2ccc(Cc3ccc(cc3)N4C(=O)C=CC4=O)cc2 | 0 |
| CC(C)CCOCCC(C)C | 0 |
| CC(O)CN(CCCN(C)C)CC(C)O | 0 |
| FC(=C(F)OC(F)(F)F)F | 0 |
| CCCCC(CC)C(=O)OOC(C)(C)CC(C)(C)C | 0 |
| ClC1=C(Cl)C2(Cl)C3CCC4C(CCC3C1(Cl)C2(Cl)Cl)C5(Cl)C(=C(Cl)C4(Cl)C5(Cl)Cl)Cl | 0 |
| CC(C)(C)OOC1(CCCCC1)OOC(C)(C)C | 0 |
| CC(COC(=O)c1ccccc1)OC(=O)c2ccccc2 | 0 |
| Cc1nccn1C | 0 |
| CN1CN(C)CN(C)C1 | 0 |
| CN(C)CCCN(CCCN(C)C)CCCN(C)C | 0 |
| CC(C)CCCCCCCCCCCCCCC(=O)OC(CO)CO | 0 |
| OCCN1C(=O)N(CCO)C(=O)N(CCO)C1=O | 0 |
| O=C=NCc1cccc(CN=C=O)c1 | 0 |
| O=C1OC=CO1 | 0 |
| CCCCCCCCCCCCCCCC(=O)OCC(O)C1OCC(O)C1O | 0 |
| C1CN2CCN1CC2 | 0 |
| CCCCCCCCCCCC(=O)OCC(O)C1OCC(O)C1O | 0 |
| CCc1ccc(CC)cc1 | 0 |
| CCCCCCCCC=CCCCCCCCC(=O)OCC(OC(=O)CCCCCCCC=CCCCCCCCC)C1OCC(O)C1OC(=O)CCCCCCCC=CCCCCCCCC | 0 |
| CN1CCN(C)CC1 | 0 |
| OCCN1CCNC1=O | 0 |
| O=C=NCCCCCCN=C=O | 0 |
| CC1(C)CC(O)CC(C)(C)N1CCO | 0 |
| OCCN1CCCC1=O | 0 |
| CC(=O)c1ccc(O)cc1 | 0 |
| ClCCCN1CCN(CC1)c2cccc(Cl)c2 | 0 |
| CC(=O)c1ccc2ccccc2c1 | 0 |
| CC(=O)c1ccc(C)cc1 | 0 |
| CC1CC(NC(=O)N)NC(=O)N1 | 0 |
| CCCCCCCCCCCCCCCCCCOC=C | 0 |
| CC(O)CN(CCCN(C)C)CCCN(C)C | 0 |
| NCc1ccccc1 | 0 |
| CC(O)CN(C)C | 0 |
| ClCc1ccccc1Cl | 0 |
| CC(O)CN | 0 |
| CC(=O)OC(C)(C)C1CCC(=CC1)C | 0 |
| CCN1CCNCC1 | 0 |
| OC1(CCCCC1)C#C | 0 |
| CCCCCCCCCCCCCCCC(=O)OC1CC(N(C1)C(=O)CCCCCCCCCCCCCCC)C(=O)O | 0 |
| CN1CCNCC1 | 0 |
| Cn1ccnc1 | 0 |
| NCCCCCCCCCCCC(=O)O | 0 |
| CCC[N+](=O)[O-] | 0 |
| CC(=O)c1ccccc1 | 0 |
| O=C1C=CC(=O)N1c2ccccc2 | 0 |
| CC(C)CCCCCCCCCCCCCCCOC(=O)CCCCCCCCCCCCCCC(C)C | 0 |
| CC(C)CCCCCCCCCCCCCCC(=O)O | 0 |
| FC(F)(F)C1(F)OC1(F)F | 0 |
| CC1(C)CC(O)CC(C)(C)N1 | 0 |
| CCC(C)(N=NC(C)(CC)C#N)C#N | 0 |
| CC(C)(C)OOC(C)(C)C | 0 |
| CC1(C)COP(=S)(OP2(=S)OCC(C)(C)CO2)OC1 | 0 |
| OCC(CO)(CO)CO | 0 |
| CC(C)(N=NC(C)(C)C#N)C#N | 0 |
| OCC(CBr)(CBr)CBr | 0 |
| Cc1ccc(C)c(O)c1C | 0 |
| CCC(COC(=O)C=C)(COC(=O)C=C)COC(=O)C=C | 0 |
| CCCCCCCCC(=O)OCC(CC)(COC(=O)CCCCCCCC)COC(=O)CCCCCCCC | 0 |
| CC(C)(CO)CO | 0 |
| CN(C)Cc1cc(CN(C)C)c(O)c(CN(C)C)c1 | 0 |
| CC(C)CCC(C)(O)C#CC(C)(O)CCC(C)C | 0 |
| CCCCC(CC)C(=O)OOC(C)(C)CCC(C)(C)OOC(=O)C(CC)CCCC | 0 |
| CC1=CC2C(C1)C3CC2C(=C3)C | 0 |
| CC(C)CCCC(C)CCCC(C)CCCCC(C)CCCC(C)CCCC(C)C | 0 |
| CC(C)CC(C)CC(O)CC(C)C | 0 |
| CCCCOCCOCCOC(=O)C(=C)C | 0 |
| CN(C)CCOCCO | 0 |
| CC(C)(C)CC(C)(C)c1ccc(O)c(c1)n2nc3ccccc3n2 | 0 |
| CCC(C)(C)c1cc(c(O)c(c1)C(C)(C)CC)n2nc3ccccc3n2 | 0 |
| Cc1ccc(O)c(c1)n2nc3ccccc3n2 | 0 |
| OCCOCCOc1ccccc1 | 0 |
| NCCOCCO | 0 |
| CCCCCCCCCc1ccc(OCC(=O)O)cc1 | 0 |
| CCNCCO | 0 |
| CCN(CC)CCOC(=O)C(=C)C | 0 |
| CCCCNCCO | 0 |
| CNCCO | 0 |
| CCCCN(CCO)CCCC | 0 |
| CCCCCCCC(=O)NCC(=O)O | 0 |
| CCC(N)(CO)CO | 0 |
| CC(=C)C(=O)OCCNC(C)(C)C | 0 |
| CCCCCCCCCCCCCCCCCCOC(=O)C=CC(=O)OCCCCCCCCCCCCCCCCCC | 0 |
| CCCCCCCCCCCCCCCCCOC(=O)C=CC(=O)OCCCCCCCCCCCCCCCCC | 0 |
| CCCCCCCCCCCCCCCOC(=O)C=CC(=O)OCCCCCCCCCCCCCCC | 0 |
| CCCCCCCCOC(=O)C=CC(=O)OCCCCCCCC | 0 |
| Cc1cc(NC(=O)c2cc3ccccc3c(N=Nc4cc(Cl)ccc4C)c2O)c(C)cc1NC(=O)c5cc6ccccc6c(N=Nc7cc(Cl)ccc7C)c5O | 0 |
| CCCC(=NO)C | 0 |
| C=CC(=O)OC1C=CC2C3CCC(C3)C12 | 0 |
| COc1ccccc1NC(=O)C(N=Nc2ccc(Cl)cc2[N+](=O)[O-])C(=O)C | 0 |
| CC(=O)C(N=Nc1ccc(Cl)cc1[N+](=O)[O-])C(=O)Nc2ccccc2Cl | 0 |
| COc1ccc(N=NC(C(=O)C)C(=O)Nc2ccccc2OC)c(c1)[N+](=O)[O-] | 0 |
| OS(=O)(=O)c1ccccc1N=C2C=CC(=C(c3ccc(Nc4ccccc4)cc3)c5ccc(Nc6ccccc6)cc5)C=C2 | 0 |
| CC(=O)C(N=Nc1ccc(C)cc1[N+](=O)[O-])C(=O)Nc2ccccc2 | 0 |
| N#CCN(CC#N)CC#N | 0 |
| CCOCCOCCOCCOC(=O)C(=C)C | 0 |
| CC(C)(c1ccc(OCCO)cc1)c2ccc(OCCO)cc2 | 0 |
| NC(CO)CO | 0 |
| Cc1ccc(cc1N=Nc2c(O)c(cc3ccccc23)C(=O)Nc4cc(C)c(NC(=O)c5cc6ccccc6c(N=Nc7c(C)cccc7C(=O)OCCCl)c5O)cc4C)C(=O)OCCCl | 0 |
| CCC(CO)(CO)CO | 0 |
| CCc1ccc2C(=O)c3ccccc3C(=O)c2c1 | 0 |
| CCCCC(CC)CNCC(CC)CCCC | 0 |
| CCCCC(CC)CN | 0 |
| CC(C)COC=C | 0 |
| CCC(C)(C)O | 0 |
| CC=C(C)C | 0 |
| Cc1ccc(cc1S(=O)(=O)O)[N+](=O)[O-] | 0 |
| OC(=O)CC(=C)C(=O)O | 0 |
| CC(CN)CCCN | 0 |
| CCC(=C)C | 0 |
| CCC(C)CO | 0 |
| Nc1ccccc1[N+](=O)[O-] | 0 |
| CCCCCCCCCCCCCC(=O)OCC(CCCCCCCC)CCCCCCCCCC | 0 |
| C=CC(=O)OCCOc1ccccc1 | 0 |
| CC(=C)C | 0 |
| CC(C)(C)S | 0 |
| CCCCCC(CO)CCC | 0 |
| CC(=O)OC1CCCCC1C(C)(C)C | 0 |
| CC(COCC(C)OC(=O)C=C)OCC(C)OC(=O)C=C | 0 |
| CN(C)CCCN1CN(CCCN(C)C)CN(CCCN(C)C)C1 | 0 |
| CC12CCC3C(=CC=C4CC5(CCC34C)OCCO5)C1CCC2C6(C)OCCO6 | 0 |
| CC(=O)C(N=Nc1cc(ccc1Cl)C(=O)Nc2cc(Cl)ccc2C)C(=O)Nc3cc(Cl)c(NC(=O)C(N=Nc4cc(ccc4Cl)C(=O)Nc5cc(Cl)ccc5C)C(=O)C)cc3Cl | 0 |
| Cc1cc(C)c(O)c(c1)C(C)(C)C | 0 |
| CC(=O)C(N=Nc1cc(ccc1Cl)C(=O)Nc2cc(Cl)ccc2C)C(=O)Nc3cc(C)c(NC(=O)C(N=Nc4cc(ccc4Cl)C(=O)Nc5cc(Cl)ccc5C)C(=O)C)cc3C | 0 |
| NCCCOCCCCOCCCN | 0 |
| NCCCOCCOCCOCCCN | 0 |
| CC(=O)C(N=Nc1cc(ccc1Cl)C(=O)Nc2cc(ccc2Oc3ccc(Cl)cc3)C(F)(F)F)C(=O)Nc4cc(Cl)c(NC(=O)C(N=Nc5cc(ccc5Cl)C(=O)Nc6cc(ccc6Oc7ccc(Cl)cc7)C(F)(F)F)C(=O)C)cc4C | 0 |
| CC1CC(CC(C)(C)C1)OC(=O)c2ccccc2O | 0 |
| CC(=O)C(N=Nc1cc(ccc1Cl)C(=O)Nc2cccc(Cl)c2C)C(=O)Nc3cc(Cl)c(NC(=O)C(N=Nc4cc(ccc4Cl)C(=O)Nc5cccc(Cl)c5C)C(=O)C)cc3C | 0 |
| Cc1ccc(C=O)cc1C | 0 |
| CCN(CC)c1ccc(C=C(C#N)C(=O)OCCC[N+](C)(C)CCOC(=O)C(=Cc2ccc(cc2)N(CC)CC)C#N)cc1 | 0 |
| CC(CCOC(=O)C)CC(C)(C)C | 0 |
| COC(C)(C)CCOC(=O)C | 0 |
| CCCCC(CC)C(=O)OCC(COC(=O)C(CC)CCCC)(COC(=O)C(CC)CCCC)COC(=O)C(CC)CCCC | 0 |
| SCCC(=O)OCC(COC(=O)CCS)(COC(=O)CCS)COC(=O)CCS | 0 |
| CC(C)C(O)C(C)(C)COC(=O)C(C)C | 0 |
| Cc1nn2c(O)c3ccccc3nc2c1N=Nc4ccc(Cl)cc4[N+](=O)[O-] | 0 |
| CC(C)(CO)COC(=O)C(C)(C)CO | 0 |
| CC(CO)(CO)C=O | 0 |
| C[n+]1ccn(C=C)c1 | 0 |
| COCCCN | 0 |
| CC(C)CCO | 0 |
| CC(C)CCOC(=O)C | 0 |
| OS(=O)(=O)CCC[n+]1ccccc1 | 0 |
| Cc1cccc(N)c1 | 0 |
| CC1(C)CCCC2(C)C1CCC3(C)OCCC23 | 0 |
| OC(=O)CCS | 0 |
| Brc1cc2C(=O)c3cccc4c(Br)cc5C(=O)c6cccc1c6c2c5c34 | 0 |
| Cc1nn(c(O)c1N=Nc2ccc(cc2Cl)c3ccc(N=Nc4c(C)nn(c4O)c5ccccc5)c(Cl)c3)c6ccccc6 | 0 |
| CC1=NN(C(=O)C1N=Nc2ccc(cc2Cl)c3ccc(N=NC4C(=NN(C4=O)c5ccc(C)cc5)C)c(Cl)c3)c6ccc(C)cc6 | 0 |
| C(CN1CCOCC1)OCCN2CCOCC2 | 0 |
| Nc1ccc(Cc2ccc(N)c(Cl)c2)cc1Cl | 0 |
| Nc1ccc(c2ccc(N)c3C(=O)c4ccccc4C(=O)c23)c5C(=O)c6ccccc6C(=O)c15 | 0 |
| CC(C)(C)CC(C)(C)c1ccc(Nc2ccc(cc2)C(C)(C)CC(C)(C)C)cc1 | 0 |
| Clc1c(Cl)c(Cl)c2C(=O)C(C(=O)c2c1Cl)c3ccc4cccc(N5C(=O)c6c(Cl)c(Cl)c(Cl)c(Cl)c6C5=O)c4n3 | 0 |
| CC(C)(c1ccccc1)c2ccc(O)cc2 | 0 |
| NC1CCC(CC2CCC(N)CC2)CC1 | 0 |
| OCCCCOC=C | 0 |
| Cc1cc(ccc1O)C2(CCCCC2)c3ccc(O)c(C)c3 | 0 |
| CC(C)(C1CCC(O)CC1)C2CCC(O)CC2 | 0 |
| C[N+](=CCC(O)CC(=O)N)C | 0 |
| CCCCOC(=O)C=CC(=O)O | 0 |
| [O-][N+](=O)c1cc(ccc1Nc2ccccc2)S(=O)(=O)Nc3ccccc3 | 0 |
| Nc1ccc(F)cc1 | 0 |
| CC(=O)Nc1ccc(cc1)S(=O)(=O)Cl | 0 |
| CC(=O)CC(C)(C)O | 0 |
| CC1C=CCC2C1C(=O)OC2=O | 0 |
| COc1ccc(O)cc1 | 0 |
| Cc1ccc(cc1)S(=O)(=O)Cl | 0 |
| CC(=C1CCC(=CC1)C)C | 0 |
| CC(=CC(=O)C)C | 0 |
| CC(=O)CCc1ccccc1 | 0 |
| Oc1c2ccccc2c(O)c3c1ccc4nc5c6C(=O)c7ccccc7C(=O)c6ccc5nc34 | 0 |
| CC1CCC2=C(C1)C(=O)OC2=O | 0 |
| CN(CCCCCC(=O)O)S(=O)(=O)c1ccccc1 | 0 |
| Cc1ccc(cc1)S(=O)(=O)NCCCCCC(=O)O | 0 |
| CCCCCCCCC=CCCCCCCCC(=O)OCCCCCCCC(C)C | 0 |
| CC(COCCCO)OP(OCCOCCO)OCCOC(C)(C)C(C)(C)O | 0 |
| CCCCCCCCCCCCCCO | 0 |
| CCCCCCCCCCCCO | 0 |
| CCCCCCCC=CCC | 0 |
| COC(=O)c1c(Cl)c(Cl)c(Cl)c(Cl)c1C#N | 0 |
| N(c1ccccc1)c2ccccc2 | 0 |
| OCCCCO | 0 |
| CC(C)CCCCCCCOC(=O)CCCCCCCC(=O)OCCCCCCCC(C)C | 0 |
| CCOC(=O)C(C)CC | 0 |
| OC(=O)CN(CCN(CC(=O)O)CC(=O)O)CC(=O)O | 0 |
| CC(C)C1CCC(C)CC1 | 0 |
| C1CCC=CC1 | 0 |
| CCCCCCCCCCCCOCCO | 0 |
| CCCCCCOP(=O)(O)O | 0 |
| CCCCCCCCCCCCc1ccccc1S(=O)(=O)O | 0 |
| OC(=O)C=CC=CC=CC=CC=CC=CC=C | 0 |
| CCCCCCCCCCCCCCCCC(=O)O | 0 |
| CCCCCCCCCCCCCCCCCC(=O)OCCCCCCCCCCCCCC | 0 |
| CCCCCCCCCCCCCCCCCCC(=O)O | 0 |
| CCCCCCCCC(=O)O | 0 |
| CC(C)CCCCCCCCCCCCCCCO | 0 |
| CCCCCCCCCCCCCCCCCC(=O)OC(CO)COC(=O)CCCCCCCCCCCCCCC | 0 |
| CCCCCCCCCCCCC(=O)OCC(O)CO | 0 |
| CCCCCCCO | 0 |
| CC1CC(CC2CCC(N)C(C)C2)CCC1N | 0 |
| CC(C)(C)OO | 0 |
| CC(C)CCCC=C | 0 |
| CC(C)CCCCCCCCCCO | 0 |
| Cc1cc(c(cc1Cl)N=Nc2c(O)c(cc3ccccc23)C(=O)O)S(=O)(=O)O | 0 |
| CCCCCCCCC=CCCCCCCCC(=O)OC(O)CCOCCC(O)O | 0 |
| CN(C)CCCNCCCN | 0 |
| CC(C)C(NC(=O)N)NC(=O)N | 0 |
| Oc1c(cc2ccccc2c1N=Nc3cccc(Cl)c3Cl)C(=O)Nc4cccc5c(NC(=O)c6cc7ccccc7c(N=Nc8cccc(Cl)c8Cl)c6O)cccc45 | 0 |
| Oc1c(cc2ccccc2c1N=Nc3cc(Cl)ccc3Cl)C(=O)Nc4cc(Cl)c(NC(=O)c5cc6ccccc6c(N=Nc7cc(Cl)ccc7Cl)c5O)cc4Cl | 0 |
| Oc1c(cc2ccccc2c1N=Nc3cc(ccc3Cl)C(F)(F)F)C(=O)Nc4cc(Cl)c(NC(=O)c5cc6ccccc6c(N=Nc7cc(ccc7Cl)C(F)(F)F)c5O)cc4Cl | 0 |
| Oc1c(cc2ccccc2c1N=Nc3cc(Cl)ccc3Cl)C(=O)Nc4ccc(NC(=O)c5cc6ccccc6c(N=Nc7cc(Cl)ccc7Cl)c5O)c(Cl)c4 | 0 |
| CN(C)CCCCCCN(C)C | 0 |
| Oc1cc2ccccc2cc1C(=O)N=NNc3cc(Cl)c(cc3Cl)c4ccc(cc4)c5cc(Cl)c(NN=NC(=O)c6cc7ccccc7cc6O)cc5Cl | 0 |
| CCN(C)C | 0 |
| CN(C)C(=O)CC(=O)C | 0 |
| CCN(CC)CC | 0 |
| CN(C)C1CCCCC1 | 0 |
| CCCN(C)C | 0 |
| CCCN(CCC)CCC | 0 |
| CC(C)(C)CC(C)(C)NC(=O)C=C | 0 |
| CC(C)(C)N(Sc1nc2ccccc2s1)Sc3nc4ccccc4s3 | 0 |
| CCCCC(CC)CNC1(C=CCc2ccccc12)N=Nc3ccc(cc3C)N=Nc4ccccc4C | 0 |
| CC(C)(C)NSc1nc2ccccc2s1 | 0 |
| CCCCCCCCC=CCCCCCCCC(=O)NCC(C)O | 0 |
| CN(CCCN)CCCN | 0 |
| NCCCNCCCN | 0 |
| COc1cc(NS(=O)(=O)c2ccc(C)cc2)c3C(=O)c4ccccc4C(=O)c3c1N | 0 |
| CC(C)(C)CC(C)(C)N(c1ccccc1)c2cccc3ccccc23 | 0 |
| COc1cc(NC(=O)C(N=Nc2cc(OC)c(cc2OC)S(=O)(=O)Nc3ccccc3)C(=O)C)c(OC)cc1Cl | 0 |
| CN(C)CCN(C)CCN(C)C | 0 |
| COc1ccc(Cl)cc1NC(=O)C(N=Nc2ccc(cc2OC)[N+](=O)[O-])C(=O)C | 0 |
| CN(C)CCCNC(=O)C(=C)C | 0 |
| CCCCCCCCCCCC(=O)NCCC[N+](C)(C)CC(O)CS(=O)(=O)O | 0 |
| CCN(CCOC(C)OCC(C)C)c1ccc(cc1)N=Nc2ccccc2 | 0 |
| CCCCNCCCC | 0 |
| CCCNCC | 0 |
| CC(C)NC(=O)C(=C)C | 0 |
| NCCCNCCNCCCN | 0 |
| CCCCCCCCCCCCCCCCC#N | 0 |
| CCCCCCCCCC=C | 0 |
| CC=CCCC=CCCCCC=CCCC=CCCC=CCCC=CC | 0 |
| CC=CC=CC | 0 |
| CCC=CCC | 0 |
| Cc1ccccc1OP(=S)(S)Oc2ccccc2C | 0 |
| OC1CCCCCCCCCCC1 | 0 |
| CCNC(=S)OC(C)C | 0 |
| NC(=O)N | 0 |
| OP(O)O | 0 |
| CC(C)CC(C)OP(=S)(S)OC(C)C | 0 |
| C=CC(=O)OCCCOCC(COCCCOC(=O)C=C)OCCCOC(=O)C=C | 0 |
| CC(C)COP(=S)(S)OCC(C)C | 0 |
| CC(O)COC(C)CO | 0 |
| CCCCCOC(=O)CC | 0 |
| CCC(COCCOC(=O)C=C)(COCCOC(=O)C=C)COCCOC(=O)C=C | 0 |
| CC(=CCCC(=CCCC(C)(O)C=C)C)C | 0 |
| CC(C)(C)CCCCCC(=O)OOC(C)(C)C | 0 |
| CCOP(=S)(S)OCC | 0 |
| CCCCCCCCCCCCCCCCCC(=O)OCC(O)C1OCC(O)C1O | 0 |
| CCCCCCCCCCCCCCCCCC(=O)OCC(OC(=O)CCCCCCCCCCCCCCCCC)C1OCC(O)C1OC(=O)CCCCCCCCCCCCCCCCC | 0 |
| CCCCCCCCCCCC(=O)OC=C | 0 |
| CCC=CCCOC(=O)C | 0 |
| CCC(COCC(CC)(COC(=O)C=C)COC(=O)C=C)(COC(=O)C=C)COC(=O)C=C | 0 |
| CC(=O)CC(=O)OCC=C | 0 |
| CCC(C)O | 0 |
| CC(C)O | 0 |
| NC(=N)N | 0 |
| NC(=N)S(=O)O | 0 |
| C1CCCNCC1 | 0 |
| CC(CC(C)(C)C)CP(=O)(O)CC(C)CC(C)(C)C | 0 |
| OC(=O)CCCCCCCC(=O)O | 0 |
| CCCCOCCOC(=O)CCCCC(=O)OCCOCCCC | 0 |
| CCCCC(CC)COC(=O)C=CC(=O)OCC(CC)CCCC | 0 |
| CCCCC(CC)COC(=O)CCCCCCCC(=O)OCC(CC)CCCC | 0 |
| CC(C)COC(=O)C1CCCCC1C(=O)OCC(C)C | 0 |
| CC=CC | 0 |
| CC(CCOC(=O)C(=C)C)OC(=O)C(=C)C | 0 |
| OC(COCCCCOCC(O)COC(=O)C=C)COC(=O)C=C | 0 |
| CC(=C)C(=O)OCCCCOC(=O)C(=C)C | 0 |
| Cc1ccc(N=Nc2c(O)c(cc3ccccc23)C(=O)O)c(c1)S(=O)(=O)O | 0 |
| CC(=O)C(N=Nc1ccc(cc1[N+](=O)[O-])S(=O)(=O)O)C(=O)Nc2ccccc2Cl | 0 |
| Cc1ccccc1NC(=O)CC(=O)CN=Nc2ccc(cc2[N+](=O)[O-])S(=O)(=O)O | 0 |
| NNC(=O)NN | 0 |
| C1CCCC=CCC1 | 0 |
| C1CCC(CC1)c2ccccc2 | 0 |
| NCC1CCCC(CN)C1 | 0 |
| C=CCOC(=O)OCCOCCOC(=O)OCC=C | 0 |
| CCCCCCCCCCOC(=O)CCCCCCCC=CCCCCCCCC | 0 |
| OC1CCCCC1 | 0 |
| CCCCOC(=O)C=CC(=O)OCCCC | 0 |
| CCCCCCCCCCCCOC(=O)C=CC(=O)OCCCCCCCCCCCC | 0 |
| CCOC(=O)C1=NN(C(=O)C1N=Nc2ccc(cc2Cl)c3ccc(N=NC4C(=O)N(N=C4C(=O)OCC)c5ccccc5)c(Cl)c3)c6ccccc6 | 0 |
| CCc1ccccc1CC | 0 |
| CC(C)OC(=O)CCCCC(=O)OC(C)C | 0 |
| CC(C)CCCCCCCCCCOC(=O)CCCCC(=O)OCCCCCCCCCCC(C)C | 0 |
| COC(=O)CC(=O)OC | 0 |
| COC(=O)c1ccc2cc(ccc2c1)C(=O)OC | 0 |
| CCCCCCCCCCCCCCCCCCP(=O)(OC)OC | 0 |
| COC(=O)CCCCCCCCC(=O)OC | 0 |
| CCCCCCCCCCCCCC[N+](C)(C)[O-] | 0 |
| CCCCCCCCC=CCCCCCCCCNC(=O)CC(C(=O)O)S(=O)(=O)O | 0 |
| Nc1ccc(N=Nc2ccc(cc2)N=Nc3c(N)c4c(O)c(N=Nc5ccc(cc5)N=Nc6ccc(N)cc6N)c(cc4cc3S(=O)(=O)O)S(=O)(=O)O)c(N)c1 | 0 |
| CCCCCCCCCCCCCCOC(=O)C=CC(=O)OCCCCCCCCCCCCCC | 0 |
| CCCCCCCCCCCCCCCCCCCCCCOC(=O)CCCCCCCCCCCCCCCCCCCCC | 0 |
| CCCCCCCCCCCCS | 0 |
| CCCCCCCCCCCCCCCCCCCCCC(=O)O | 0 |
| CCCCCCCCCCCC#N | 0 |
| CC(=C)C(=O)OCCOCCOCCOC(=O)C(=C)C | 0 |
| CCS | 0 |
| CCCCCCCCCCCCOC(=O)CCCCCCCC=CCCCCCCCC | 0 |
| CCCCCCCCCCCCOC(=O)C=C | 0 |
| CCCCCCCCCCCC[N+](C)(C)[O-] | 0 |
| CC | 0 |
| CCOC(=O)CC1(C)OCCO1 | 0 |
| CCOC=C | 0 |
| CCOC(=O)c1ccc(O)cc1 | 0 |
| CCCC(C)C(=O)OCC | 0 |
| OC(=O)C=O | 0 |
| CC(O)C#CC(C)O | 0 |
| CCCCCCCCCCCCCCCCOC(=O)C=C | 0 |
| CCCCCCN | 0 |
| C=CC(=O)OCCCCCCOC(=O)C=C | 0 |
| CCCCCCCCCCCCCCCCCCCC(=O)O | 0 |
| CCCCCC(=O)O | 0 |
| CC(C)CCCCCOC(=O)CCS | 0 |
| CC(=C)c1ccccc1 | 0 |
| F[P-](F)(F)(F)(F)F | 0 |
| C | 0 |
| COC=C | 0 |
| CC(=C)C#N | 0 |
| COC(=O)CC(=O)C | 0 |
| COC(=O)C=Cc1ccccc1 | 0 |
| CCCCCCCCCC(=O)OC | 0 |
| CCCCCC(=O)OC | 0 |
| CCCCCCCCCCCCCC(=O)OC | 0 |
| CCCCCCCCCCCC(=O)OC | 0 |
| CCCCCCCC(=O)OC | 0 |
| CCCCCCCCCCCCCCCC(=O)OC | 0 |
| CCCCN(CCCC)C(=S)SCSC(=S)N(CCCC)CCCC | 0 |
| C[N+](=O)[O-] | 0 |
| CCCCCCCCCCCCCCCCCC(=O)OC | 0 |
| CCCCCCCCCC=CCCCCCCC(=O)OCCCCCCCCC=CCCCCCCCC | 0 |
| NCCNCCNCCNCCNCCN | 0 |
| CCCCCCCCC=CCCCCCCCCCCCC(=O)OCCCCCCCCC=CCCCCCCCC | 0 |
| CC[N+](=O)[O-] | 0 |
| CCCCCCCCS | 0 |
| CCCCCCCCCCCCCCCCCCOC(=O)C=C | 0 |
| CCCCCCCCN | 0 |
| CCC=CC#N | 0 |
| O=C1CCCO1 | 0 |
| CCCCCCCCCCCCCCCC(=O)O | 0 |
| O[PH+]=O | 0 |
| CCCCCO | 0 |
| OP(=O)(O)F | 0 |
| CC(COCC(C)OC(=O)C=C)OC(=O)C=C | 0 |
| O[P+](=O)O | 0 |
| CCCN | 0 |
| OC(=O)C(F)(F)F | 0 |
| CC(=O)OCC(COC(=O)C)OC(=O)C | 0 |
| CC(CC(=O)OCC(COC(=O)CC(C)CC(C)(C)C)OC(=O)CC(C)CC(C)(C)C)CC(C)(C)C | 0 |
| OCCCO | 0 |
| CC(C)S | 0 |
| CCCOC(=O)c1ccc(O)cc1 | 0 |
| Oc1ccccc1O | 0 |
| C1CCNC1 | 0 |
| OCC(O)C(O)C(O)C(O)C(O)C(=O)O | 0 |
| CCCCCCCCCCCCN(CCC(=O)O)CCC(=O)O | 0 |
| Cc1cc(Nc2cc(c(N)c3C(=O)c4ccccc4C(=O)c23)S(=O)(=O)O)cc(c1C)S(=O)(=O)NCCO | 0 |
| CC(C)Cc1cc2ccccc2c(c1CC(C)C)S(=O)(=O)O | 0 |
| CN(C1CCCCC1)S(=O)(=O)c2ccccc2N=Nc3c(N)ccc4cc(cc(O)c34)S(=O)(=O)O | 0 |
| OS(=O)(=O)c1ccc(Nc2nc(Cl)nc(Cl)n2)cc1 | 0 |
| OS(=O)(=O)C=C | 0 |
| OC(=O)c1ccc[n+](Cc2ccccc2)c1 | 0 |
| CS | 0 |
| OP=O | 0 |
| OS(=O)(=O)CC=C | 0 |
| CCCCCCCCCCCCCCCCCC(=O)OCC(O)CO | 0 |
| Cc1cc(N=Nc2c(O)c(cc3ccccc23)C(=O)O)c(cc1Cl)S(=O)(=O)O | 0 |
| Cc1cc(N=Nc2c(O)ccc3ccccc23)c(cc1Cl)S(=O)(=O)O | 0 |
| Cc1c(Cl)cc(cc1S(=O)(=O)O)N=Nc2c(O)c(cc3ccccc23)C(=O)O | 0 |
| CC(C)C(=O)OOC(C)(C)C | 0 |
| CC(C)(C)OOC(=O)C(C)(C)C | 0 |
| FS(F)(F)(F)(F)F | 0 |
| CC(C)(C)OOC(C)(C)c1ccccc1 | 0 |
| CCCCCCCCCCCCCCOC(=O)CCCCCCCC=CCCCCCCCC | 0 |
| CCCCCCCCCCCCCCOC(=O)CCCCCCCCCCCCC | 0 |
| CCCCCCCCCCCCCCOC(=O)C=C | 0 |
| CC12CCCC=C1C(=O)OC2=O | 0 |
| COC(=O)c1ccc(C(=O)OC)c(c1)N=NC(C(=O)C)C(=O)Nc2ccc(NC(=O)C(N=Nc3cc(ccc3C(=O)OC)C(=O)OC)C(=O)C)cc2 | 0 |
| O=C1CCCCO1 | 0 |
| CC(O)(P(=O)(O)O)P(=O)(O)O | 0 |
| CC(C)c1ccc(C)cc1O | 0 |
| OC(=O)CCCCCCCCCCCC(=O)O | 0 |
| OCC1CCC2C3CC(CC3CO)C12 | 0 |
| CC(=C)C(=O)NCCC[N+](C)(C)C | 0 |
| CCCCCCCCOC(=O)c1ccc(C(=O)OCCCCCCCC)c(c1)C(=O)OCCCCCCCC | 0 |
| CCCCCCCCCCC=O | 0 |
| CC(C)(C)CCCCCC(=O)OC=C | 0 |
| CCCCOP(=S)(S)OCCCC | 0 |
| CCOC(=O)C1=NN(c2ccc(Cl)cc2Cl)C(C)(C1)C(=O)OCC | 0 |
| Nc1cccc(N)c1 | 0 |
| Nc1nc(N)c2nc3c(N)nc(N)nc3nc2n1 | 0 |
| CC(C)OC(=O)c1ccc(C)c(c1)N=Nc2c(O)c(cc3ccccc23)C(=O)Nc4cc(Cl)c(NC(=O)c5cc6ccccc6c(N=Nc7cc(ccc7C)C(=O)OC(C)C)c5O)cc4Cl | 0 |
| Nc1ccc([N+](=O)[O-])cc1 | 1 |
| Cc1cc(=O)[nH]o1 | 1 |
| C=CC1CC=CCC1 | 1 |
| O=Cc1ccccc1 | 1 |
| COC(=O)c1c(Cl)nn(C)c1S(=O)(=O)NC(=O)Nc1nc(OC)cc(OC)n1 | 1 |
| [N-]=[N+]=N[C@]1(CO)O[C@@H](n2ccc(N)nc2=O)[C@@H](F)[C@@H]1O | 1 |
| CCC/C(=N\OCC)C1=C(O)CC(C2CCCSC2)CC1=O | 1 |
| Nc1ccc(Oc2ccc(N)cc2)cc1 | 1 |
| ClB(Cl)Cl | 1 |
| O=C(NC(=O)c1c(F)cccc1F)Nc1cc(Cl)c(OC(F)(F)C(F)C(F)(F)F)cc1Cl | 1 |
| CS(=O)(=O)c1ccc(C(=O)C2C(=O)CCCC2=O)c([N+](=O)[O-])c1 | 1 |
| O=c1c(C2CC(c3ccc(-c4ccc(Br)cc4)cc3)Cc3ccccc32)c(O)sc2ccccc12 | 1 |
| CCOc1ccc([Si](C)(C)CCCc2ccc(F)c(Oc3ccccc3)c2)cc1 | 1 |
| C#CCOC(=O)[C@@H](C)Oc1ccc(Oc2ncc(Cl)cc2F)cc1 | 1 |
| CC(C)=CCC/C(C)=C/CO | 0 |
| CCC(C)n1ncn(-c2ccc(N3CCN(c4ccc(O)cc4)CC3)cc2)c1=O | 1 |
| C=C[Si](OCCOC)(OCCOC)OCCOC | 1 |
| C1CC2OC2CC1C1CO1 | 1 |
| C=CCOCC1CO1 | 1 |
| CC(C)(C)C(O)(CCc1ccc(Cl)cc1)Cn1cncn1 | 1 |
| CNC1C(O)C(OC2C(N)CC(N)C(OC3OC(C(C)O)C(O)C(O)C3N)C2O)OCC1(C)O | 1 |
| CC1OC(C)OC(C)OC(C)O1 | 1 |
| COCC(C)OC(C)=O | 1 |
| Nc1nc(N)nc(N)n1 | 1 |
| CCn1ncc(C(=O)c2ccc(S(C)(=O)=O)c(OCCOC)c2C)c1OC(C)OC(=O)OC | 1 |
| COc1ccc(/C(=C/C(=O)N2CCOCC2)c2ccc(Cl)cc2)cc1OC | 1 |
| C1OCOCO1 | 1 |
| OB(O)O | 1 |
| ClCCOCCCl | 1 |
| S=C(S)NCCNC(=S)S | 1 |
| CCCCOCCO | 1 |
| COCCOCCO | 1 |
| CCC(C)c1cc(N=Nc2ccc([N+](=O)[O-])cc2)cc(C(C)CC)c1O | 1 |
| CC[C@H]1OC(=O)[C@H](C)[C@@H](O[C@H]2C[C@@](C)(OC)[C@@H](O)[C@H](C)O2)[C@H](C)[C@@H](O[C@@H]2O[C@H](C)C[C@H](N(C)C)[C@H]2O)[C@](C)(O)C[C@@H](C)C(=O)[C@H](C)[C@@H](O)[C@]1(C)O | 0 |
| CNC(=O)Oc1ccccc1OC(C)C | 1 |
| COCc1cnc(C2=NC(C)(C(C)C)C(=O)N2)c(C(=O)O)c1 | 1 |
| O=C1NC(=O)c2cc(Cl)c3c4c(Cl)cc5c6c(cc(Cl)c(c7c(Cl)cc1c2c73)c64)C(=O)NC5=O | 1 |
| CCCN(CC)CC1COC2(CCC(C(C)(C)C)CC2)O1 | 1 |
| CCC(Cc1ccccc1)(C(=O)c1ccc(N2CCOCC2)cc1)N(C)C | 1 |
| CC1=CC(=O)N(Cc2cccc(CN3C(=O)C=C(C)C3=O)c2)C1=O | 1 |
| Oc1ccc(Cl)cc1Cc1ccccc1 | 1 |
| Cc1ccc([N+](=O)[O-])cc1[N+](=O)[O-] | 1 |
| COB(OC)OC | 1 |
| CC1COCc2cc3c(cc21)C(C)(C)C(C)C3(C)C | 1 |
| CCNc1nc(Cl)nc(NCC)n1 | 1 |
| CCCCC/C(C=O)=C/c1ccccc1 | 0 |
| c1ccc(OCC2CO2)cc1 | 1 |
| OOC1(OOC2(O)CCCCC2)CCCCC1 | 1 |
| CON(C(=O)c1cn(C)nc1C(F)F)C(C)Cc1c(Cl)cc(Cl)cc1Cl | 1 |
| CCCCC(C=O)CC | 0 |
| CC1=NNC(=O)N(/N=C/c2cccnc2)C1 | 1 |
| CC1(C)CCC(Cc2ccc(Cl)cc2)C1(O)Cn1cncn1 | 1 |
| CC(C)C1CCC(Cc2ccc(Cl)cc2)C1(O)Cn1cncn1 | 1 |
| CC(C)c1ccc(Cl)cc1CN(C(=O)c1c(C(F)F)nn(C)c1F)C1CC1 | 1 |
| ClC(Cl)=C(Cl)Cl | 0 |
| CC(=O)[C@H]1CC[C@H]2[C@@H]3CC[C@@H]4C[C@H](O)CC[C@]4(C)[C@H]3CC[C@]12C | 0 |
| CN(C)c1ccc(C(c2ccccc2)c2ccc(N(C)C)cc2)cc1 | 1 |
| O=C(O)[C@@H]1C[C@@H](C2CCCCC2)CN1 | 1 |
| Oc1c(I)cc(Cl)c2cccnc12 | 1 |
| O=BOB=O | 1 |
| C=C[C@H]1CN2CC[C@H]1C[C@H]2[C@H](O)c1ccnc2ccc(OC)cc12 | 0 |
| SCCSCC(CS)SCCS | 1 |
| COc1ccc(C(=O)c2ccccc2)c(O)c1 | 0 |
| C/C=C/C(=O)Oc1c(C(C)CCCCCC)cc([N+](=O)[O-])cc1[N+](=O)[O-] | 1 |
| CC1(C)CC/C(=C\c2ccc(Cl)cc2)C1(O)Cn1cncn1 | 1 |
| Clc1cc(-c2cc(Cl)c(Cl)c(Cl)c2)cc(Cl)c1Cl | 1 |
| Fc1ccc([C@@]2(Cn3cncn3)O[C@@H]2c2ccccc2Cl)cc1 | 1 |
| O=C1O[C@H]([C@@H](O)CO)C([O-])=C1O | 0 |
| O[C@@](Cn1cnnn1)(c1ccc(F)cc1F)C(F)(F)c1ccc(-c2ccc(OCC(F)(F)F)cc2)cn1 | 1 |
| CC(=NC#N)N(C)Cc1ccc(Cl)nc1 | 1 |
| CC(C)(c1ccc(O)cc1)c1cccc(C(C)(C)c2ccc(O)cc2)c1 | 1 |
| CCOC(=O)Nc1cccc(OC(=O)Nc2ccccc2)c1 | 1 |
| CN(C)C(=S)SSC(=S)N(C)C | 0 |
| CCCCC(=O)N(Cc1ccc(-c2ccccc2-c2nn[nH]n2)cc1)[C@H](C(=O)O)C(C)C | 1 |
| CCCC(=O)c1c(C)cc(C)c(C2CC(=O)C(/C(CC)=N/OCC)=C(O)C2)c1C | 1 |
| CCC/C(=N\OCC(C)Oc1ccc(Cl)cc1)C1=C(O)CC(C2CCCSC2)CC1=O | 1 |
| N#Cc1ccc(C/C(=N\NC(=O)Nc2ccc(OC(F)(F)F)cc2)c2cccc(C(F)(F)F)c2)cc1 | 1 |
| O=C(O)/C=C/C(=O)O | 0 |
| CS(=O)(=O)c1cc(C(F)(F)F)ccc1C(=O)c1cnoc1C1CC1 | 1 |
| CN1CCC[C@@H]1Cc1c[nH]c2ccc(Br)cc12 | 1 |
| CCN1CCOC1(C)CCC(C)C | 1 |
| Cc1cc(C)nc(NC(=O)NS(=O)(=O)c2ccccc2C(=O)OC2COC2)n1 | 1 |
| Nc1ccc(S(=O)(=O)Nc2ccccn2)cc1 | 1 |
| Cc1ccn[nH]1 | 1 |
| Oc1ccc(C(c2ccc(O)cc2)(C(F)(F)F)C(F)(F)F)cc1 | 1 |
| CCOP(=S)(OCC)O/N=C(\C#N)c1ccccc1 | 1 |
| CCC(C)(C)C(=O)OC1=C(c2ccc(Cl)cc2Cl)C(=O)OC12CCCCC2 | 1 |
| S=c1[nH]c2ccccc2s1 | 0 |
| CNC(=O)/C(=N/OC)c1ccccc1COc1cc(C)ccc1C | 1 |
| CC/C(=N\OC/C=C/Cl)C1=C(O)CC(C2CCOCC2)CC1=O | 1 |
| Nc1ccc(C(=O)O)cc1 | 0 |
| C1CN1 | 1 |
| Cc1cccc(NC(=O)C2=Cc3ccccc3C(=NNc3ccc4c(c3)C(=O)c3cc(NN=C5C(=O)C(C(=O)Nc6ccccc6Cl)=Cc6ccccc65)ccc3-4)C2=O)c1 | 1 |
| C[C@@H]1C[C@H]2[C@@H]3C[C@H](F)C4=CC(=O)C=C[C@]4(C)[C@H]3[C@@H](O)C[C@]2(C)[C@H]1C(=O)CO | 1 |
| CN1COCN(Cc2cnc(Cl)s2)/C1=N/[N+](=O)[O-] | 1 |
| C[C@]12C=CC(=O)C=C1CC[C@@H]1C2=CC[C@]2(C)C(=O)CC[C@@H]12 | 1 |
| Cc1ccc(NC(=O)N(C)C)cc1Cl | 1 |
| C=C[Si](C)(O/N=C(\C)CC(C)C)O/N=C(\C)CC(C)C | 1 |
| Cl/C=C/Cl | 0 |
| CCN1CCCC1CNC(=O)c1cc(S(N)(=O)=O)ccc1OC | 1 |
| CC(c1ccccc1)(c1ccc(O)cc1)c1ccc(O)cc1 | 0 |
| CCCCCCCCCCCCCCCCCCOP(=O)([O-])OC1CC[N+](C)(C)CC1 | 1 |
| CC1CN(C2CCCCCCCCCCC2)CC(C)O1 | 1 |
| CCNC(=O)[C@@H](C)OC(=O)Nc1ccccc1 | 1 |
| CC1=C(/C=C/C(C)=C/C=C/C(C)=C/C=C/C=C(\C)C=O)C(C)(C)CCC1 | 1 |
| CC(C)(c1ccc(OCC2CO2)cc1)c1ccc(OCC2CO2)cc1 | 0 |
| N#Cc1cc(I)c(O)c(I)c1 | 1 |
| N#Cc1cc(Br)c(O)c(Br)c1 | 1 |
| CCCCCCCC(=O)Oc1c(Br)cc(C#N)cc1Br | 1 |
| SCCSC(CS)CSCC(CS)SCCS | 1 |
| O=C1Cc2cc(Cl)ccc2N1 | 1 |
| O=C(O)CN(CCCN(CC(=O)O)CC(=O)O)CC(=O)O | 1 |
| COc1cc2nc[nH]c(=O)c2cc1OCCCN1CCOCC1 | 1 |
| C[C@@H](Oc1ccc(Oc2cnc3cc(Cl)ccc3n2)cc1)C(=O)OCC1CCCO1 | 1 |
| CC(C)C(N)C(N)=O | 1 |
| CCOC(=O)OC1=C(c2cc(C)ccc2C)C(=O)NC12CCC(OC)CC2 | 1 |
| Oc1c(F)cccc1Cl | 1 |
| CN/C(=N\[N+](=O)[O-])NCc1cnc(Cl)s1 | 1 |
| CC1CNC(=S)N1 | 1 |
| C[C@@H]1C[C@H]2[C@@H]3C[C@H](F)C4=CC(=O)C=C[C@]4(C)[C@@]3(F)[C@@H](O)C[C@]2(C)[C@@]1(O)C(=O)CO | 1 |
| CC(C)(c1ccc(O)cc1)c1ccc(C(C)(C)c2ccc(O)cc2)cc1 | 0 |
| CCSC(=O)N1CCCCCC1 | 1 |
| CC(C)[C@@H]1CC[C@@H](C)C[C@H]1O | 0 |
| COc1ccc2cc([C@H](C)C(=O)O)ccc2c1 | 1 |
| O=C(OCc1ccccc1)C(Br)CCBr | 1 |
| CCOP(=S)(OCC)SCn1c(=O)oc2cc(Cl)ccc21 | 1 |
| ClC1(Cl)C2(Cl)C3(Cl)C4(Cl)C(Cl)(Cl)C5(Cl)C3(Cl)C1(Cl)C5(Cl)C24Cl | 1 |
| O=C(NCc1ncc(C(F)(F)F)cc1Cl)c1c(Cl)cccc1Cl | 1 |
| CCCCOCC1CO1 | 1 |
| Cc1ccc2nc3sc(=O)sc3nc2c1 | 1 |
| CCc1cc(C)cc(CC)c1-c1c(OC(=O)C(C)(C)C)n2n(c1=O)CCOCC2 | 1 |
| CC(C)N1C(=O)c2ccccc2NS1(=O)=O | 1 |
| BrC1(Br)CCCCCCCCCC(Br)(Br)C1(Br)Br | 1 |
| CCCCCCCCCc1ccccc1OP(Oc1ccccc1CCCCCCCCC)Oc1ccccc1CCCCCCCCC | 1 |
| CC1(C)S[C@@H]2[C@H](NC(=O)[C@H](N)c3ccc(O)cc3)C(=O)N2[C@H]1C(=O)O | 0 |
| NC1=C(Cl)C(=O)c2ccccc2C1=O | 1 |
| Cc1cn[nH]c1C | 1 |
| CCN(CC)C(=O)SCc1ccc(Cl)cc1 | 1 |
| O=c1oc2ccccc2c(O)c1C(CC(O)c1ccc(-c2ccc(Br)cc2)cc1)c1ccccc1 | 1 |
| C[C@H]1C[C@H]2[C@@H]3CC[C@](O)(C(=O)COC(=O)CCC(=O)O)[C@@]3(C)C[C@H](O)[C@@H]2[C@@]2(C)C=CC(=O)C=C12 | 1 |
| OC(O)C(Cl)(Cl)Cl | 0 |
| C[C@]12CC[C@H]3[C@@H](CCC4=CC(=O)CC[C@@]43C)[C@@H]1CC[C@@H]2C(=O)O | 1 |
| BrC1CCC(Br)C(Br)CCC(Br)C(Br)CCC1Br | 1 |
| CN(C)C(=O)Nc1ccc(Cl)c(Cl)c1 | 0 |
| CCOP(=S)(OCC)Oc1cc(C)nc(C(C)C)n1 | 1 |
| CS(=O)(=O)c1ccc(C(=O)C2C(=O)CCCC2=O)c(Cl)c1COCC(F)(F)F | 1 |
| CCOCN(C(=O)CCl)c1c(C)cccc1CC | 1 |
| CCN(CC)CCNC(=O)c1cc(Cl)c(N)cc1OC | 0 |
| CC(C)NC(=O)N1CC(=O)N(c2cc(Cl)cc(Cl)c2)C1=O | 1 |
| C[C@H]1C[C@H]2[C@@H]3CCC4=CC(=O)C=C[C@]4(C)[C@@]3(F)[C@@H](O)C[C@]2(C)[C@@]1(O)C(=O)CO | 1 |
| COCCO[Si](CCCl)(OCCOC)OCCOC | 1 |
| CCCCCCCC(=O)Oc1c(I)cc(C#N)cc1I | 1 |
| CCC(CC)Nc1c([N+](=O)[O-])cc(C)c(C)c1[N+](=O)[O-] | 1 |
| C/C=C/c1ccc(OC)cc1 | 0 |
| CC(C)NC[C@@H](COC1=CC=CC2=CC=CC=C21)O | 1 |
| C[C@@]12CCC[C@H]1[C@@H]1CC[C@H]3CCCC[C@]3(C)[C@H]1CC2 | 0 |
| Cc1ncc([N+](=O)[O-])n1CCO | 1 |
| CN1CC[C@]23c4c5ccc(O)c4O[C@H]2C(=O)CC[C@H]3[C@H]1C5 | 1 |
| CC[C@H]1CC[C@H]2[C@@H]3CC[C@@H]4CCCC[C@]4(C)[C@H]3CC[C@]12C | 0 |
| Cc1cc(O)c(C)c(O)c1 | 0 |
| CC(O)C(=O)O | 0 |
| C[C@]12C[C@H](O)[C@H]3[C@@H](CCC4=CC(=O)CC[C@@]43C)[C@@H]1CC[C@]2(O)C(=O)CO | 1 |
| C[C@]12CC[C@@H]3c4ccc(O)cc4CC[C@H]3[C@@H]1CC[C@@H]2O | 1 |
| CCCCOCCOCCOCc1cc2c(cc1CCC)OCO2 | 0 |
| O=[N+]([O-])c1ccc(O)c([N+](=O)[O-])c1 | 1 |
| CN1[C@H]2C[C@H](OC(=O)[C@H](CO)c3ccccc3)C[C@@H]1[C@H]1O[C@@H]21 | 0 |
| ClC[C@H]1CO1 | 1 |
| CC(=O)[C@H]1CC[C@H]2[C@@H]3CC[C@H]4C[C@H](O)CC[C@]4(C)[C@H]3CC[C@]12C | 0 |
| CC(=O)[C@H]1CC[C@H]2[C@@H]3CC[C@H]4C[C@@H](O)CC[C@]4(C)[C@H]3CC[C@]12C | 0 |
| CCOC(N)=O | 1 |
| COc1ccc([C@@H]2CC(=O)c3c(O)cc(O[C@@H]4O[C@H](CO[C@@H]5O[C@@H](C)[C@H](O)[C@@H](O)[C@H]5O)[C@@H](O)[C@H](O)[C@H]4O)cc3O2)cc1O | 0 |
| COc1ccc(-c2cc(=O)c3c(O)cc(O[C@@H]4O[C@H](CO[C@@H]5O[C@@H](C)[C@H](O)[C@@H](O)[C@H]5O)[C@@H](O)[C@H](O)[C@H]4O)cc3o2)cc1O | 0 |
| Clc1ccc(C(Cl)(Cl)Cl)cc1 | 1 |
| CC1(C)C(C=C(Cl)Cl)C1C(=O)OC(C#N)c1cccc(Oc2ccccc2)c1 | 1 |
| O=[N+]([O-])C(Br)(CO)CO | 0 |
| O=C(O)[C@H](O)[C@@H](O)[C@H](O)[C@H](O)CO | 0 |
| N[C@@H](CS)C(=O)O | 0 |
| CC1(C)[C@@H](C=C(Br)Br)[C@H]1C(=O)O[C@H](C#N)c1cccc(Oc2ccccc2)c1 | 1 |
| O=C(O)c1ccccc1Nc1cccc(C(F)(F)F)c1 | 0 |
| C[C@]12CC[C@@H]3c4ccc(O)cc4CC[C@H]3[C@@H]1CCC2=O | 1 |
| C[C@]12CC[C@H]3[C@@H](CC[C@H]4C[C@H](O)CC[C@@]43C)[C@@H]1CCC2=O | 0 |
| C[C@]12CC[C@H]3[C@@H](CC[C@@H]4C[C@H](O)CC[C@]34C)[C@@H]1CCC2=O | 0 |
| CCNC(=O)[C@@H]1CCCN1C(=O)[C@H](CCCN=C(N)N)NC(=O)[C@H](CC(C)C)NC(=O)[C@@H](CC(C)C)NC(=O)[C@H](Cc1ccc(O)cc1)NC(=O)[C@H](CO)NC(=O)[C@H](Cc1c[nH]c2ccccc12)NC(=O)[C@H](Cc1cnc[nH]1)NC(=O)[C@@H]1CCC(=O)N1 | 1 |
| Clc1cccc(Cl)c1 | 0 |
| COCCCC/C(=N\OCCN)c1ccc(C(F)(F)F)cc1 | 0 |
| COP(=S)(OC)Oc1ccc(SC)c(C)c1 | 1 |
| CC(=O)C[C@@H](c1ccccc1)c1c(O)c2ccccc2oc1=O | 1 |
| CC(=O)C[C@H](c1ccccc1)c1c(O)c2ccccc2oc1=O | 1 |
| C[Si]1(C)O[Si](C)(C)O[Si](C)(C)O[Si](C)(C)O1 | 1 |
| C[C@]12CC=C3C4=C(CC[C@H]3[C@@H]1CCC2=O)CC1(CC4)OCCO1 | 1 |
| CC1c2cccc(O)c2C(O)=C2C(=O)C3(O)C(O)=C(C(N)=O)C(=O)C(N(C)C)C3C(O)C21 | 1 |
| CC/C(=C(/CC)\C1=CC=C(C=C1)O)/C2=CC=C(C=C2)O | 1 |
| CCCCCCC(=O)Oc1c(Br)cc(C#N)cc1Br | 1 |
| OCC(O)CO | 0 |
| N[C@@H](CC(=O)O)C(=O)O | 0 |
| NC(=O)CC[C@H](N)C(=O)O | 0 |
| N[C@@H](CCC(=O)O)C(=O)O | 0 |
| N[C@@H](CSSC[C@H](N)C(=O)O)C(=O)O | 0 |
| CN(CC(=O)O)C(=N)N | 0 |
| OC[C@@H]1CO1 | 1 |
| CC(=O)c1c(C)c2cnc(Nc3ccc(N4CCNCC4)cn3)nc2n(C2CCCC2)c1=O | 1 |
| CC(O)CO | 0 |
| Cc1cc(C)nc(NS(=O)(=O)c2ccc(N)cc2)n1 | 0 |
| Oc1cccc2ncccc12 | 0 |
| CC(C)CCC[C@@H](C)[C@H]1CC[C@H]2[C@@H]3CC=C4C[C@@H](O)CC[C@]4(C)[C@H]3CC[C@@]21C | 0 |
| CCNC(=O)NC(=O)/C(C#N)=N/OC | 1 |
| Oc1ccc2ncccc2c1 | 0 |
| Oc1cnc2ccccc2c1 | 0 |
| Oc1ccc2cccnc2c1 | 0 |
| CNC1=Nc2ccc(Cl)cc2C(c2ccccc2)=[N+]([O-])C1 | 1 |
| Cn1c(=O)c2[nH]cnc2n(C)c1=O | 1 |
| OC[C@H](O)[C@@H](O)[C@H](O[C@@H]1O[C@H](CO)[C@H](O)[C@H](O)[C@H]1O)[C@H](O)CO | 0 |
| O=C[C@H](O)[C@@H](O)[C@H](O)CO | 0 |
| Cc1c(C)c2c(c(C)c1O)CC[C@@](C)(CCC[C@H](C)CCC[C@H](C)CCCC(C)C)O2 | 0 |
| CC(=O)OC/N=[N+](/C)[O-] | 1 |
| CNC[C@H](O)c1cccc(O)c1 | 0 |
| Cc1ncc2n1-c1ccc(Cl)cc1C(c1ccccc1F)=NC2 | 1 |
| O=C(O)c1cccnc1 | 0 |
| OC(c1ccc(Cl)cc1)(c1cncnc1)c1ccccc1Cl | 1 |
| Cc1cccc([N+](=O)[O-])c1[N+](=O)[O-] | 1 |
| CCCC1COC(Cn2cncn2)(c2ccc(Cl)cc2Cl)O1 | 1 |
| Cc1c([N+](=O)[O-])cccc1[N+](=O)[O-] | 1 |
| Cc1ccc([N+](=O)[O-])c([N+](=O)[O-])c1 | 1 |
| O=C1C(Cl)C(CCl)CN1c1cccc(C(F)(F)F)c1 | 1 |
| O=CC1CCCSC1 | 1 |
| Nc1ncn[nH]1 | 1 |
| Cc1cc([N+](=O)[O-])cc([N+](=O)[O-])c1 | 1 |
| Cc1cc([N+](=O)[O-])ccc1[N+](=O)[O-] | 1 |
| Oc1ccc(Cc2ccc(O)cc2)cc1 | 0 |
| CCc1ccc(C)cc1 | 0 |
| CN=C=O | 1 |
| Nc1ccccc1 | 1 |
| NC(N)=S | 1 |
| CSCC[C@H](N)C(=O)O | 0 |
| N[C@@H](Cc1ccccc1)C(=O)O | 0 |
| O[C@H]1CO[C@@H]2[C@H](O)CO[C@H]12 | 0 |
| CC(=O)N1CCN(c2ccc(OC[C@H]3CO[C@](Cn4ccnc4)(c4ccc(Cl)cc4Cl)O3)cc2)CC1 | 1 |
| CN1CCCC1c1cccnc1 | 1 |
| NC(=O)c1ccccc1O | 0 |
| CCCC(Cn1cncn1)c1ccc(Cl)cc1Cl | 1 |
| C[C@H]1C[C@H](C)C(=O)[C@H]([C@H](O)CC2CC(=O)NC(=O)C2)C1 | 1 |
| CC1(C)CN=C(NN=C(/C=C/c2ccc(C(F)(F)F)cc2)/C=C/c2ccc(C(F)(F)F)cc2)NC1 | 1 |
| CC(Cc1ccc(C(C)(C)C)cc1)CN1C[C@H](C)O[C@H](C)C1 | 1 |
| ClC(Cl)Cl | 1 |
| CC(C)CC(C)(c1ccc(O)cc1)c1ccc(O)cc1 | 1 |
| CC(=O)O[C@H]1C[C@@]2(C)[C@@H](C[C@@H](O)[C@H]3[C@@]4(C)CC[C@@H](O)[C@@H](C)[C@@H]4CC[C@@]32C)/C1=C(\CCC=C(C)C)C(=O)O | 0 |
| NC(=O)C[C@H](N)C(=O)O | 0 |
| CCCO | 0 |
| c1ccccc1 | 0 |
| CC(Cl)(Cl)Cl | 0 |
| COc1ccc(/C=C/C(=O)OCCC(C)C)cc1 | 0 |
| CC(C)[C@H](N)C(=O)O | 0 |
| CC(O)C(N)C(=O)O | 0 |
| CN1CCCN(C)C1=O | 1 |
| COP(=S)(OC)SCN1C(=O)c2ccccc2C1=O | 1 |
| N[C@@H](Cc1c[nH]c2ccccc12)C(=O)O | 0 |
| CCB(CC)OC | 1 |
| CCCl | 1 |
| C=CCl | 0 |
| S=C=S | 1 |
| C=C(Cl)Cl | 0 |
| Cc1cc(C)c(C(=O)P(=O)(c2ccccc2)c2ccccc2)c(C)c1 | 1 |
| CC(C)(C)[C@@H](O)[C@@H](Cc1ccc(Cl)cc1)n1cncn1 | 1 |
| Cc1c(C)c2c(c(C)c1OC(=O)CCC(=O)O)CC[C@@](C)(CCC[C@H](C)CCC[C@H](C)CCCC(C)C)O2 | 0 |
| O=C1OC(c2ccc(O)cc2)(c2ccc(O)cc2)c2ccccc21 | 1 |
| CCC(C)(c1ccc(O)cc1)c1ccc(O)cc1 | 0 |
| ClC1=C(Cl)C(Cl)(Cl)C(Cl)=C1Cl | 0 |
| CC(=CC(C)(C)CC(C)(C)C)C | 0 |
| CCCCCOC(=O)c1ccccc1C(=O)OCCC(C)C | 1 |
| CCN(CC)c1cccc(N=Nc2sc([N+](=O)[O-])cc2C(C)=O)c1NC(C)=O | 1 |
| COc1cc(S(N)(=O)=O)cc(C)c1N=Nc1cc2c(O)cc(S(=O)(=O)O)cc2cc1Nc1nc(NCC(C)N)nc(NC(C)(N)COC=O)n1 | 1 |
| CCC(C)=O | 0 |
| ClC=C(Cl)Cl | 0 |
| NC(=O)CCl | 1 |
| CCCCOC(=O)[C@@H](C)Oc1ccc(Oc2ccc(C(F)(F)F)cn2)cc1 | 1 |
| C[C@H](O)C(=O)O | 0 |
| O=C(O)C(Cl)Cl | 1 |
| O=[N+]([O-])c1cc(C(F)(F)F)c(Cl)c([N+](=O)[O-])c1Nc1ncc(C(F)(F)F)cc1Cl | 1 |
| O=C(O)[C@@H]1Cc2ccccc2N1 | 1 |
| CCC(C)(C)C(=O)O[C@H]1C[C@@H](C)C=C2C=C[C@H](C)[C@H](CC[C@@H]3C[C@@H](O)CC(=O)O3)[C@H]21 | 1 |
| CCCCC(O)(Cn1cncn1)c1ccc(Cl)cc1Cl | 1 |
| Nc1ccc(S(=O)(=O)c2ccc(N)cc2)cc1 | 1 |
| CC(C)(OOC(C)(C)c1ccccc1)c1ccccc1 | 1 |
| O=C(O)CCNC(=O)c1ccc(/N=N/c2ccc(O)c(C(=O)O)c2)cc1 | 0 |
| CC(C)(CO)[C@@H](O)C(=O)NCCCO | 0 |
| CC(=O)CC(c1ccccc1)c1c(O)c2ccccc2oc1=O | 1 |
| CC/C(=C(/c1ccc(OCCN(C)C)cc1)c1cccc(O)c1)c1ccccc1 | 1 |
| CCOC(=O)CCN(SN(C)C(=O)Oc1cccc2c1OC(C)(C)C2)C(C)C | 1 |
| COc1cccc(-c2c(C)n(Cc3c(F)cccc3C(F)(F)F)c(=O)n(CC(NCCCC(=O)O)c3ccccc3)c2=O)c1F | 1 |
| CC[C@H]1OC(=O)[C@H](C)[C@@H](O[C@H]2C[C@@](C)(OC)[C@@H](O)[C@H](C)O2)[C@H](C)[C@@H](O[C@@H]2O[C@H](C)C[C@H](N(C)C)[C@H]2O)[C@](C)(O)C[C@@H](C)CN(C)[C@H](C)[C@@H](O)[C@]1(C)O | 0 |
| CC(=O)Nc1nc2c(ncn2COC(CO)CO)c(=O)[nH]1 | 1 |
| Oc1ccc(C2(c3ccc(O)cc3)CCCCC2)cc1 | 1 |
| CC(C)CCc1ccc(C(=O)[O-])c(C(=O)[O-])c1CCC(C)C | 1 |
| CC(c1ccccc1)c1ccc(O)cc1O | 0 |
| C[Si](Cn1cncn1)(c1ccc(F)cc1)c1ccc(F)cc1 | 1 |
| O=NN(c1ccccc1)c1ccccc1 | 0 |
| O=[N+]([O-])O[C@H]1CO[C@H]2[C@@H]1OC[C@H]2O[N+](=O)[O-] | 0 |
| NP(N)(=O)Nc1ccccc1[N+](=O)[O-] | 1 |
| c1ccc2c(N3CCNCC3)nsc2c1 | 1 |
| O[C@H]1[C@H](O)[C@@H](O)[C@H](O)[C@@H](O)[C@H]1O | 0 |
| Cc1cc(O)cc(C)c1Cl | 0 |
| CC(C)C1C2CCC1c1c(NC(=O)c3cn(C)nc3C(F)F)cccc12 | 1 |
| CCCCC(C#N)(Cn1cncn1)c1ccc(Cl)cc1 | 1 |
| Cc1ccccc1[N+](=O)[O-] | 1 |
| COc1ccccc1O | 0 |
| O=C1O[C@H](CO)[C@@H](O)[C@H](O)[C@H]1O | 0 |
| c1ccc2ccccc2c1 | 0 |
| O=S(=O)(O)c1ccc2ccc(S(=O)(=O)O)cc2c1 | 0 |
| C=CC(=O)NCO | 1 |
| O=C(O)c1cn(C2CC2)c2cc(F)c(F)cc2c1=O | 1 |
| O=C(c1ccc(F)c(F)c1Nc1ccc(I)cc1F)N1CC(O)([C@@H]2CCCCN2)C1 | 1 |
| CCCC(O)=C1C(=O)CC(C2CCCSC2)CC1=O | 1 |
| c1ccc2sc(SNC3CCCCC3)nc2c1 | 1 |
| Cc1ccccc1C | 1 |
| Cc1ccccc1O | 0 |
| Cc1ccccc1Cl | 1 |
| Clc1ccccc1Cl | 0 |
| c1ccc(C2CO2)cc1 | 1 |
| OCC(Br)CBr | 1 |
| O=C(O)[C@@H]1C[C@@H](c2ccccc2)CN1 | 1 |
| COc1ccc(C[C@@H]2c3cc(OC)c(OC)cc3CC[N@+]2(C)CCC(=O)OCCCCCOC(=O)CC[N@@+]2(C)CCc3cc(OC)c(OC)cc3[C@H]2Cc2ccc(OC)c(OC)c2)cc1OC | 0 |
| CCOP(=S)(OCC)Oc1ccc(Cl)cc1Cl | 1 |
| C=CCc1ccc(O)c(OC)c1 | 0 |
| NC(=O)NC1NC(=O)NC1=O | 0 |
| CCN(CC)C(=S)SSC(=S)N(CC)CC | 0 |
| Nc1cccc([N+](=O)[O-])c1 | 1 |
| CS(=O)(=O)c1ccc(C(=O)C2C(=O)CCCC2=O)c(Cl)c1 | 1 |
| O=[N+]([O-])c1cc(NCCO)c(O)c([N+](=O)[O-])c1 | 1 |
| COC(=O)c1ccc(O)cc1 | 0 |
| Cc1ccc(C(C)C)cc1 | 0 |
| Cc1ccc(N(C)C)cc1 | 0 |

**Table S2. The algorithm implementation and key hyperparameters of machine learming models.**

| **Algorithm** | **Implementation** | **Key Hyperparameters** |
| --- | --- | --- |
| Decision Tree | DecisionTreeClassifier (scikit-learn) | criterion={“gini”, “entropy”}; max_depth={3, 5, 7, 10}; min_samples_split={2, 5, 10} |
| k-Nearest Neighbors | KNeighborsClassifier (scikit-learn) | n_neighbors={3, 5, 7, 9}; weights={“uniform”, “distance”}; metric=“minkowski” (p=2) |
| Linear SVM | LinearSVC (scikit-learn) | C={0.01, 0.1, 1, 10}; loss={“hinge”, “squared_hinge”} |
| Naive Bayes | BernoulliNB (scikit-learn) | alpha={0.1, 0.5, 1.0} |
| Logistic Regression | LogisticRegression (solver=“liblinear”) | penalty={“l1”, “l2”}; C={0.01, 0.1, 1, 10} |
| Random Forest | RandomForestClassifier (scikit-learn) | n_estimators={100, 200, 500}; max_features={“sqrt”, “log2”}; max_depth={None, 10, 20} |
| AdaBoost | AdaBoostClassifier (scikit-learn) | n_estimators={50, 100, 200}; learning_rate={0.01, 0.1, 1.0} |
| GBDT | GradientBoostingClassifier (scikit-learn) | n_estimators={100, 200}; learning_rate={0.01, 0.1}; max_depth={3, 5} |
| Extra Trees | ExtraTreesClassifier (scikit-learn) | n_estimators={100, 200}; max_features={“sqrt”, “log2”} |
| LightGBM | LGBMClassifier (lightgbm) | num_leaves={31, 63}; n_estimators={100, 200}; learning_rate={0.01, 0.1} |
| XGBoost | XGBClassifier (xgboost) | max_depth={3, 5}; n_estimators={100, 200}; learning_rate={0.01, 0.1}; subsample={0.8, 1.0} |

## REFERENCES

Feng, H., Zhang, L., Li, S., Liu, L., Yang, T., Yang, P., et al. (2021). Predicting the reproductive toxicity of chemicals using ensemble learning methods and molecular fingerprints, Toxicol. Lett., 340, 4-14.

Jiang, C., Yang, H., Di, P., Li, W., Tang, Y., Liu, G. (2019). In silico prediction of chemical reproductive toxicity using machine learning. J. Appl. Toxicol. 39, 844-854.

Zhang, H., Shen, C., Liu, R., Mao, J., Liu, C., Mu, B. (2020). Developing novel in silico prediction models for assessing chemical reproductive toxicity using the naïve Bayes classifier method. J. Appl. Toxicol. 40, 1198-1209.

Ren, J.N., Chen, Q., Ye, H.Y., Cao, C., Guo, Y.M., Yang, J.R., et al. (2024). FGTN: fragment-based graph transformer network for predicting reproductive toxicity. Arch. Toxicol. 98, 4077-4092.
